# The Double-Cross of Benzotriazole-Based Polymers as Donors and Acceptors in Non-Fullerene Organic Solar Cells

**DOI:** 10.3390/molecules29153625

**Published:** 2024-07-31

**Authors:** Laura Crociani

**Affiliations:** Institute of Condensed Matter Chemistry and Energy Technologies, ICMATE, National Research Council of Italy, CNR, Corso Stati Uniti 4, 35127 Padua, Italy; laura.crociani@cnr.it

**Keywords:** benzotriazole, organic solar cell, conjugated polymers, photovoltaic properties

## Abstract

Organic solar cells (OSCs) are considered a very promising technology to convert solar energy to electricity and a feasible option for the energy market because of the advantages of light weight, flexibility, and roll-to-roll manufacturing. They are mainly characterized by a bulk heterojunction structure where a polymer donor is blended with an electron acceptor. Their performance is highly affected by the design of donor–acceptor conjugated polymers and the choice of suitable acceptor. In particular, benzotriazole, a typical electron-deficient penta-heterocycle, has been combined with various donors to provide wide bandgap donor polymers, which have received a great deal of attention with the development of non-fullerene acceptors (NFAs) because of their suitable matching to provide devices with relevant power conversion efficiency (PCE). Moreover, different benzotriazole-based polymers are gaining more and more interest because they are considered promising acceptors in OSCs. Since the development of a suitable method to choose generally a donor/acceptor material is a challenging issue, this review is meant to be useful especially for organic chemical scientists to understand all the progress achieved with benzotriazole-based polymers used as donors with NFAs and as acceptors with different donors in OSCs, in particular referring to the PCE.

## 1. Introduction

The continuous increase in global energy demand due to population growth and economic development cannot be any longer satisfied simply by traditional energy resources (e.g., coal, oil, and gas) because of their limited, exhausting supplies and environmental impact.

While energy from fossil fuels remains non-sustainable, solar energy is renewable, and it is generally considered the most promising way to solve the global energy crisis.

The conversion of sunlight energy to electrical energy is achieved in photovoltaic devices containing suitable semiconductors; first- and second-generation technologies use inorganic semiconductors such as crystalline silicon, thin layers of cadmium telluride, copper indium diselenide, and copper indium gallium selenide [1].

**Figure 1:**
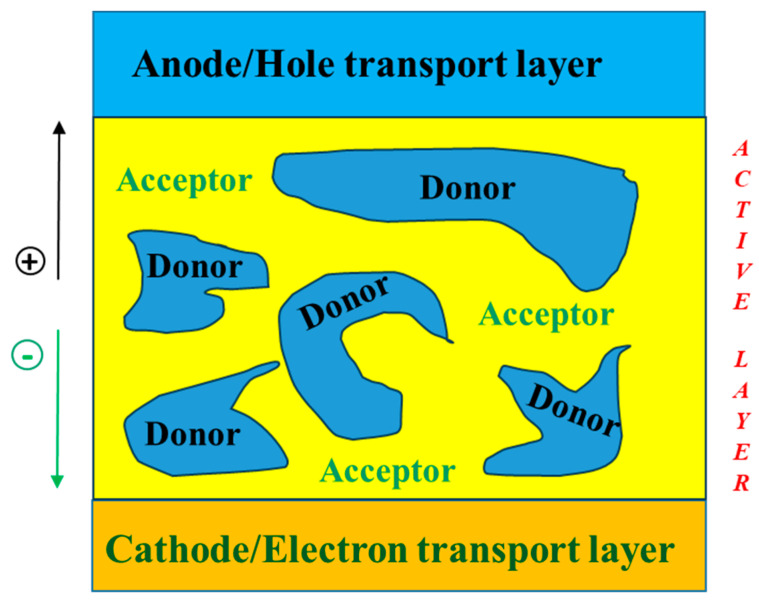
Bulk heterojunction organic solar cell scheme, adapted from [1].

In order to provide electricity at a lower cost, new photovoltaic systems have been developed albeit not yet commercialized at large scale. Among such emerging third-generation technologies, organic solar cells (OSC) based on polymer semiconductors, defined as polymer solar cells (PSC), are receiving more and more attention since the discovery of bulk heterojunction (BHJ) solar cells (Figure 1) in the early 1990s [2]. 

**Scheme 1:**
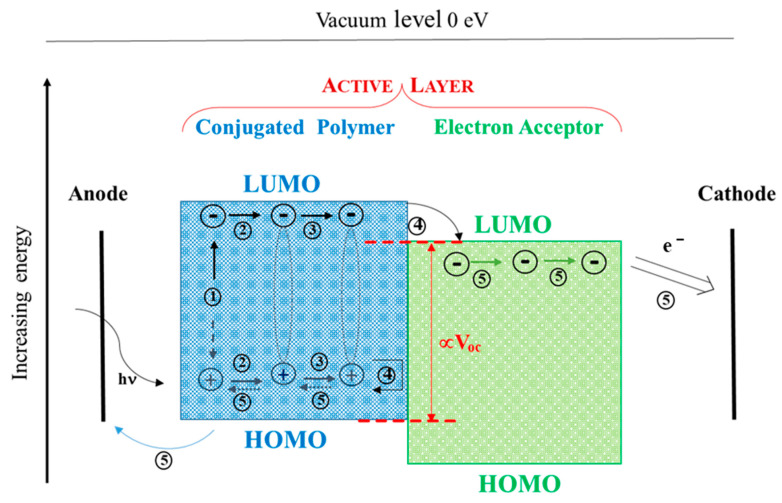
Electric charge generation mechanism and processes in BHJ organic cells: (1) light-generated exciton formation; (2) light-generated exciton diffusion; (3) charge-transfer exciton formation; (4) charge-transfer exciton dissociation/carrier formation; (5) carrier transportation and collection. Adapted with permission from Y. Bai, L.W. Xue, H.Q. Wang, Z.G. Zhang, Research Advances on Benzotriazole-based Organic Photovoltaic Materials. *Acta Chimi Sin* 79 (2021) 820–852. ©2021 Shanghai Institute of Organic Chemistry, Chinese Academy of Sciences [3].

In such a system, the active layer is obtained by blending a conjugated polymer serving as a donor (D) with an electron acceptor (A) to form bicontinuous interpenetrating networks at the nanoscale in order to favor separation into free carriers of the tightly bounded electron–hole pairs at the D/A interface, namely, excitons formed upon light excitation, and to allow migration of the charges to the respective electrodes (Scheme 1).

Photons passing through the transparent anode are absorbed by the donor molecules and, depending on their energy and the bandgap of the polymer, electrons will be excited from the highest occupied molecular orbital (HOMO) to the lowest unoccupied molecular orbital (LUMO), leaving a positive charge on the HOMO. Once the excitons reach the interface between donor and acceptor, it is thermodynamically more favorable for the electrons to be situated in the LUMO of the electron acceptor material lying below the LUMO of the donor and for the holes to remain in the HOMO of the electron donor material.

Such intermolecular charge transfer (CT) states should evolve into the separate charge states so that free electrons and holes are obtained and transported to the cathode and to the anode, respectively, minimizing charge recombination in the organic cells which would inhibit the total charge flux [4]; the charge carriers are taken to the respective electrodes with the aid of the internal electric field resulting from the difference in the working function of the electrodes. Photocurrent generation is therefore a stepwise process, where all the principal stages need to be optimized to produce photocurrent efficiently [5].

**Scheme 2:**
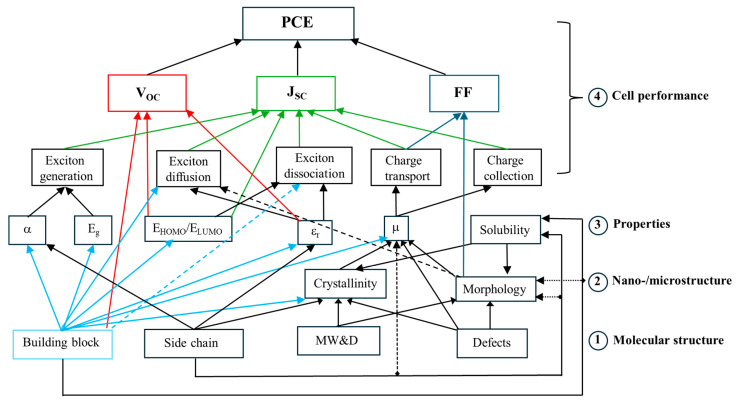
Scheme of the structure–property–cell performance relationships of a polymer donor for OSCs, where α is the absorption coefficient, ε_r_ is the dielectric constant (or relative permittivity), μ is the mobility, and MW and D are the molecular weight of the polymer donor and its distribution, respectively. Defects may include terminal groups, homo-coupled units in a copolymer, random arrangements of comonomers in a copolymer, regio-irregular units, branching, lightly cross-linked units, oligomers, etc. Some or all of these relationships may apply to small-molecule donors as well as polymer and small-molecule acceptors. The red lines evidence the connection with Voc, the green ones with J_SC_ and the blue ones with FF; the lines surrounding the building block and departing from it are in light blue to evidence better its several relationships with parameters different from Voc, J_SC_ and FF. Adapted from ref. [7] with permission from the Royal Society of Chemistry.

The performance of an OSC is therefore assessed in terms of power conversion efficiency (PCE or η, in percentage) which is directly proportional to short-circuit current density (J_SC_), open-circuit voltage (V_OC_), and fill factor (FF) as reported in the following equation:

PCE = (V_OC_ × J_SC_ × FF)/(I_P_ × M)

where I_P_ is the power density of the incident light irradiation and M is spectral mismatch factor [6]. Besides interface engineering for efficient charge collection, such parameters correlated to the photovoltaic processes are in particular influenced by material properties; considering that an OSC works through the close collaboration of matching donors and acceptors, the cell performance, properties, and the molecular and nano-/microstructures of polymer donor materials, which are ultimately governed by the molecular structure of the polymer donor, are connected by complex hierarchical relationships as illustrated in Scheme 2.

In order to find a system in which to tune so many strictly correlated variables, the scientific community began to focus their research on the development of donor–acceptor, D-A, conjugated polymers, regarded as the second sub-group of third-generation semiconducting polymers [8], which, having both electron-rich (pushing) units and electron-deficient (pulling) units, may allow the following: (1) a broad absorption in visible and near-infrared regions to harvest sun light efficiently to increase J_SC_, (2) suitable alignment of HOMOs and LUMOs for efficient charge separation and higher V_OC_, (3) good charge mobility to overcome recombination process and facilitate the charge transport efficiency for high FF and J_SC_, (4) processability in the fabrication of OSCs and suitable morphology, and (5) nanoscale phase separation for effective charge separation and extraction [9].

In particular, the donor–acceptor approach, first demonstrated by Havinga et al. [10], gained popularity as a means to narrow the bandgap of conjugated copolymers since the push–pull function among donor–acceptor units could promote charge transfer and create electronic delocalization [11].

Utilizing an electron-rich donor with a high-lying HOMO level in combination with an electron-deficient acceptor with a low-lying LUMO level, copolymerization of donor and acceptor monomers, which usually occurs via classical coupling reactions, such as Suzuki–Miyaura and Migita–Stille, determines the hybridization of their orbitals resulting in a reduced bandgap through the simultaneous raising of the HOMO and lowering of the LUMO and shifting optical absorbance to a lower energy compared to constituent donor and acceptor homopolymers [12].

**Figure 2:**
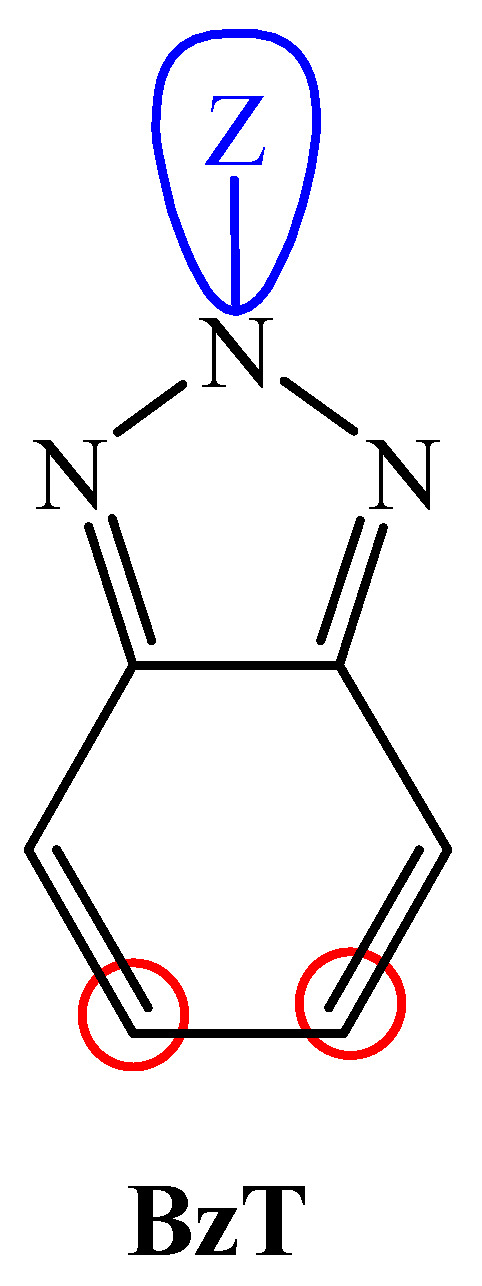
Molecular structure of benzotriazole in which the Z substituent and 5- and 6-position are highlighted.

Among the various acceptors for constructing the *p*-type semiconductor benzotriazole, BzT (Figure 2), a nitrogen-containing heterocyclic benzazole derivative is a moderately electron-deficient unit due to the diimine structure which offers the following chemically functionalizable sites [13,14]:

(1) The central nitrogen position can be functionalized with a Z group, mostly an alkyl chain, to endow solution processability, which, separate from the conjugated backbone, reduces the steric hindrance, thereby enhancing the effective intrachain π-conjugation and interchain packing; (2) The 5- and 6-positions on the benzotriazole unit can be modified chemically by introducing other substituents to modulate the frontier orbital levels and oxidational stability of the resulting molecules. 

On the other hand, regarding the *n*-type organic semiconductor of the active layer, fullerene derivatives have been widely used and investigated as acceptors because of their strong electron-accepting ability, high electron mobility, and ability to form proper-sized BHJ domains in PSC devices [15]. In order to overcome their weak absorption in the visible spectral region, limited energy level tunability, and the inadequate long-term stability of the devices caused by the susceptibility to dimerization and gradual aggregation, thanks to the invention of the A–D–A-structured small-molecule non-fullerene acceptor ITIC [16], in the last decade, non-fullerene acceptors (NFAs), consisting of organic small molecules or polymers, have been developed and successfully employed in OSCs with effective improvement to PCE [17,18] which can be achieved by following such important criteria: (1) complementary absorptions between donor and acceptor components to enhance light harvesting; (2) matching energy levels to ensure efficient charge separation and to minimize voltage loss (V_loss_, defined as V_loss_ = E_gap_/q − Voc, where E_gap_ is the lowest optical bandgap among the donor and acceptor components); (3) balanced charge mobilities to avoid charge accumulation at device interfaces; and (4) favorable morphology including high crystallinity, face-on orientation, and small domain size [19].

In particular, among the NFAs, BzT-based polymers have been very recently prepared and successfully tested as acceptors in the OSC active layers.

Benzatriazole is therefore an important building block both for donor and acceptor polymers; this review is meant to provide an overview of the progress over the last ten years on the use of this simple moiety for the synthesis of both BzT-based donor D-A conjugated polymers combined with NFAs and BzT-based acceptor polymers, in particular referring to PCE values of corresponding devices constructed with such polymers in their BHJ active layer. The OSC containing an active layer composed of D and A species will be indicated as **D**:**A**-based devices, and the chemical structures of NFAs used with a BzT-based polymer and the donor polymers used with BzT-based polymer acceptors mentioned throughout these sections are depicted in the Supporting Information (Figures S1 and S2).

## 2. D-A Conjugated Polymers

**Figure 3:**
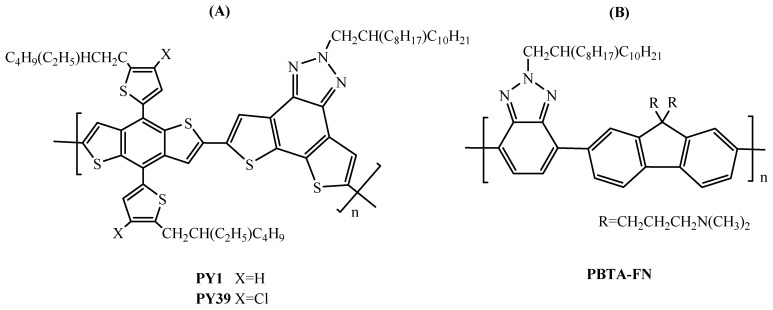
The structural formula of (**A**) **PY1**, **PY39** [20,21] and (**B**) **PBTA-FN** [22].

The literature is very rich with papers dealing with BzT-based D-A conjugated polymers, but to the best of my knowledge in NFAs-OSC, only three polymers (Figure 3) are precisely characterized by the formula D-A containing a benzotriazole-based electron-deficient unit directly linked to a donor, specifically, two benzo [1,2-b:4,5-b′]dithiophene derivatives (polymers **PY1** and **PY39)** [20,21] or a fluorene derivative (polymer **PBTA-FN**) [22].

**Scheme 3:**
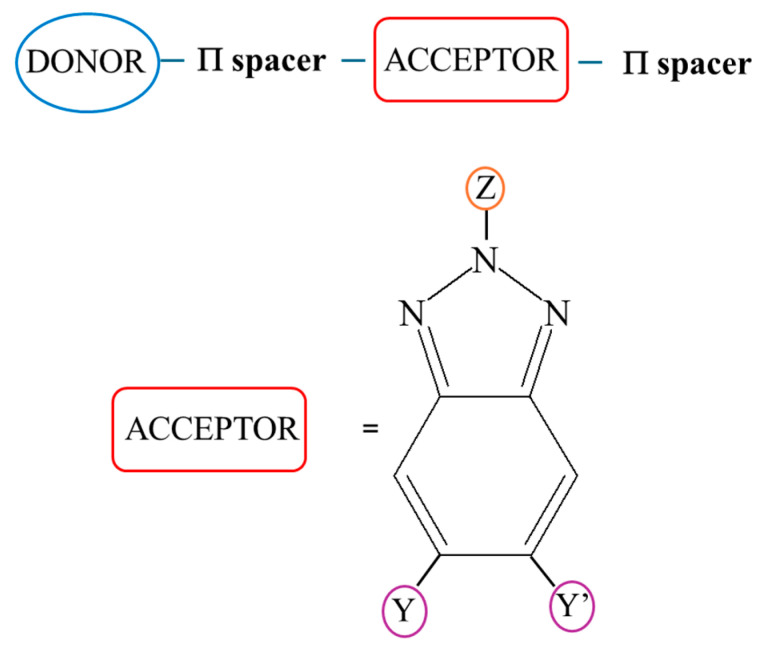
Scheme of BzT-based polymers having a D-π spacer-A-π spacer molecular formula.

It is common to indicate conjugated polymers generally as D-A species, but to be clear and precise, most of the polymers are characterized more properly by a formula such as D-π spacer-A-π spacer, as in Scheme 3; the D unit can be a single or a group of molecules, the π spacer is a bridging unit, specifically furan, thiophene, selenophene, and thieno[3,2-b]thiophene, and A is a BzT derivative, which contains different organic or siloxane groups Z on the azole nitrogen and Y,Y′ substituents consisting of H, Cl, F, CN, OR, or forming a ring condensed with BzT.

Considering the π-spacer, thiophene is one of the most used molecules as a π-bridge, since it has high charge-transport ability and extends the conjugation length of the polymer, and it is definitely the most used in BzT-based conjugated polymers. The introduction of thiophene on the polymer backbone decreases steric hindrance that may occur between D-A units and may favor planarity. Moreover, it determines the widening of the absorption in the direction of near-infrared region (NIR) wavelengths in addition to increasing the absorption coefficient due to strong intramolecular charge transfer (ICT) [23].

Considering the acceptor, it is possible to identify two main groups of polymers, one based on 5,6-difluorobenzotriazole (F-BzT) and one on the π-extended BzT unit consisting of the BzT unit condensed with various rings.

Therefore, conjugated polymers will be illustrated in two sections in which the polymers will be listed based upon the type of bridge and donor moiety.

### 2.1. F-BzT-Based Conjugated Polymers with Thiophene Bridge

**Figure 4:**
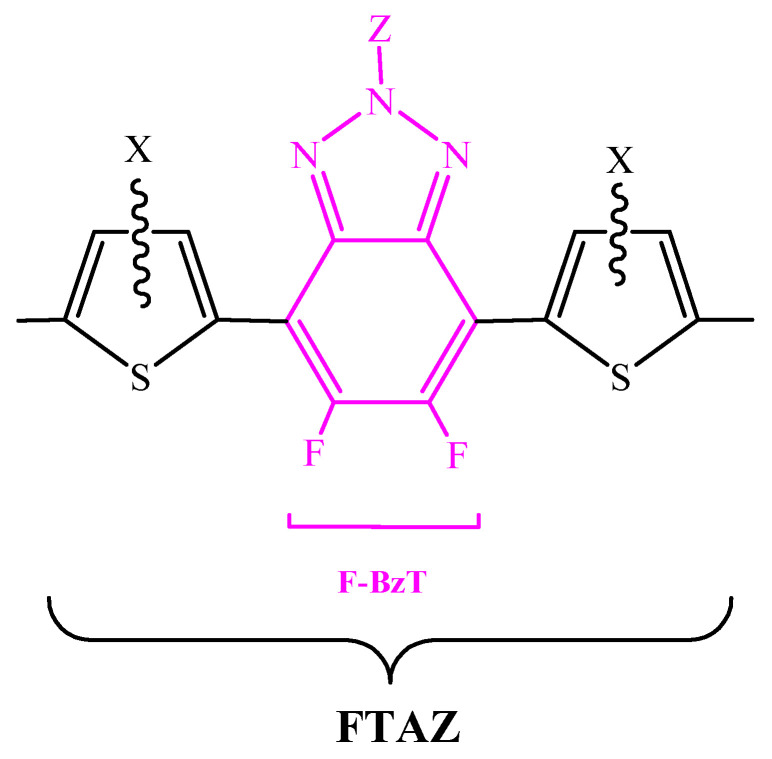
Structural formula of FTAZ where the F-BzT unit is highlighted in violet.

The 5,6-difluorobenzotriazole unit provides a lot of advantages for the constructions of conjugated polymers in comparison to simple BzT-based polymers [24,25,26,27,28,29,30,31,32], which may be attributed to its small van der Waals radius of 1.35 Å and high electronegativity, effectively modifying the energy levels and enhancing optical absorption without a negative influence on molecular packing. This unit with two flanking thiophenes, called FTAZ, is the most largely used BzT-based acceptor core (Figure 4).

#### 2.1.1. FTAZ-Conjugated Polymers with BDT as Donor Moiety

**Figure 5:**
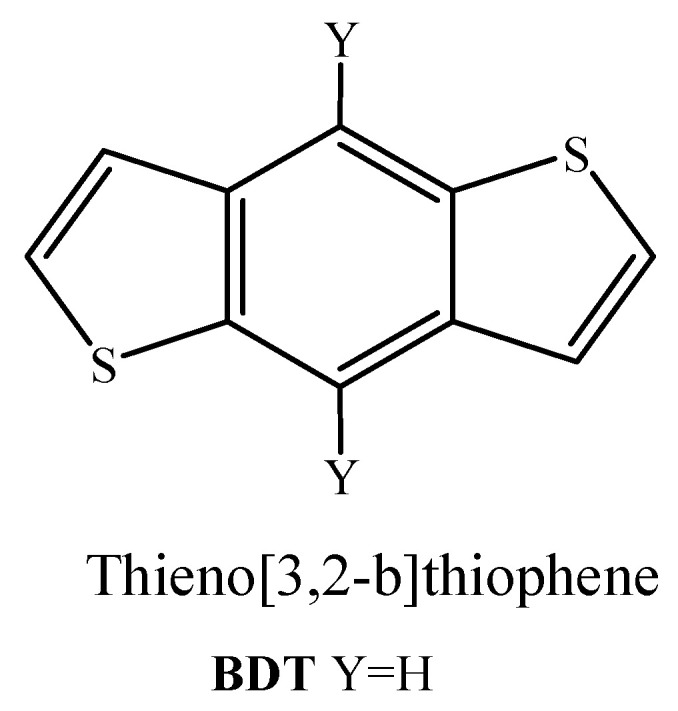
Structural formula of **BDT**.

Thieno[3,2-b]thiophene, BDT Figure 5, which is usually connected to the thiophene bridge along the polymer backbone via one of the condensed thiophenes except for just one case where it is linked via the X group, is the most used donor molecule in BzT-based polymers: actually, its rigid and planar structure provides great potential for tuning the energy levels, bandgaps, and charge carrier mobility with the desired chemical structure modifications via side-chain engineering, i.e., the convenient choice of the X group [26].

**Figure 6:**
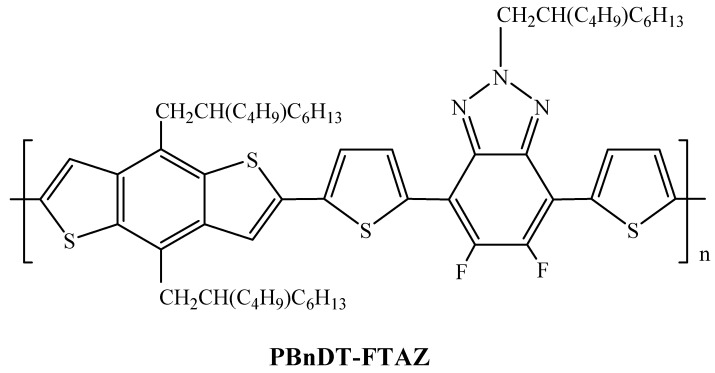
Structural formula of **PBnDT-FTAZ** [33].

The first polymer containing an alkyl derivative of BDT and FTAZ, **PBnDT-FTAZ,** Figure 6, was reported by Price et al. [33] and blended with the fullerene derivative [6,6]-phenyl C61-butyric acid methyl ester (PC61BM) as acceptor, obtaining a OSC with a PCE higher than 7%, quite relevant for that time for BzT-based polymers.

**Figure 7:**
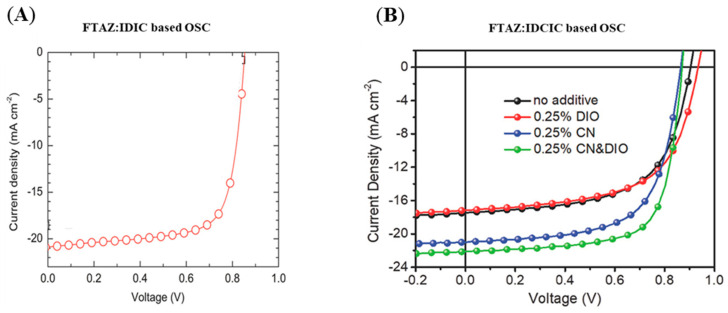
The J-V curves recorded under AM1.5G (100 mWcm^−2^) illumination of (**A**) **FTAZ:IDIC**-based device, [34] © 2018 WILEY-VCH Verlag GmbH & Co. KGaA, Weinheim; (**B**) **FTAZ:IDCIC**-based device, [35] © 2018 WILEY-VCH Verlag GmbH & Co. KGaA, Weinheim.

However, when the polymer was blended with NFAs, the PCE of the OSC containing such active layers increased except with SF-PDI2 [25] (Table 1), reaching the maximum value of 13.03% without additive and IDIC [34] or 13.58% with 1,8-diiodooctane (DIO) and chloronaphthalene (CN) with IDCIC [35]; the corresponding J-V curves are reported in Figure 7.

**Table 1:** PCE values of **D**:**NFA** OSCs under simulated AM1.5G (100 mWcm^−2^) illumination.

Polymer Donor	Non-Fullerene Acceptor	PCE_max_^a^ (%)	PCE_avg_^b^ (%)	References
**PBnDT-FTAZ**	*SF-PDI2*	2.3		[25]
**FTAZ ^c^**	*IDIC*	13.03		[34]
**FTAZ ^c^**	*IDCIC*	9.64 (13.58) ^d^	9.09 ± 0.34 (13.10 ± 0.26) ^d^	[35]
**FTAZ ^c^**	*ITIC 1*	8.54	8.32 ± 0.19	[36]
**FTAZ ^c^**	*ITIC 2*	11.0	10.6 ± 0.2	[36]
**FTAZ ^c^**	*ITIC-Th*	8.88	8.67 ± 0.15	[37]
**FTAZ ^c^**	*ITIC-Th 1*	12.1	11.9 ± 0.1	[37]
**FTAZ ^c^**	*INIC*	7.7	7.5	[38]
**FTAZ ^c^**	*INIC1*	10.1	9.9	[38]
**FTAZ ^c^**	*INIC2*	10.8	10.6	[38]
**FTAZ ^c^**	*INIC3*	11.5	11.2	[38]
**FTAZ ^c^**	*IHIC 2*	7.45	7.30 ± 0.18	[39]
**FTAZ ^c^**	*IOIC 2*	11.2 (12.1) ^e^	11.1 ± 0.1 (12.1± 0.2) ^e^	[39]
**FTAZ ^c^**	*IDIC*	12.5	12.1 ± 0.4 ^e^	[40]
**FTAZ ^c^**	*IT-M*	11.89 (12.22) ^f^		[41]
**PBnDT-FTAZ**	*IT-M*	12.0 ^g^	11.7 ± 0.3	[42]
**OTAZ ^h^**	*IT-M*	4.1	3.8 ± 0.2	[42]
**F OTAZ ^i^**	*IT-M*	5.7	5.2 ± 0.4	[42]
**4′-FT-FTAZ ^j^**	*ITIC-Th1*	10.3		[28]

^a^ PCE maximum value, reported for as-cast film from halogenated solvents unless otherwise specified. ^b^ PCE average values and standard deviations when available. ^c^ The authors have shortened the name **PBnDT-FTAZ** in just **FTAZ**. ^d^ Film processed with DIO and CN as additives. ^e^ Film processed with DIO as additive. ^f^ Film processed with 2,3,5,6-tetrafluoro-7,7,8,8-tetracyanoquinodimethane (F4-TCNQ) as additive. ^g^ Film processed with toluene. ^h^ Structural formula reported in Figure S3. ^i^ Structural formula reported in Figure S4. ^j^ Structural formula reported in Figure S5.

It was also possible to process a device with PCE over 11% using toluene instead of halogenated solvent [42], which holds great promise for the development of low-cost, low-toxicity, and high-efficiency OSCs as well as showing the successful combination of this wide-band-gap (WBG) polymer (E_g_ ca 2 eV) with NFAs with respect to fullerene acceptors.

In Table 1, PCEs of devices with slightly different polymers [**4′-FT-FTAZ** has fluorine in the β-position in the thiophene bridge while **OTAZ,** instead of fluorine in the 5- and 6-position, has two oligo-(ethylene-oxide) chains] [42] are reported in order to provide a full overview on the 2-butyloctyl-functionalized BDT donor derivative.

**Figure 8:**
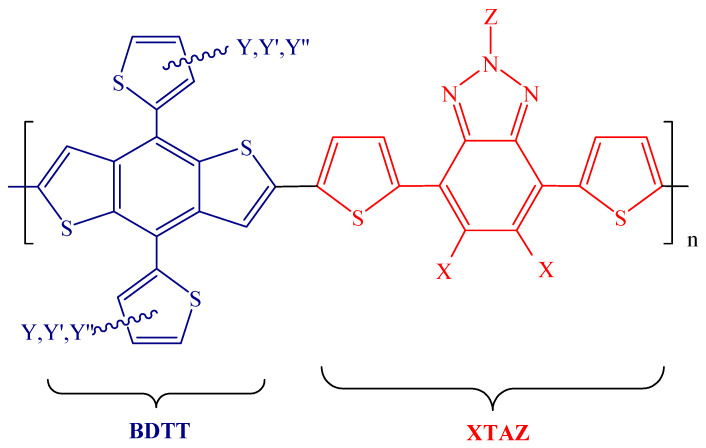
Structural formula of BDTT-alt-XTAZ polymers.

The use of NFAs was definitely a breakthrough in the development of OSCs, and in particular WBG (E_g_ 1.9–2.0 eV) polymers with a D–A molecular skeleton of thienyl benzodithiophene-alt-4,7-bis(thiophen-2-yl)benzotriazole (BDTT-alt-XTAZ), Figure 8, have become widely used and successful material systems due to their strong optical absorption in the wavelength range of 400–650 nm with a high absorption coefficient of ∼10^5^ cm^−1^ in film, ordered molecular packing, high hole mobility of ∼10^−3^ cm^2^ V^−1^ s^−1^, and favorable morphological insensitivity [43].

**Figure 9:**
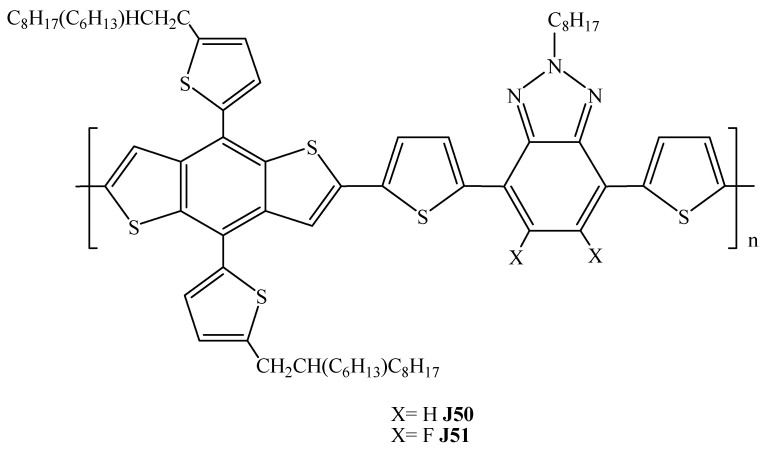
Structural formula of **J50** and **J51** polymers [44].

The first polymers **J50** and **J51**, Figure 9, were synthesized by Min et al. [44] who observed their two-dimensional character and the redshift in their absorption spectrum with respect to the linear copolymer analogues with alkoxy side-chains or alkyl side-chains on the BDT unit.

**Figure 10:**
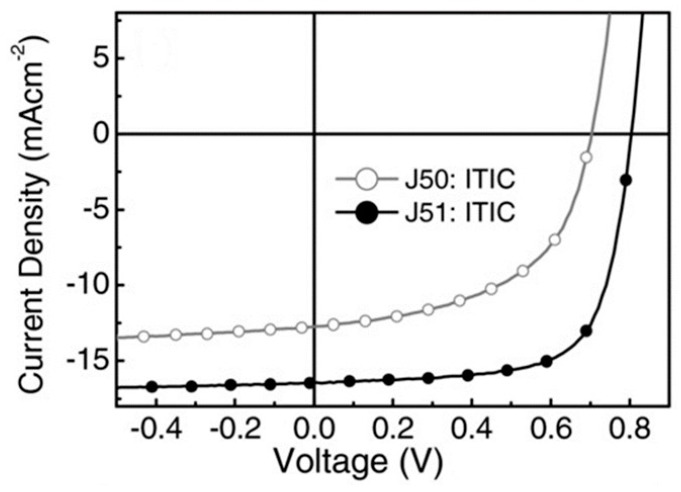
The J-V curves of a **J51:ITIC**-based device recorded under AM1.5G (100 mWcm^−2^) illumination [45] © 2016 WILEY-VCH Verlag GmbH & Co. KGaA, Weinheim.

The combination of **J51** with the polymer acceptor N2200 allowed researchers to produce the first all-polymer cell containing a BzT-based polymer donor, with PCE of 8.28% [24]. Better efficiency was achieved with ITIC (PCE 9.26%), having a stronger absorbance than N2200 in the longer-wavelength region [45]: the J-V curves are reported in Figure 10.

**Figure 11:**
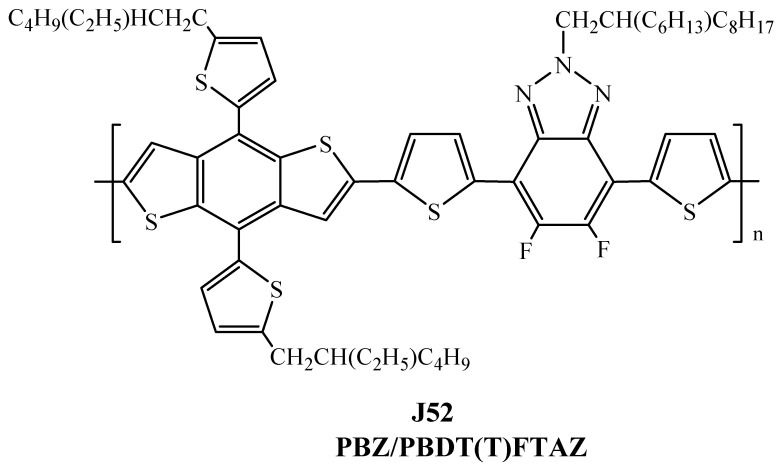
Structural formula of the **J52/PBZ/PBDT(T)FTAZ** polymer with the different names [50,51,52].

Except for a few polymers in which the alkyl group bound to BDTT in the α-position was substituted by an ethynyl derivative (2-triisopropylsilylethynyl) [46] or linear hexyl/octyl chain [47,48,49], research was devoted to the study of polymers with the 2-ethylhexyl group, **J52** [50] Figure 11, also indicated as **PBZ** [51] or **PBDT(T)FTAZ** [52].

**Figure 12:**
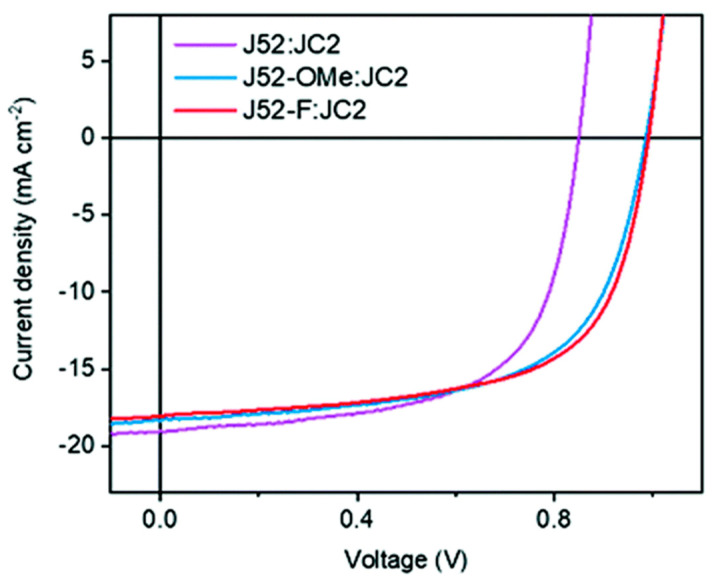
The J-V curve of a **J52:JC2**-based device recorded (violet line) under AM1.5G (100 mWcm^−2^) illumination reproduced from [56] with permission from the Royal Society of Chemistry.

This was tested with various acceptors [50,52,53,54,55,56,57,58,59] (Table 2), the best performance being obtained when **J52** was paired to a JC2 acceptor (PCE 10.27%) (the J-V curve is shown in Figure 12) [56].

**Scheme 4:**
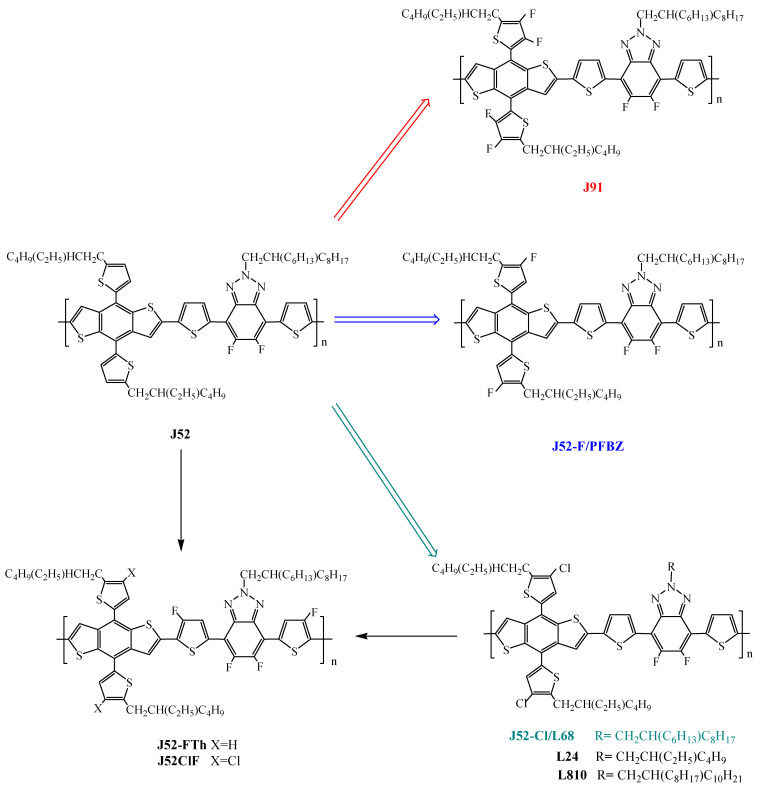
Scheme of **J52**-halogenated derivatives [21,29,43,51,53,54,55,56,59,64,65,66,67,68,69].

**J52** was also copolymerized with other polymers to obtain random terpolymers (Figure S6) [52,60], or slightly changed by introducing a siloxane group on the azole nitrogen to provide **J55** (Figure S7A) [61], but it mostly underwent further functionalization of the thienyl group with the introduction of fluorine or chlorine (Scheme 4).

Fluorination is a well-known method to improve photovoltaic performance: first, from a synthetic point of view, fluorine does not react with aromatic boronic or stannyl monomers in the palladium-catalyzed carbon–carbon coupling reactions, which are the most applied methods in the synthesis of polymers for OSCs [62]. Fluorine is more electronegative than hydrogen (3.98) and has a similar size to hydrogen. The small size and high electron affinity of the fluorine atom can effectively amend the energy levels by lowering both HOMO and LUMO energy levels with benefits for V_OC_ [63]. With a fluorine substitution in the donor unit, the resulting polymer will possess a minimized steric effect due to the similar size of fluorine and hydrogen. Besides the significant effect on the HOMO energy levels in these fluorine-substituted polymers, the intermolecular noncovalent interactions such as F^…^H(O) or F^…^F can also affect the molecular stacking of polymer chains as a result of more ordered aggregation in the nanoscale phase-separated bulk-heterojunction film; they potentially also improve charge carrier mobility, what is beneficial to J_SC_.

As expected, the monofluorinated **J52-F** (Scheme 4) also indicated as **PFBZ** exhibited lower HOMO/LUMO levels, stronger p–p interaction, higher extinction coefficient, and smaller stacking spacing than **J52** [51] which resulted in the better performance of **PFBZ**:**ITIC**-based OSCs, yielding a higher PCE of 10.4% (Table 3). Moreover, the PCE values of the **PFBZ**:**ITIC**-based devices are insensitive to the variation of active layer thickness and the **PFBZ**:**ITIC**-based devices exhibit high tolerance to the thermal annealing.

**Figure 13:**
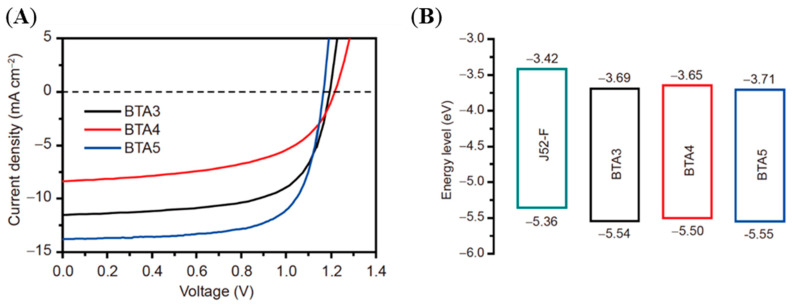
*(***A**) The J–V curve of **J52-F:BTA3**-, **J52-F:BTA4**-, and **J52-F:BTA5**-based devices recorded under AM1.5G (100 mWcm^−2^) illumination. (**B**) Energy level diagram of **J52-F** and acceptors BTA3, BTA4, and BTA5 adapted from ref. [64] with permission from Springer Nature© Science China Press and Springer-Verlag GmbH Germany, part of Springer Nature 2020.

**J52-F** was tested also with other NFAs (Table 3): BTA5 is the acceptor more suitable to determine a higher PCE (the J-V curves are reported in Figure 13A) and in particular high V_OC_ (1.17 V), which is, however, the lowest V_OC_ among the three devices (V_OC_ 1.19 V for **J52-F:BTA3**, V_OC_ 1.21 V for **J52-F:BTA4**) [64]. In particular, the highest V_OC_ value, 1.21 V, resulted from the raised lowest unoccupied molecular orbital energy level of BTA4 with respect to the other acceptors (Figure 13B).

**Figure 14:**
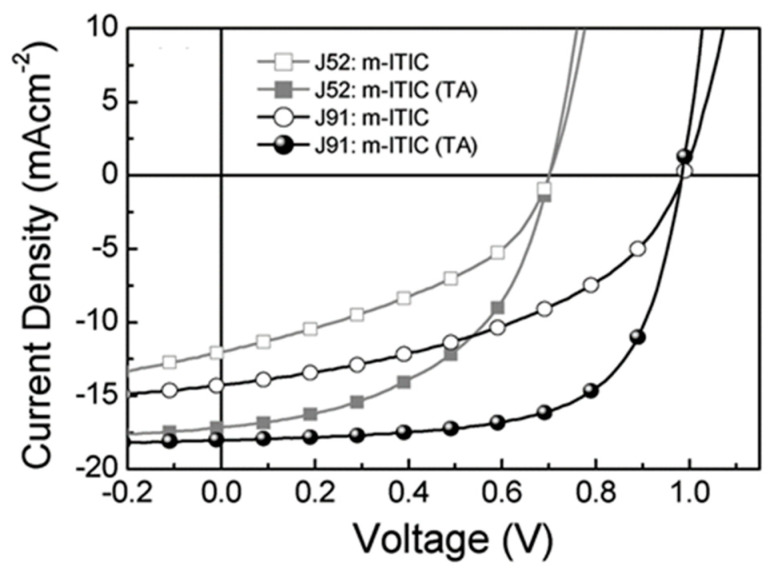
The J-V curves of **J91:m-ITIC**- and **J52:m-ITIC**-based devices recorded under AM1.5G (100 mWcm^−2^) illumination [53] © 2017 WILEY-VCH Verlag GmbH & Co. KGaA, Weinheim.

Also, double fluorination of the BDT thienyl side-chain group was successful in producing polymer **J91** which demonstrated enhanced absorption, low-lying highest occupied molecular orbital energy level, and higher hole mobility in comparison with its control polymer **J52**; the **J91**:**m-ITIC** based OSC performed better (PCE 11.63%, Voc 0.984 V, Jsc 18.03 mAcm^−2^ and FF 65.54%) than the analogous one with **J52** (PCE 5.98%, Voc 0.701 V, Jsc 17.16 mAcm^−2^, FF 49.73%) [53]; the J-V curves are reported in Figure 14.

It is interesting also to observe that, similar to the F atom, chlorine too shows strong electronegativity and demonstrates the large potential to adjust the molecular energy levels, crystallinity, and carrier mobility of the target polymers; in particular, due to the heavy atom effect and empty 3d orbitals of the chlorine atom, the HOMO energy level of the target polymer is lowered further than that of the fluorine substituted polymer [62]. Not secondarily, the synthetic costs are much lower and routes of chlorination more simple.

**Figure 15:**
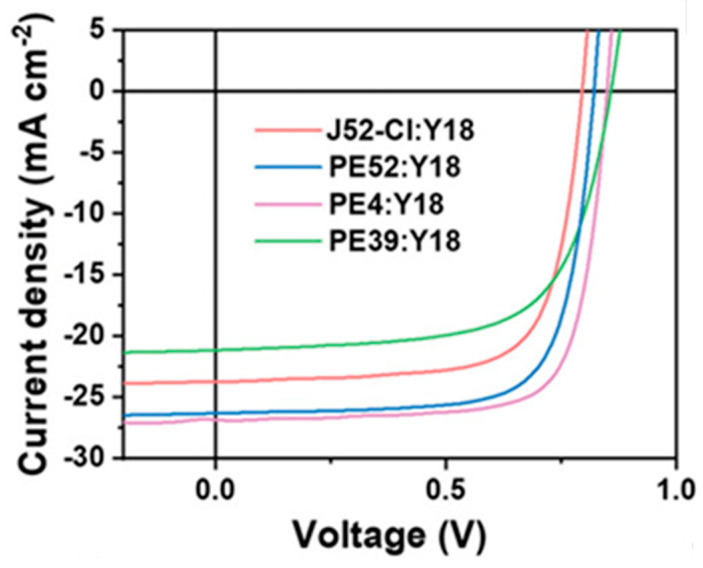
The J-V curve of the **J52-Cl:Y18**-based device (red line) recorded under AM1.5G (100 mWcm^−2^) illumination [21] ©2023 Wiley-VCH GmbH.

Therefore, **J52-Cl**, also indicated as **L68** [66] (Scheme 4), the analogous chlorine derivative of **J52-F**, was synthesized and tested with various acceptors (Table 3) [21,29,55,56,66,67], obtaining OSC devices with very promising PCEs (Table 3) ranging from 11.53% with ITIC [55] up to 13.64% with Y18 [21]; the J-V curve of the **J52-Cl:Y18**-based device is reported in Figure 15.

**Figure 16:**
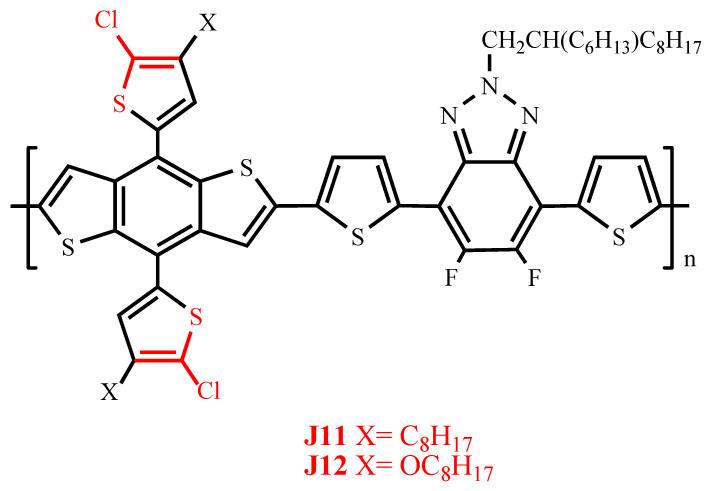
Structural formula of **J11** and **J12** [70,71].

Moreover, because of a feasible chlorination synthetic pathway for the thiophene group linked to BDTT, it was possible to prepare efficient chlorinated polymer donors with a low-lying HOMO energy level altering the position of the chlorine atom from the meta- to the ortho-position of the thiophene unit (Figure 16) [70,71].

**Figure 17:**
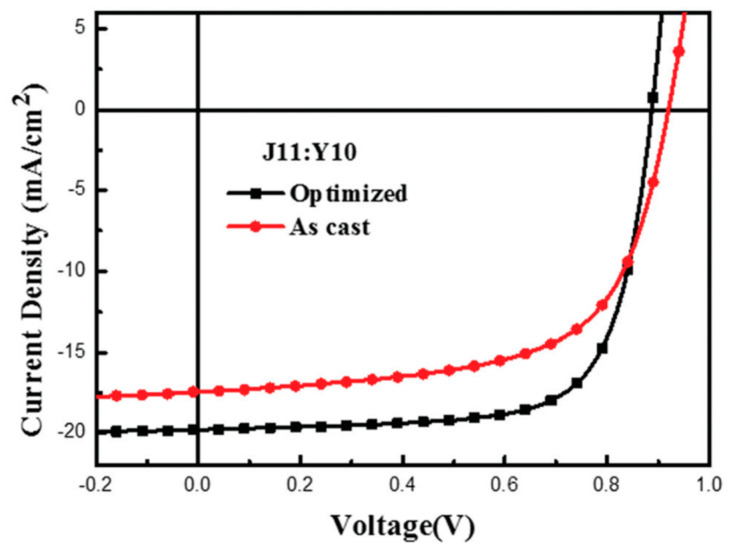
The J-V curve of **J91:Y10**-based device recorded under AM1.5G (100 mWcm^−2^) illumination reproduced from ref. [71] with permission from the PCCP Owner Societies.

In particular, the OSC containing **J11** blended with m-ITTC performed better than the one with **J12** and m-ITTC (Table 3), exhibiting a high PCE of 12.32% with a high FF of 73%, which even increased with Y10 (PCE 13.46%); the J-V curve of the **J11Cl:Y10**-based device is reported in Figure 17. This is thanks to the more redshifted spectrum of Y10, which is characterized by a narrow optical bandgap (E_g_^opt^) of 1.35 eV compared to the E_g_^opt^ of m-ITTC (1.61 eV), which is beneficial for obtaining a higher Jsc [71].

**Figure 18:**
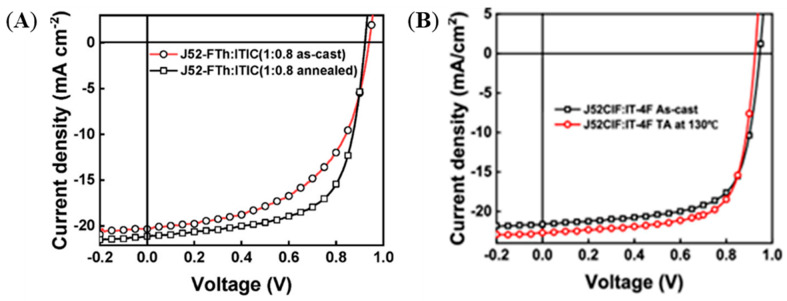
The J-V curves of (**A**) **J52-FTh:ITIC** reproduced with permission from [69] *Organic Electronics* 108 J.X. Liao, F.B. Weng, P.J. Zheng, G.B. Xu, L.X. Zeng, Z.G. Huang, T.J. Deng, Y.Q. Pang, S.Y. Wu, J.H. Chen, H.B. Zhao, Y.J. Xu, Enhanced efficiency of polymer solar cells via simple fluorination on the π-bridge of polymer donors, © 2022 Published by Elsevier B.V (2022) and (**B**) **J52ClF:IT-4F**-based devices recorded under AM1.5G (100 mWcm^−2^) illumination, [51] © 2017 WILEY-VCH Verlag GmbH & Co. KGaA, Weinheim.

Other side-chain engineering on **J52-Cl** regarded the R groups of the azole nitrogen to produce **L24** and **L810**, (Scheme 4, Table 3) [66], and very recently further molecular engineering of **J52** and **J52-Cl** occurred by replacing the thiophene π-bridge with 3-fluorothiophene in the main chain to obtain **J52-FTh** [69] and **J52ClF** [51] (Scheme 4), which notably improved the photovoltaic performance (Table 3): in particular, the PCE of the **J52ClF**:**IT-4F**-based OSC was boosted from 9.7 [50] to 14.59% [62] by realizing extensive and important noncovalent contacts such as F−H, F−S, and F−Cl. The J-V curves of **J52Fth:ITIC**- and **J52ClF:IT4F**-based devices are reported in Figure 18.

**Figure 19:**
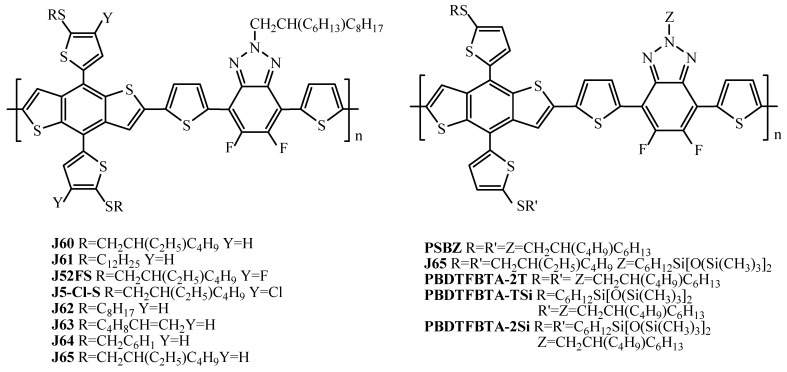
Structural formula of alkylthio-substituted BDTT-alt-FTAZ [50,54,57,61,72,73,74,75,76,77,78].

Besides alkyl chains, alkylthio groups were used as substituents of the BDTT unit to develop the donor polymers reported in Figure 19 (PCEs in Table 4) [50,54,61,72,73,74,75,76,77,78].

The incorporation of the alkylthio groups was inspired by sulfur’s special function of forming pπ(C)–dπ(S) orbital overlap between the conjugated side-chains and the alkylthio substitution, thus down-shifting the highest occupied molecular orbital (HOMO) levels and redshifting absorption.

It is interesting to observe that, differently from the polymers previously described, the research of such was particularly focused on evaluating the photovoltaic properties given the variations of the topology of the BDTT substituents, i.e., selecting linear or branched chains with different length which strongly affects the aggregation state and morphology and, therefore, PCE.

**Figure 20:**
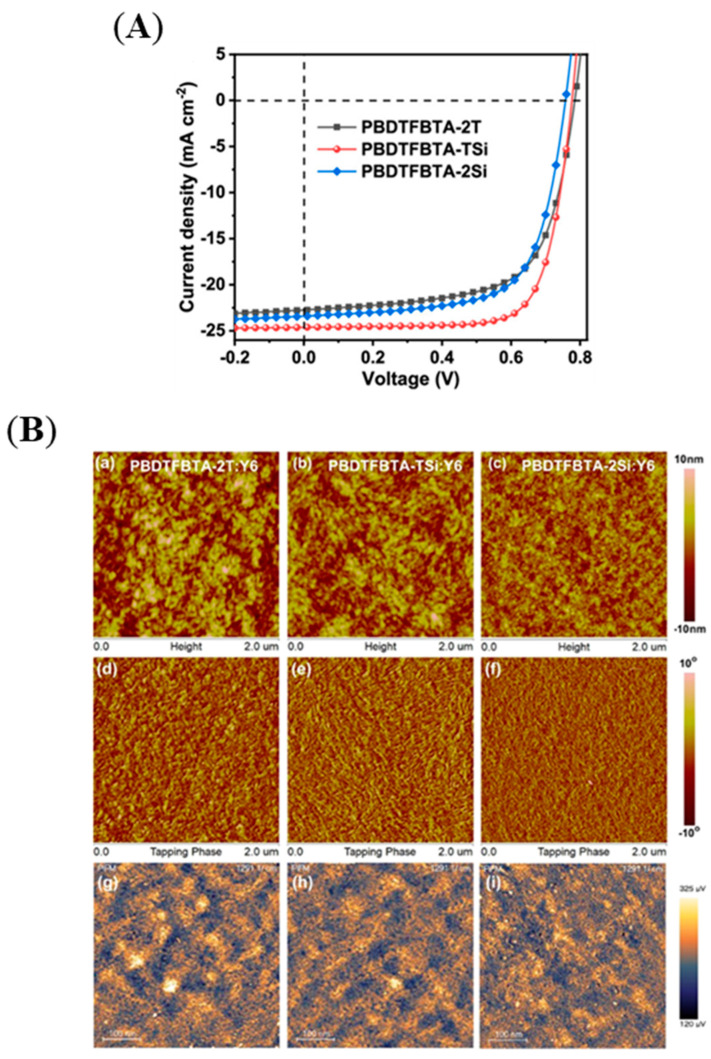
(**A**) The J-V curve of the **PBDTFBTA-TSi**:**Y6**-based device (red line) recorded under AM1.5G (100 mWcm^−2^) illumination in red reproduced from [78] with permission of American Chemical Society © 2020 American Chemical Society. (**B**) Atomic Force Microscopy (AFM) height images (**a**–**c**) and phase images (**d**–**f**) of the active blends. (**g**–**i**) PiFM images of the active blends adapted from [78] with permission of American Chemical Society © 2020 American Chemical Society.

The best performance (PCE 14.18%) was obtained by a device fabricated with the asymmetric siloxane-functionalized polymer **PBDTFBTA-TSi** blended with Y6 (the J-V curve of the **PBDTFBTA-TSi**:Y6-based device is shown in Figure 20A) [78]; the introduced siloxane functional groups showed less of an effect on the absorption and frontier orbital levels of the polymers but had a significant effect in improving the miscibility between the donor polymers and the non-fullerene acceptor, weakening the phase separation of the related blend films, which allowed for finely tuning the active blend morphology. As a consequence, **PBDTFTBA-TSi**:**Y6** blends had the most balanced crystallinity and miscibility with more interpenetrating microstructures (Figure 20B), generating the most appropriate phase separation for exciton dissociation and charge transport and resulting in a high PCE value.

Moreover, in order to construct a device with higher PCE, rather than ternary copolymerization [79], one facile solution is to use a ternary approach: two donor polymers having complementary absorption spectra are combined with an acceptor to construct a ternary solar cell [80,81]. In this way, PCE of 14.88% was achieved when **J61** was blended with **PffBTT2-DPPT2** (Figure S8) and Y6 [78,81].

**Figure 21:**
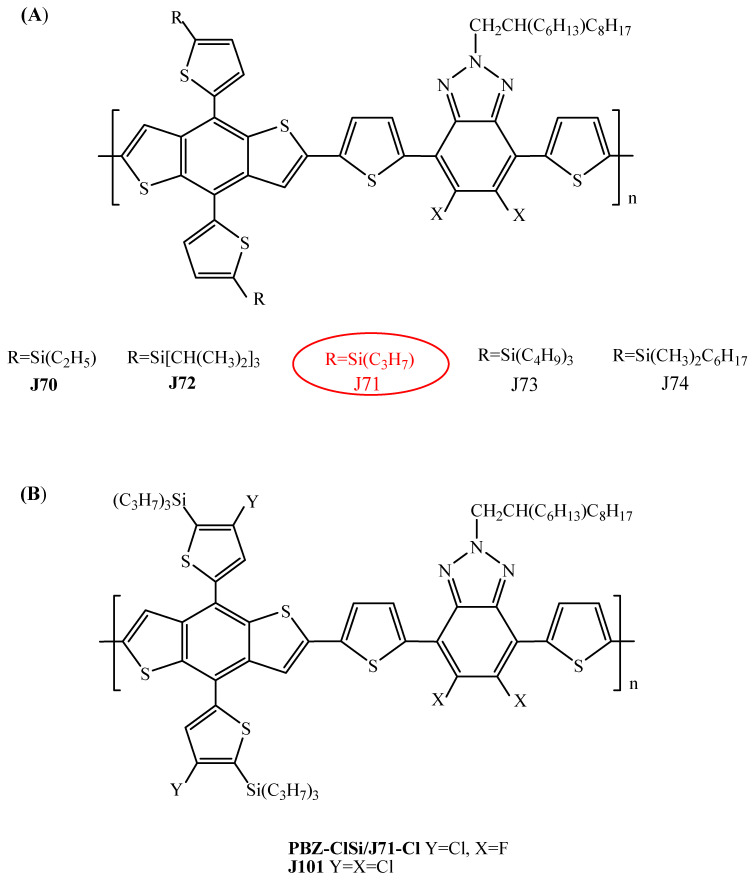
Structural formula of (**A**) alkylsilyl-substituted BDTT-alt-FTAZ, **J70-74** [30,82,83,84,85]; (**B**) halogenated alkylsilyl-substituted BDTT-alt-FTAZ, **PBZ-ClSi/J71-Cl** [30,43], **J101** [30].

**Figure 22:**
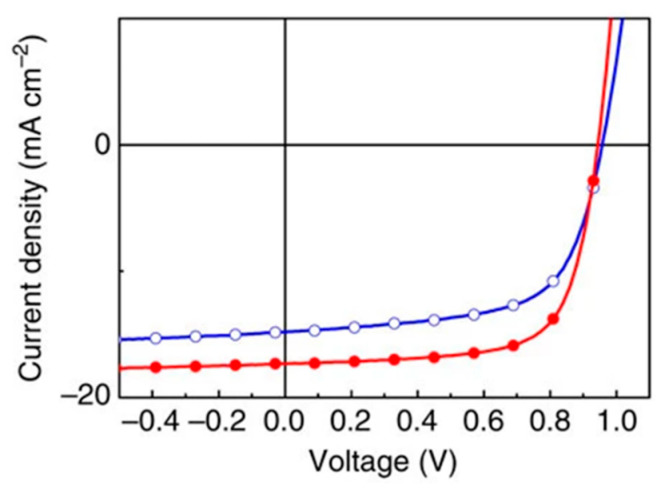
The J-V curve of **J71**:**ITIC**-based devices without (open circles) and with (filled circles) thermal annealing at 150 °C for 10 min recorded under AM1.5G (100 mWcm^−2^) illumination [82].

The last group of BDTT substituents are alkylsilyl groups: the alkylsilyl side-chain approach developed by Bin et al. [82] is simple and convenient for downshifting the HOMO energy level and strengthening the absorption due to the bond interaction of the low-lying s* orbital of the Si atom with the p* orbital of the aromatic units. Moreover, the Si atom has a significant effect on the crystallinity of the polymer. Bin et al. [82] synthesized polymer **J71** (Figure 21A) which, blended with ITIC, produced devices with PCE of 11.4% (the J-V curve of **J71**:**Y6**-based devices is shown in Figure 22).

Later on, different alkyl groups (branched or linear chains longer than n-propyl) were introduced to produce polymers **J70**, **J72**, **J73**, and **J74** (Figure 21A) (Table 5) [83], but most of the research was based on testing **J71** with various NFAs or also other deposition techniques [84,85,86,87].

It is noteworthy to underline that chlorination, analogously to **J52** derivatives, helped in improving PCE [43].

**Figure 23:**
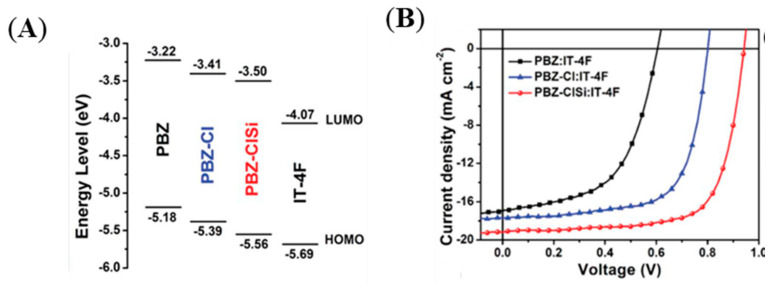
(**A**) The energy level diagram of **PBZ**, **PBZ-Cl**, **PBZ-ClSi**, and IT-4F. (**B**) The J-V curves of **PBZ**:**IT-4F**-, **PBZ-Cl**:**IT-4F**-, and **PBZ-ClSi**:**IT-4F**-based devices recorded under AM1.5G (100 mWcm^−2^) illumination adapted from ref. [43] with permission from the Royal Society of Chemistry.

In particular, **PBZ-ClSi** (**J71-Cl**) (Figure 21B), thanks to the presence of Cl and alkylsilyl substituents, showed reduced HOMO levels (Figure 23A), increased absorption coefficient, and improved charge mobility with respect to **J52** and **J52-Cl** [43].

As a result, the non-halogen-solvent-processed OSC based on **PBZ-ClSi**:**IT-4F** achieved a high PCE of 12.8% with a high V_OC_ of 0.93 V, J_SC_ of 19.2 mA cm^−2^, FF of 71.5%, and E_loss_ as low as 0.57 eV, while the OSCs based on **PBZ**:**IT-4F** and **PBZ-Cl**:**IT-4F** performed worse (PCEs of 6.4% and 9.7%, respectively); the corresponding J-V curves are reported in Figure 23B.

Good performance was also obtained for **J101** (Figure 21B, Table 5) where chlorine was introduced into the main chain of the **BTD-alt-XTAZ** polymer as well as in the side-chains [30].

Besides thienyl and analogous selenophene groups [88], alkoxide and aryl species were used to functionalize BDT to produce other donor polymers.

**Figure 24:**
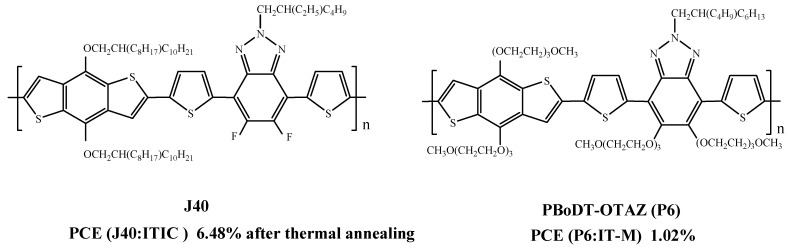
Structural formula of **J40** [89] and **P6** [90] and PCEs of corresponding devices.

The alkoxide derivatives **J40** and **P6** (Figure 24)-based OSCs did not show great performance even when the introduction of oligo(ethylene glycol) side-chains also on the benzo-group of BzT allowed the processing of the active layer film with 2-methyltetrahydrofuran (2-MeTHF), a renewable and green solvent [89,90].

To achieve better performance, it was necessary to introduce a two-dimensional (2D) side-chain which extends the π-conjugation and strengthens the intermolecular interactions, enhancing the light-harvesting and charge transport and lowering the HOMO energy levels.

**Figure 25:**
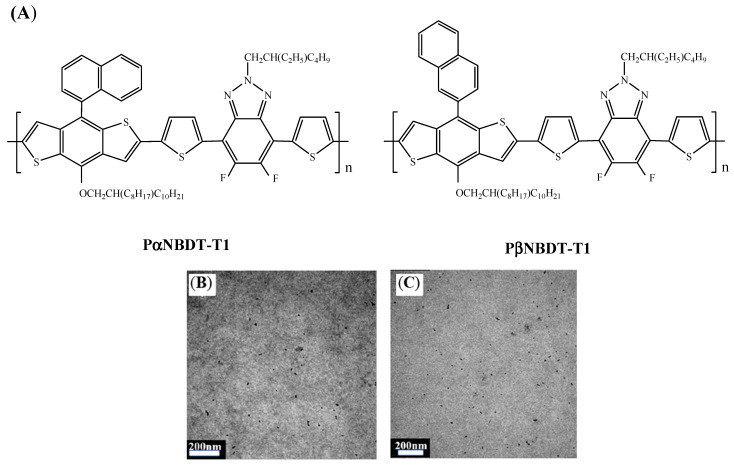
(**A**) Structural formula of **PαNBDT-T1** and **PβNBDT-T1** [91]; (**B**) TEM image of the active layer containing **PβNBDT-T1**:**ITIC** (1:1.5, *w*/*w*); (**C**) TEM image of the active layer containing **PαNBDT-T1**:**ITIC** (1:1.5, *w*/*w*) adapted from ref. [91] with permission of the Royal of Chemistry.

This strategy was followed by Liu et al. [91] who introduced 2-octyldodecylalkoxide, a one-dimensional side-chain, and a naphthalene group, a 2D side-chain, as well on BDT and prepared two polymers (Figure 25A): in particular, the OSC fabricated with the polymer containing the naphtalene linked in the α-position to BDT, **PαNBDT-T1**, blended with ITIC showed a PCE of 9.60%, higher than with the other isomer **PβNBDT-T1** (PCE 6.73%). This different performance is particularly ascribed to a better blending morphology with a more uniform phase separation as evident from transmission electron microscopy (TEM) images (Figure 25B,C) because of weaker intermolecular stacking due to a larger dihedral angle between the naphthalene rings (α form) and BDT.

**Figure 26:**
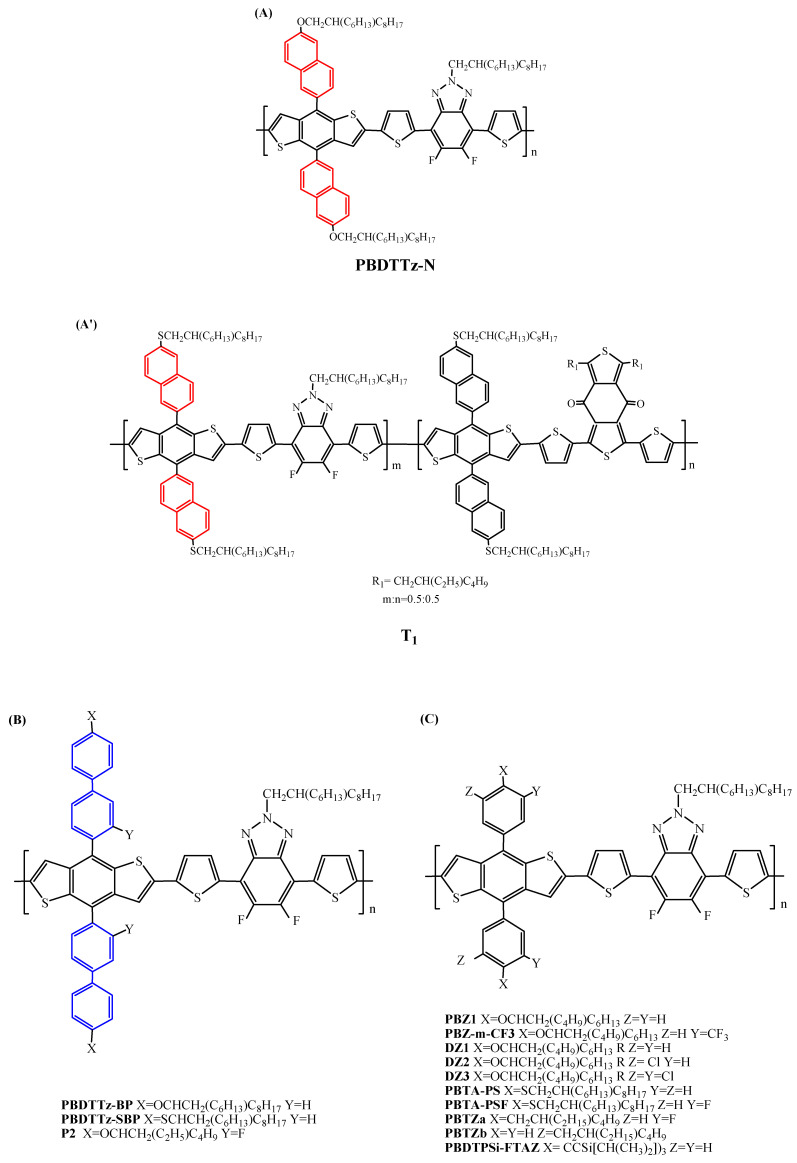
Structural formula of aryl-substituted BDT-alt-FTAZ [47,92,93,94,95,96,97,98,99,100]: (**A**) Structural formula of **PBDTTz-N**, (**A’**) Structural formula of **T1**, (**B**) Structural formula of **PBDTTz-BP, PBSTTz-SBP, P2**, (**C**) Structural formula of **PBZ1**, **PBZ-m-CF3**, **DZ1**, **DZ2**, **DZ3**, **PBTA-PS**, **PBTA-PSF**, **PBTZa**, **PBTzb, PBDTPSi-FTAZ**.

Two more polymers containing just naphthalene derivatives on the BDT skeleton (**PDTTz-N**, **T1**, Figure 26A,A’) were prepared and studied (Table 6) [92,93], but beyond naphthalene as 2D side-chain on BDT, the aryl substituent linked to BDT is usually a benzene ring functionalized with other phenyl, i.e., **PBDTTz-BP** [92], **PBDTTz-SBP** [47], and **P2** [94] (Figure 26B), alkoxide (**PBZ1** [95,96], **PBZ-m-CF3** [95], **DZ1, DZ2** and **DZ3** [97]) (Figure 26C), alkylthio (**PBTA-PS** and **PBTA-PSF** [98] Figure 26C), alkyl (**PBTZa** and **PBTZb** Figure 26B [99]) or trialkylsilylethynyl groups (**PBDTPSi-FTAZ** Figure 26C [100]).

Actually, the phenyl group is an alternative pathway to thiophene by extending the π-conjugated degree through the polymer which would be very helpful in delocalizing the electron cloud, adjusting the frontier energy level, molecular conformation, and charge transport [47]; PCE values are reported in Table 6.

**Figure 27:**
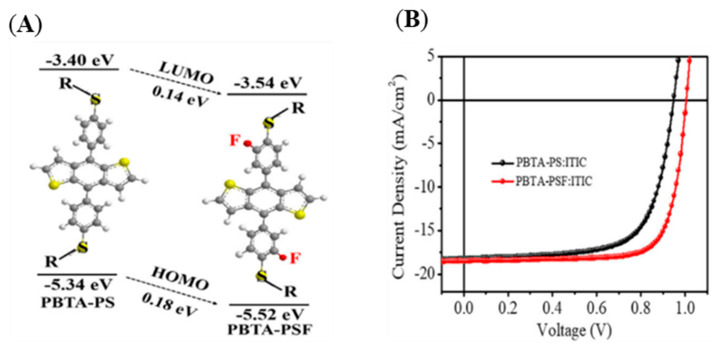
**(A**) The energy level diagrams of donors PBTA-PS and PBTA-PSF. (**B**) The J-V curves of **PBTA-PSF**:**ITIC** (red line)-based device recorded under AM1.5G (100 mWcm^−2^) illumination [98] © 2019 WILEY-VCH Verlag GmbH & Co. KGaA, Weinheim.

It is interesting to report that, by taking advantage of the weaker electron-donating nature of the phenyl-substituted BDT with respect to the thienyl-substituted BDT and thanks to the introduction of fluorine, the **PBTA-PSF**:**ITIC**-based device could reach a high PCE of 13.91% with a V_OC_ higher than 1 V (1.01 V), promoted by the low HOMO energy level (Figure 27A), a large J_SC_ of 18.51 mAcm^−2^, and an FF of 74.40% (the J-V curve of the **PBTA-PSF**:**ITIC**-based device is shown in Figure 27B) [98].

**Figure 28:**
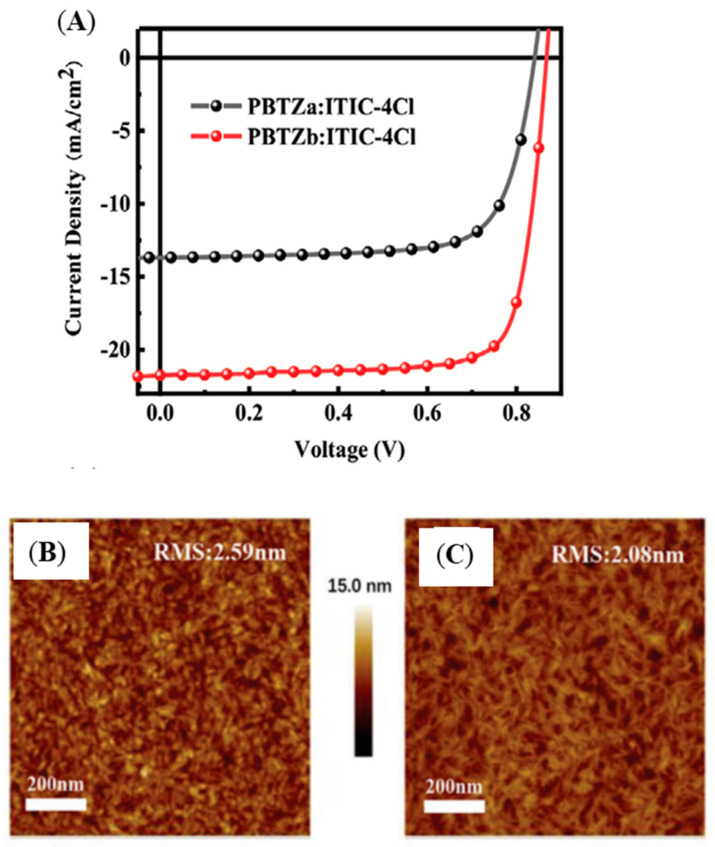
(**A**) The J-V curves of **PBTZb**:**ITIC-4Cl** (red line)-based devices recorded under AM1.5G (100 mWcm^−2^) illumination. (**B**) AFM height image (1 × 1 µm) of **PBTZa:ITIC-4Cl** blend. (**C**) AFM height image (1 × 1 µm) of **PBTZb:ITIC-4Cl** blend [99] © 2020 Wiley-VCH GmbH.

On the other hand, the introduction of an alkyl substituent on the phenyl group (in particular, 2-ethylhexyl to produce the two isomers **PBTZa** and **PBTZb** [99]) via a simple and low-cost chemical strategy (Grignard reagents rather than lithium-based reagents) allowed researchers to finely tune the polymer crystallinity and further optimize the miscibility between donor and acceptor. The **PBTZb**:**ITIC-4Cl**-based device performed better than that based on **PBTZa**:**ITIC-4Cl** with PCE of 14.53%, Jsc of 21.75 mAcm^−2^, and a high fill factor of 77% which could be attributed to a more balanced charge-carrier transport ability and better morphology (the J-V curve of the **PBTZb**:**ITIC-4Cl**-based device is shown in Figure 28A, AFM images in Figure 28B,C). The polymer crystalline domains are slightly damaged and passed by the acceptor as indicated by lower RMS roughness for **PBTZb** blend film (2.08 in comparison to 2.59 for **PBTZa** blend film, Figure 28B,C). The **PBTZb** blend exhibited an appropriate nanoscale phase separation, which then can facilitate charge separation and transport, beneficial to BHJ PSCs. On the contrary, due to the relatively larger phase separation, the coarse morphology of the **PBTZa** blend caused inferior overall photovoltaic performance.

By blending **PBz-1** with 20% **PTB7-Th** (Figure S9) and L8-BO, it was possible to achieve a PCE of 15.85% [96]; J_SC_ increased without affecting V_OC_ and FF, which is due to suppressed charge recombination and enhanced photon harvesting in the ternary photoactive layer because of **PTB7-Th**’s complementary light absorption of **PBZ1**:**L8-BO** binary films and increase in long-wavelength light absorption for the ternary films.

#### 2.1.2. FTAZ-Conjugated Polymers with Other Donor Moieties

**Figure 29:**
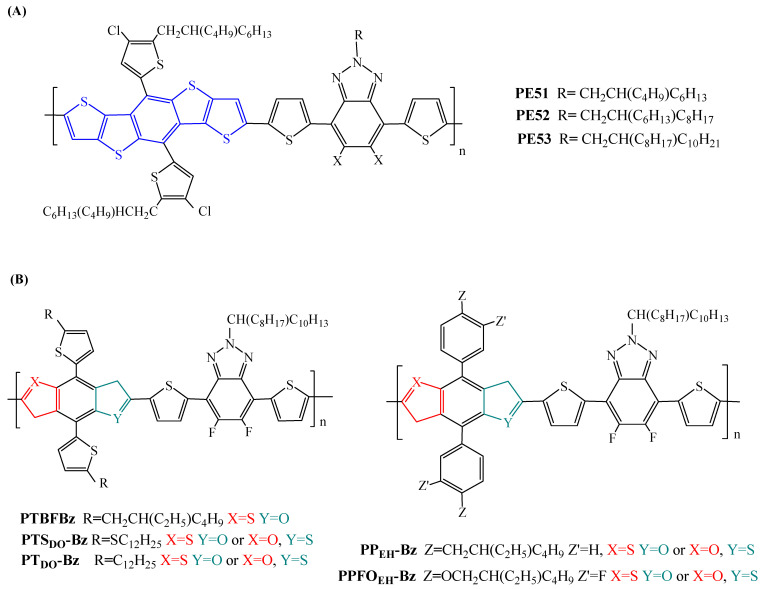
Structural formula of (**A**) DTBDT (evidenced in blue) [101] and (**B**) BDO (evidenced in red and green)-based polymers [102,103].

A further development of the research for the construction of D-A photovoltaic polymers was to make changes to the largely used BDT building block by extending the coplanar length via condensation of a thiophene unit to each BDT thienyl group to produce dithieno[2,3-d;2′,3′-d′]benzo[1,2-b;4,5-b′]dithiophenes (DTBDT), Figure 29A [101], or by substituting one thienyl ring with furan to produce thieno[2,3-f ]benzofuran (BDO), Figure 29B (Table 7) [102,103].

Much more studied than the asymmetric BDT derivative, even if not as much as the BDT system, is the BDT furan analogue benzo[1,2-b:4,5-b′]difuran (BDF). The replacement of the thiophene unit with furan possessing a smaller size may result in the formation of more planar and rigid structures with favorable inter/intramolecular interactions, tighter packing, smaller reorganization energy, and better self-assembly behavior than their BDT based counterparts. The stronger electronegativity of the oxygen atom in furan than the sulfur atom in thiophene endows BDF-based polymers with lower HOMOs that are preferred for high V_OC_, and the slight blueshift on absorption of the resulted BDF-based polymers is also expected in comparison with that of BDT-based polymers. Moreover, furan can be easily obtained from extensive bio-renewable sources, i.e., vegetables, leaves, and crops, at low cost [104].

**Figure 30:**
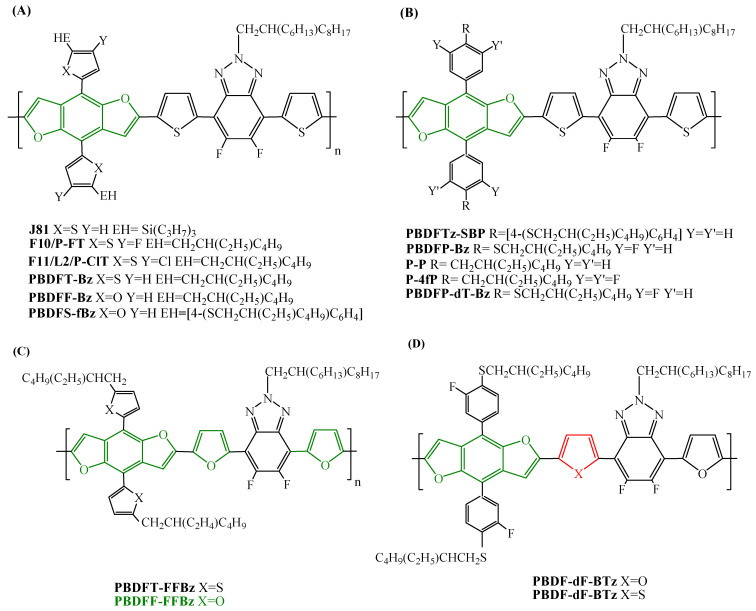
Structural formula of BDF-FTAZ-based polymers [26,62,67,105,106,107,108,109,110,111,112]: (**A**) Structural formula of **J81**, **F10/P-FT**, **F11**/L2**/P-ClT, PBDFT-Bz**, **PBDFF-Bz**, **PBDFF-fBz**, (**B**) Structural formula of **PBDFTz-SBP, PBDFP-Bz**, **PBDFT-FFBz**, **PBDFF-FFBz**, **P-P**, **P-4fP**, **PBDFP-dT-Bz**, (**C**) Structural formula of **PBDFT-FFBz**, **PBDFF-FFBz**, (**D**) Structural formula of **PBDF-dF-BTz, PBDF-dF-BTz**.

**Figure 31:**
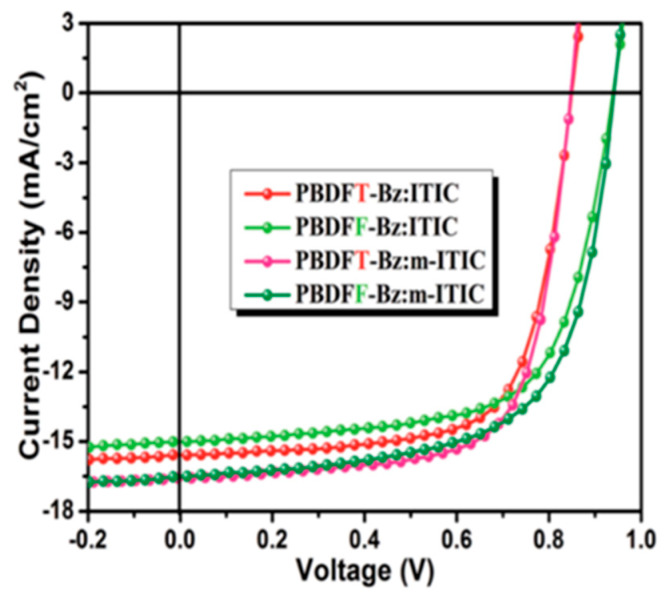
The J-V curves of **PBDFF-Bz**:**m-ITIC**- (light green line) and **PBDFT-Bz**:**m-ITIC**-based dark green line) devices recorded under AM1.5G (100 mWcm^−2^) illumination reproduced from ref. [105] with permission of the Royal Society of Chemistry.

BDF-based polymers can be distinguished by the substituents linked to the BDF phenyl ring which consist of aromatic pentacycle or aryl groups (Figure 30); for the first time, furyl derivatives were used as side-chain groups [26,105] and the corresponding polymers blended with ITIC or m-ITIC allowed for better performance than the analogues with the thienyl group (**PBDFF-Bz**:**m-ITIC**-based device PCE of 10.28% with respect to **PBDFT-Bz**:**m-ITIC**-based device PCE of 9.84%) (Table 8) (J-V curves are shown in Figure 31) [105]. This can be ascribed to the fact that **PBDFF–Bz** possesses a lower HOMO energy level and stronger p-p stacking than **PBDFT–Bz**.

In 2019 Zhu prepared an “all-furan” polymer, **PBDFF-FFBz** (Figure 30C): **PBDFT-FFBz: m-ITIC** devices showed an appreciable PCE (8.79%) (Table 8) [106].

**Figure 32:**
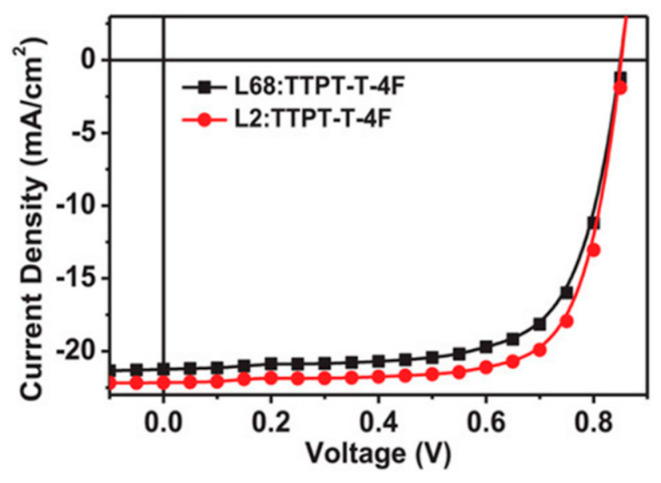
The J-V curve of the **L2**:**TTPT-T-4F**-based device (red line) recorded under AM1.5G (100 mWcm^−2^) illumination [67] © 2019 WILEY-VCH Verlag GmbH & Co. KGaA, Weinheim.

BDF-based polymers containing thienyl side-chain groups produced good performance as well [62,67,107], thanks to functionalization with halogen and in particular with chlorine (**L2**:**TTPT-T-4F**-based device with PCE of 14.0%, Table 8, the J-V curve is shown in Figure 32) [67].

**Figure 33:**
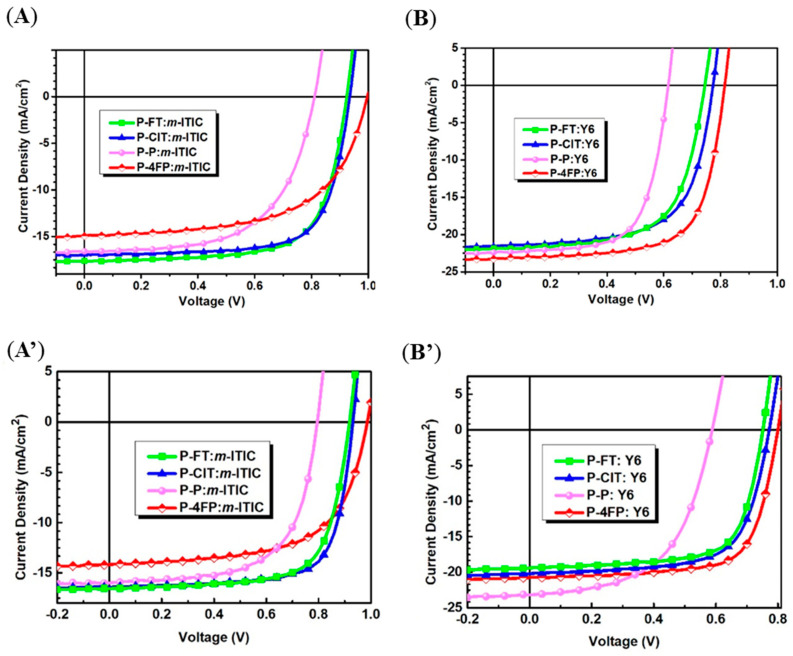
The J-V curve of (**A**) as-cast devices based on m-ITIC blended with polymers **P–FT**, **P–ClT**, **P–P**, and **P–4FP**; (**B**) as-cast devices based on Y6 blended with polymers **P–FT**, **P–ClT**, **P–P**, and **P–4FP**; (**A’**) aged devices based on m-ITIC blended with polymers **P–FT**, **P–ClT**, **P–P**, and **P–4FP**; (**B’**) aged devices based on Y6 blended with polymers **P–FT**, **P–ClT**, **P–P**, and **P–4FP** recorded under AM1.5G (100 mWcm^−2^) illumination adapted from ref. [108] with permission of the American Chemical Society © 2021 American Chemical Society.

Zheng et al. [108] reported interesting research on polymers **P-FT** (**F10**) and **P-ClT** (**L2**/**F11**), showing that the PCE of devices fabricated with **P-FT** and **P-ClT** blended with m-ITIC or Y6 after aging improved (Table 8): the J-V curves of the corresponding devices are reported in Figure 33. Moreover, the PCEs from the devices processed under ambient condition only possessed 0.3–2% loss compared to those devices under inert conditions, which indicates more stability and utility for practical applications.

The same was observed for **P-P** and **P-4FP** (Figure 30B) [108], with the last polymer PCE raised up to 13.34% with Y6 (the J-V curves of the corresponding devices are shown in Figure 33) [108].

Polymers **P-P** and **P-4FP** are characterized by the presence of an aryl group on BDF; actually, BDF-based polymers with an aryl side-chain group on BDF were developed successively, taking advantage of the fact that with large-conjugated phenyl side-chain the coplanarity of the polymer backbone is further increased, enhancing the aggregation tendency of polymer chains (Figure 30D, Table 8) [108,109,110,111,112].

**Figure 34:**
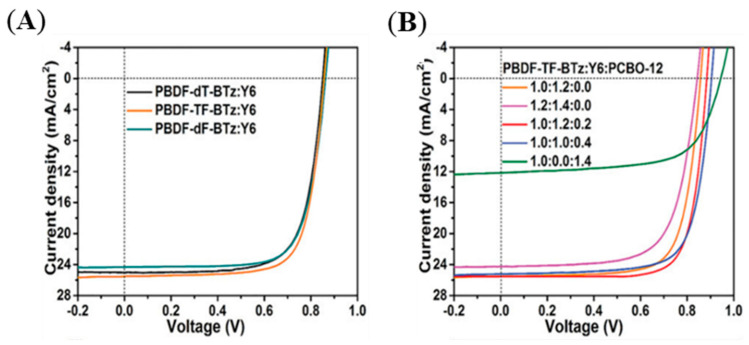
The J-V curves of (**A**) devices based on Y6 blended with polymers **PBDF-dT-Btz**, **PBDF-TF-Btz**, and **PBDF-dF-Btz** and (**B**) devices based on Y6:PCBO-12 in various ratios blended with **PBDF-TF-BtZ** (best performance corresponds to red line) recorded under AM1.5G (100 mWcm^−2^) illumination [112] © 2023 Wiley-VCH GmbH.

In particular, the most recent research on BDF-based polymers regards the development of polymers containing (2-ethylhexyl)(2-fluorophenyl)sulfane side-chain polymers; Gao et al. [112] modified the polymer backbone via engineering of the p-bridge to enhance their ɛ_r_, dielectric constant, which affects the charge dynamics process. In this way, the asymmetric **PBDF-TF-BTz** (Figure 30D) demonstrated a larger ɛ_r_, 4.22, than **PBDF-dT-BTz** with a symmetric thiophene p-bridge (3.15) (Figure 30B) and **PBDF-dF-BTz** with a symmetric furan p-bridge (3.90) (Figure 30D). The OSC fabricated with **PBDF-TF-BtZ** and Y6 showed a power conversion efficiency of 17.01% which increased up to 18.1% with an FF of 80.11% when a fullerene derivative (PCBO-12, Figure S10) was introduced as a third component (the J-V curves of the corresponding devices are shown in Figure 34).

**Scheme 5:**
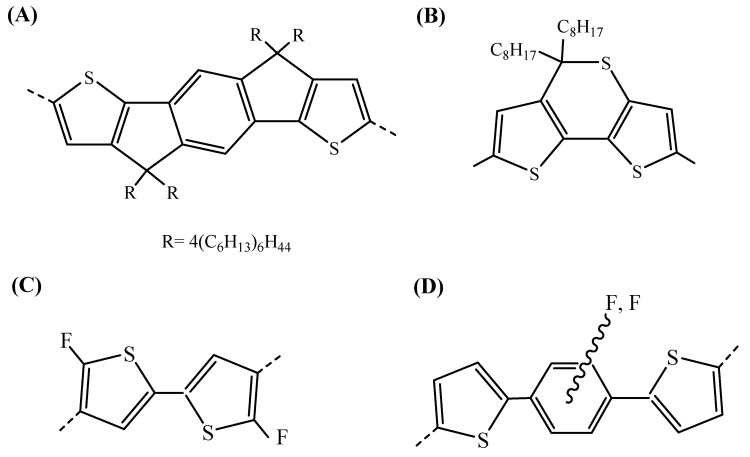
Structural formula of donor moieties: (**A**) indacenodithiophene; (**B**) dithienothiapyrane; (**C**) fluorinated thiophenes; (**D**) thiophenes intercalated with a difluorinated aryl group.

There are only very few more D-A FTAZ-based polymers containing D moieties (Scheme 5) which do not possess analogous structure to BDT; they consist of indacenodithiophene [113], dithienothiapyran [114], and thiophenes functionalized with fluorine [15] or intercalated with a difluorinated aryl group [115] in which the structure–property relationship and the importance of tuning morphology and crystallinity have been particularly evidenced.

#### 2.1.3. BzT-Based Conjugated Polymers with Other Bridges

**Figure 35:**
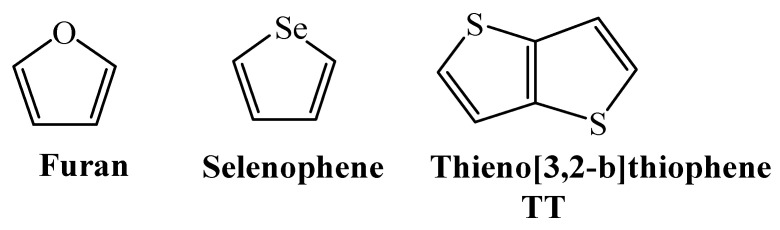
Structural formula of furan, selenophene, and thieno[3,2-b]thiophene.

Furan, selenophene, and thieno[3,2-b]thiophene (TT) (Figure 35) are the other bridging units which have been used to construct BzT-based conjugated polymers.

While furan has been used twice as bridge [105,112] and selenophene just once [116], the TT molecule has been the object of more intensive research, in particular by Zhou’s group.

Actually, TT is characterized by interesting optical and electrochemical properties due to its centrosymmetric, coplanar, and rigid structure, providing redshifted absorption, low bandgap, and high charge mobility compared to thiophene-containing polymers due to high delocalization of π-electrons and better intermolecular π-stacking interactions [117].

**Figure 36:**
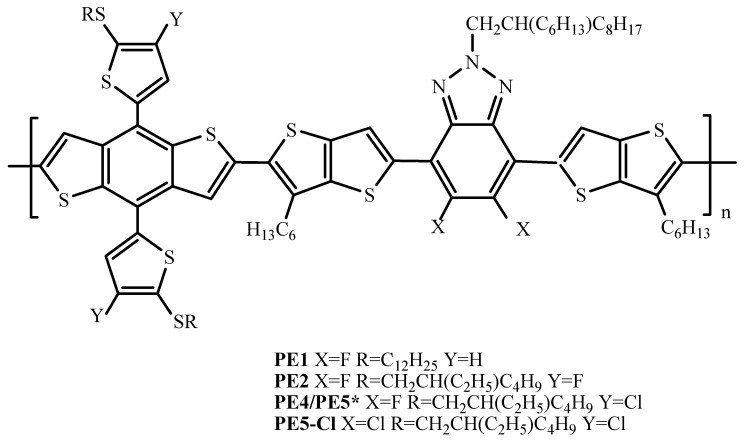
Structural formula of BDTT-alt-TT-BzT polymers [29,57,77,118]; * the reader must pay attention that the same polymer has very similar name.

The first approach was to prepare polymers analogous to **J61** and **J52** (Figure 36) [29,118].

**Figure 37:**
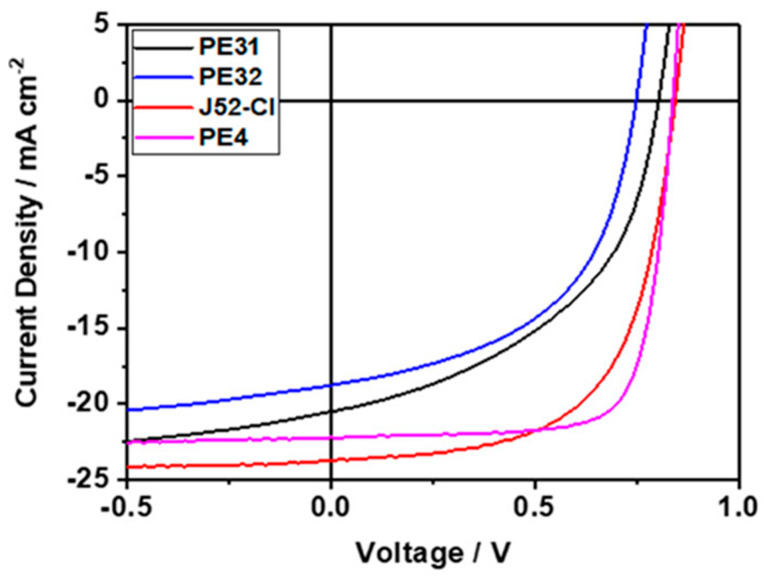
The J-V curves of the **PE4:Y6**-based device (pink line) and **J52-Cl:Y6**-based device (red line) recorded under AM1.5G (100 mWcm^−2^) illumination reproduced from ref. [29] with permission of the American Chemical Society © 2019 American Chemical Society.

In particular, the **PE4(PE5)**:**Y6**-based device has higher PCE (14.02%) (Table 9) than the **J52-Cl**:**Y6**-based one with quite high FF (75.4%), mainly as a consequence of the backbone conformation which changed from a zig-zagged type to a linear type with a little influence on the planarity (the J-V curves are shown in Figure 37) [29]. This conformation is more helpful in forming ordered interchain packing, resulting in obviously enhanced crystallinity and charge mobility.

All-chlorinated polymer **PE5-Cl** showed a poorer PCE than the corresponding **PE5**(**PE4**) (Table 9) because of the twisted backbone and weak p-p stacking induced by the dichlorination on the benzotriazole [57].

**Figure 38:**
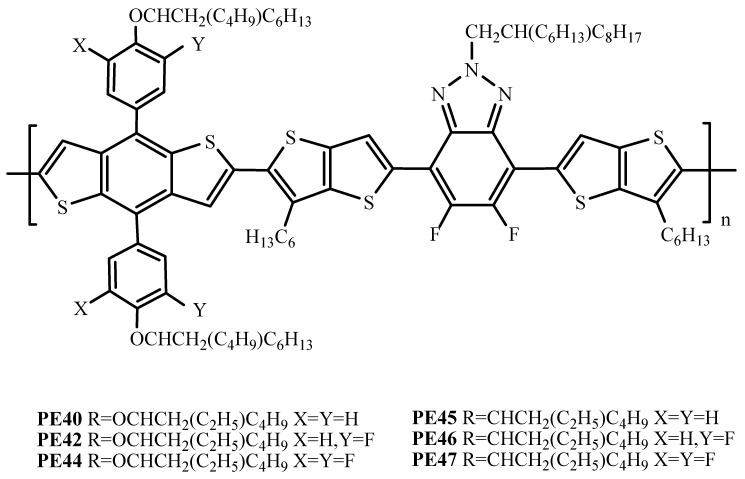
Structural formula of aryl BDT-alt-TT-BzT polymers [119,120].

**Figure 39:**
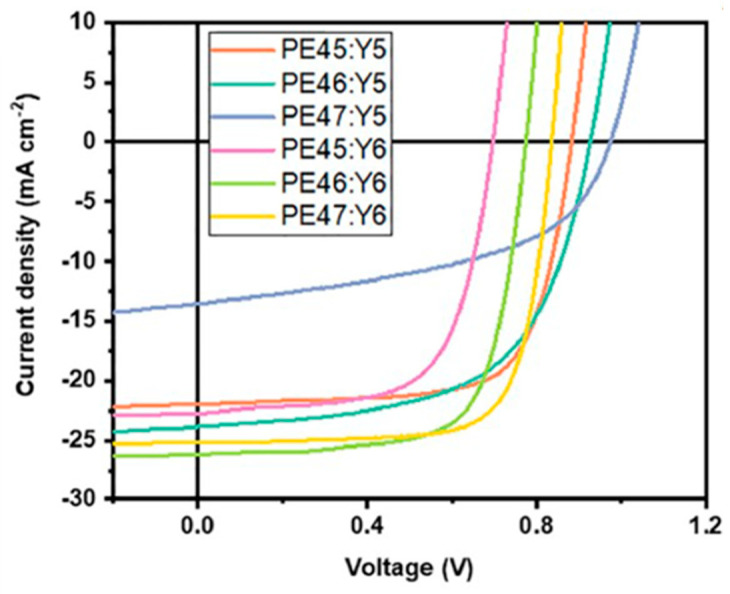
The J-V curves of devices based on Y6 blended with polymers **PE45**, **PE46**, and **PE47** and of devices based on Y6 blended with polymers **PE45**, **PE46**, and **PE47** recorded under AM1.5G (100 mWcm^−2^) illumination reproduced from ref. [120] with permission from Springer Nature © Science China Press 2023.

More recently, by substituting the thienyl group on BDT with a phenyl ring with different levels of fluorine substituent [119,120], the PCE increased up to 15.58% (Figure 38, Table 9); in particular, the different trend of PCE with Y5 and Y6 of the polymers **PE45**, **PE46**, and **PE47** is due to a better miscibility of Y6 with the more fluorinated species (the J-V curves are shown in Figure 39) [120].

Given the beneficial effects of halogenation also for TT-based BzT polymers and the superior optoelectronic properties to their non-halogenated counterparts, it is worth pointing out that the introduction of halogens have drawbacks such as additional and expensive synthesis steps and risks to human health and the ecological environment. Therefore, as an alternative to fluorine, Wang et al. [58] developed halogen-free donor polymers based on dicyanobenzotriazole in which the introduction of the cyano group actually lead to lowered energy levels, redshifted absorption, and improved photovoltaic performance in binary OSCs with Y6.

**Figure 40:**
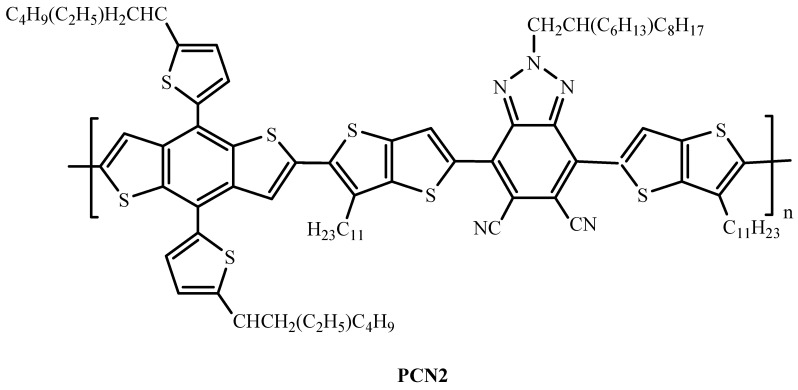
Structural formula of **PCN2** [58].

In particular with respect to **PCN1**, which has the same structure as **PCN2** (Figure 40) but with thiophene as the bridge instead of the TT derivative, **PCN2** had stronger π-π stacking and better charge transport, resulting in **PCN2**:**Y6** devices with higher and more balanced carrier mobility, less exciton recombination loss, suitable phase separation size, and thus higher PCE (Table 9).

### 2.2. Condensed Ring BzT-Based Conjugated Polymers

The utilization of building blocks with enlarged planarity for the preparation of *p*-type conjugated polymers can provide enhanced electron-delocalization and intermolecular interaction which may determine better photovoltaic performance.

**Figure 41:**
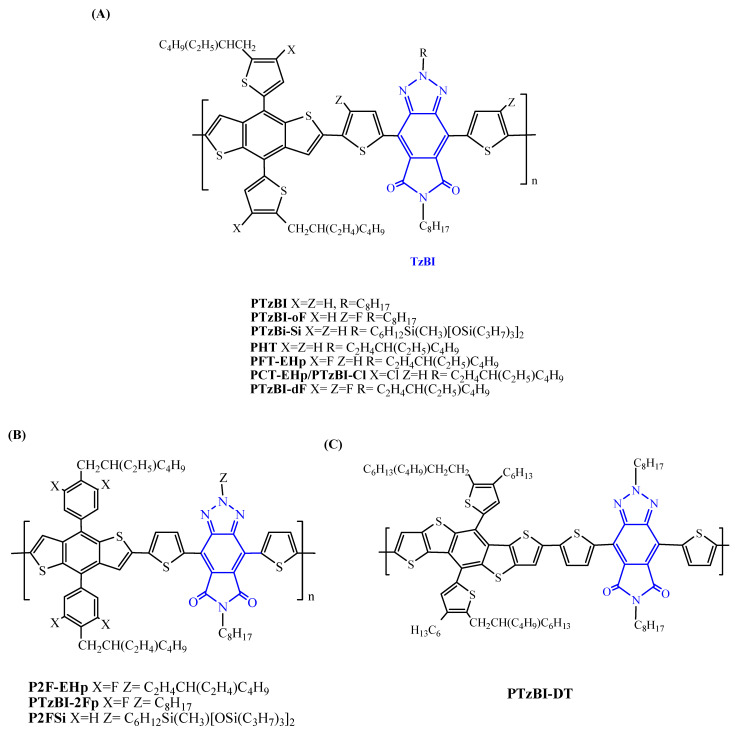
Structural formula of D-A TzBI-based polymers where the TzBI unit is indicated in blue [121,122,123,124,125,126,127,128,129,130,131,132,133,134]: (**A**) Structural formula of **PTzBI**, **PTzBI-oF, PTzBi-Si**, **PHT**, **PFT-EHp**, **PCT-EHp/PTzBI-Cl**, **PTzBI-dF**, (**B**) Structural formula of **P2F-EHp**, **PTzBI-2Fp**, **P2FSi**, (**C**) Structural formula of **PTzBI-DT**.

The pyrrolo[3,4-f]benzotriazole-5,7-dione (TzBI) unit developed by Cao’s group is the main BzT-fused-type building block used to construct conjugated polymers with BDTT derivatives (Figure 41A,B) except for the one with DTBDT, **PTzBI-DT** (Figure 41C) [121].

It is interesting to report that polymers **PTzBI** (Figure 41A) [121,122,123,124], **PTzBI-oF** (Figure 41A) [125], **PTzBI-Si (**Figure 41A) [126,127,128], and **P2F-Si** (Figure 41B) [128]—the last two with a siloxane group introduced to increase the solubility without disturbing the intermolecular stacking regarding the branched alkyl-side-chains—have been blended with NFAs also with not-halogenated solvents such as tetrahydrofuran [127], 2-MeTHF [122,125,126,127], cyclopentyl methyl ether (CPME) [127,128], and limonene (LM) [128] which are more environmentally friendly, determining appreciable PCE (Table 10).

In order to improve the photovoltaic performance, the octyl chain bonded to the azole nitrogen was substituted with the branched 3-ethylheptyl [124,129,130,131,133,135] or siloxane groups [126,127,128], the bridging thiophene or the BDTT thienyl group was halogenated [124,130,132,133,134,135], or the thienyl group bonded to BDT was substituted by difluoro aryl groups [128,129,130,132] (Table 10).

**Figure 42:**
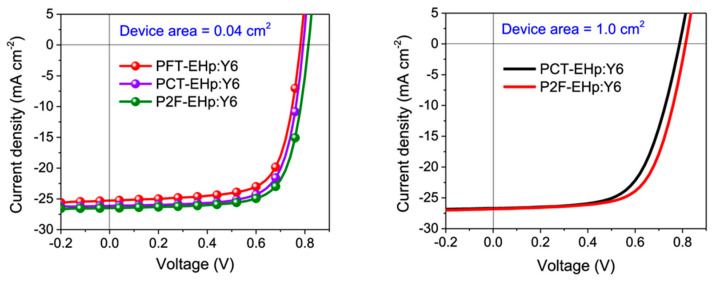
The J.V curves of devices based on Y6 blended with polymers **P2F-EHp** with different device areas (green line and red line) recorded under AM1.5G (100 mWcm^−2^) illumination reproduced from ref. [130] with permission of the American Chemical Society © 2020 American Chemical Society.

In particular, Fan et al. [130] constructed a device using **P2F-EHp** (Figure 41B) as a donor and Y6 as acceptor which also achieved PCE above 14% in the case of a device area of >1.1 cm^2^ (aperture area of 1 cm^2^); the J-V curves of OSCs with device areas of 0.04 and 1.0 cm^2^ are reported in Figure 42.

Actually, by engineering the electrical properties of the device with the introduction of a metallic frame (similar to the interconnection of solar modules) on the indium tin oxide (ITO) substrate, the charge extraction was enhanced and optoelectronic losses were minimized while maintaining the optical benefits, thus achieving high J_SC_ without sacrificing the FF of the device. Furthermore, the PCE was even increased above 16% via the addition of PC61BM which helped in optimizing the microstructure.

**Figure 43:**
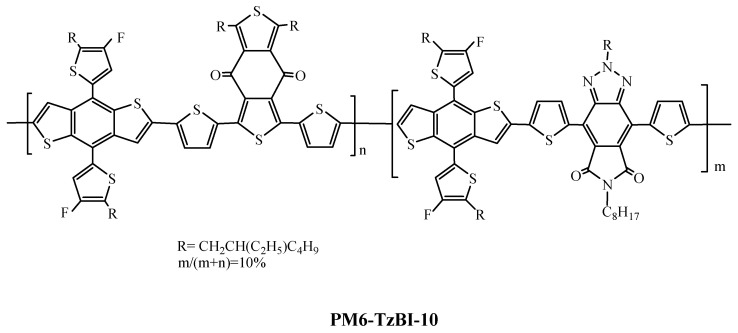
Structural formula of terpolymer **PM6-TzBI-10** [135].

Moreover, when **PTzBI** was incorporated into the high-performance donor polymer **PM6** to obtain terpolymers, the morphology was optimized gradually for improving charge generation and charge transport, also suppressing charge recombination [135]. Actually, the incorporation of the high-dipole and electron-deficient group of TzBI into the high-performance donor polymer introduces extra driving forces for crystallization by enhancing intermolecular interactions even if the additional segment in the terpolymer backbone inevitably introduces backbone disorder, which increases entropy. In this way, the device containing the terpolymer with 10% of **PTzBI** (**PM6-TzBI-10**) (Figure 43) blended with L8-BO exhibited a PCE of 18.36% (Table 10).

**Figure 44:**
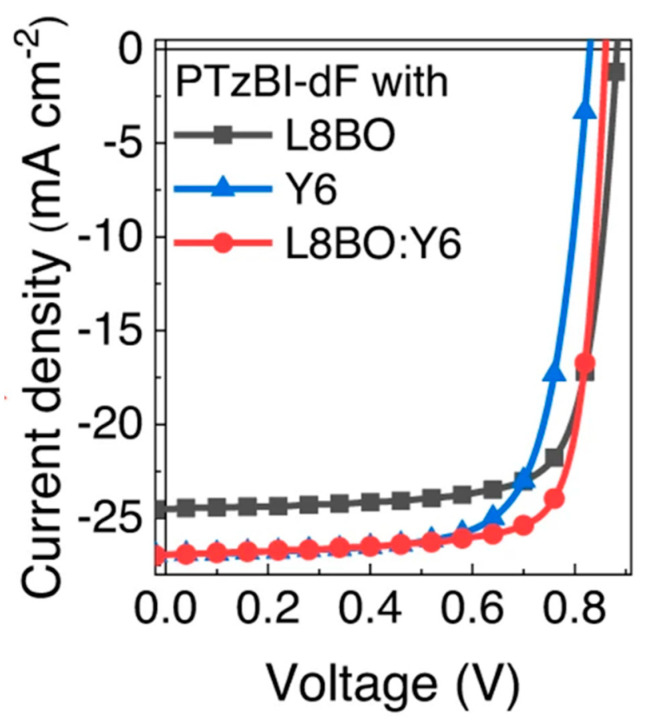
The J-V curves of devices based on **PTzBI-dF2F-EHp** blended with various NFAs recorded under AM1.5G (100 mWcm^−2^) illumination [134].

The regulation of the morphology of the BHJ photoactive layer is a crucial step in achieving high PCE, as recently reported by An et al. [134] who found that by combining L8BO:Y6 in an optimized ratio with the polymer donor **PTzBI-dF**, the corresponding device achieved a promising PCE of 18.26% (Table 10) (the J-V curves are shown in Figure 44). More importantly, the optimized OSCs could deliver excellent long-term thermal stability under 85 °C for 1400 h, which addresses the inherent thermal instability issues in state-of-the-art NFAs.

Another electron acceptor containing fused rings with benzotriazole is the thiophene-fused benzotriazole unit, developed by Chen’s group [136,137,138,139,140].

Considering that BzT-based polymers show absorption spectra in the region of 300–700 nm, an effective strategy to make full use of the sunlight is to reduce the bandgap of D–A conjugated systems via the stabilization of the population of the electronic quinoid state. Therefore, when thiophene is fused with the BzT unit, its aromaticity can stabilize the quinoid structure of the conjugated backbone and strengthen the intramolecular charge transfer to extend the absorption band for efficient light harvesting, thus improving the photocurrent of OSCs.

**Figure 45:**
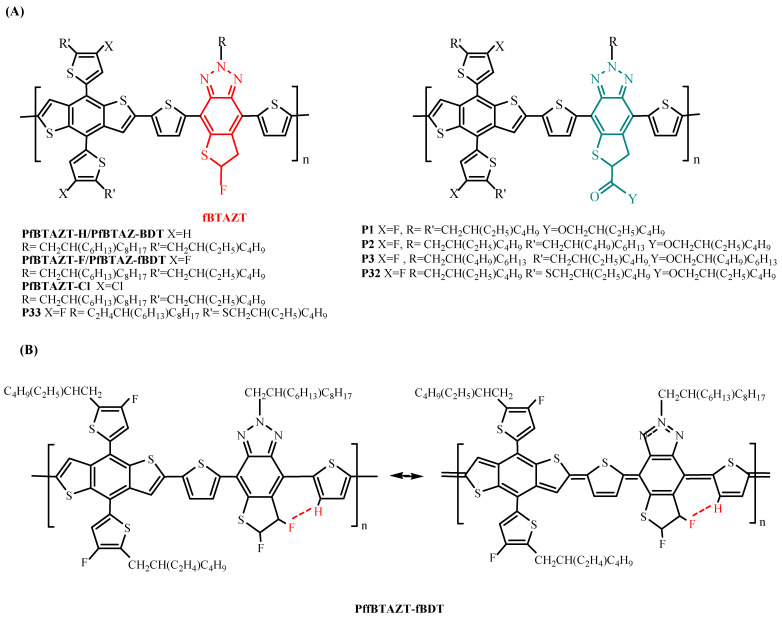
(**A**) Structural formula of D-A thiophene-fused benzotriazole-unit-based polymers [136,137,138,139,140,141,142]; (**B**) structural formula of **PffBTAZT-fBDT** and its quinoid form [140].

The thiophene-fused benzotriazole unit was combined with various BDT derivatives (Figure 45).

**Figure 46:**
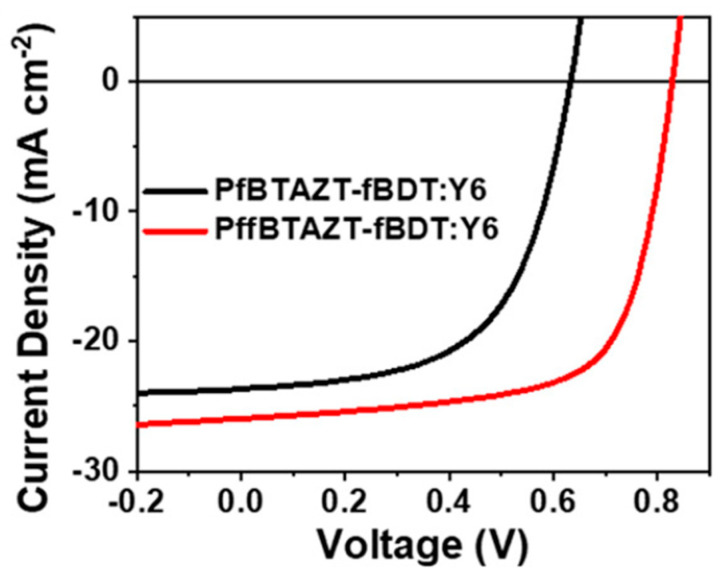
The J-V curves of the device based on **PfBTAZT-fBDT**:**Y6** (red line) recorded under AM1.5G (100 mWcm^−2^) illumination reproduced from ref. [140] with permission of the Royal Society of Chemistry.

The best PCE was obtained with **PffBTAZT-fBDT** (Figure 45B) blended with Y6 (PCE 14.53%, Table 10) (the J-V curves are shown in Figure 46) thanks to the subtle structural modification of thiophene-fused benzotriazole unit obtained introducing another F atom at the β-position of the fused thiophene ring of the fBTAZT unit [140]; actually, in this way, fluorine could build an “F^…^HC” non-covalent interaction with its adjacent thiophene ring as a “p-bridge” to partially lock the conformation of the conjugated backbone and strengthen the rigidity of the molecular skeleton, which is favorable for the light harvesting efficiency and the charge mobility which improve J_SC_. At the same time, the introduction of a strongly electronegative F atom could further reduce the HOMO energy level of the polymer donor, which is also favorable for the enhancement of V_OC_, J_SC_, V_OC_, and FF, which were increased from 23.68 to 25.43 mAcm^−2^, from 0.633 to 0.817 V, PCE from 58.46 to 69.94% and 8.77 to 14.53%, respectively, with respect to the parent mono-fluorine system.

**Figure 47:**
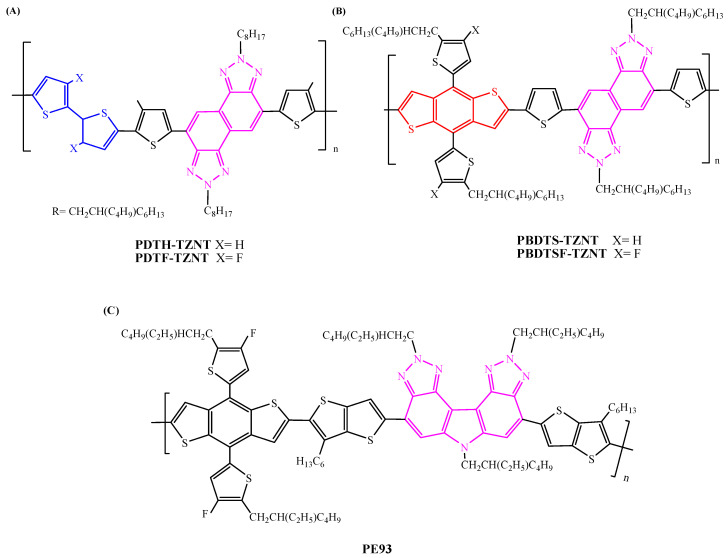
(**A**,**B**) Structural formula of D-A naphtotriazole-based polymers [141,142]. (**C**) Structural formula of **PE93** [143].

Very few more fused-ring BzT-based conjugated polymers consist of a benzotriazole unit condensed with two thiophens (**PY1**, Figure 3A) [19] and BDT derivatives (**PY39**, Figure 3A) [20] or of naphtobistriazole, where the two BzT benzogroups are condensed together, with thiophene as bridge and thiophenes (Figure 47A) [141] or BDT derivatives (Figure 47B) as donor [142] (Table 10).

The last and most recent copolymer **PE 93** (Figure 47C, Table 10) was obtained by linking a fluorinated derivative BDT, as the donor unit, through a TT molecule as bridge to an acceptor consisting of two BzT units fused in a pyrrole ring presenting seven nitrogen atoms that uniformly distribute frontier molecular orbital wave functions; the rigid conjugated ring could make the whole conjugated unit almost perfectly planar, the torsion angle between the two BzT units being only 0.21° [143].

## 3. BzT-Based Acceptor Polymers

Several non-fullerene acceptors used in OSCs are small molecules (SMAs) containing a BzT core; a very recent trend is to polymerize such a small molecule to prepare *n*-type narrow-bandgap polymer acceptors to be used in all-polymer solar cells.

Such a procedure has emerged as an effective way of enhancing both efficiency and morphological stability of all-polymer OSCs while also preserving the attractive merits of SMAs, such as high light absorbance in the NIR and efficient charge generation along with small E_loss_ in the corresponding devices [144].

**Figure 48:**
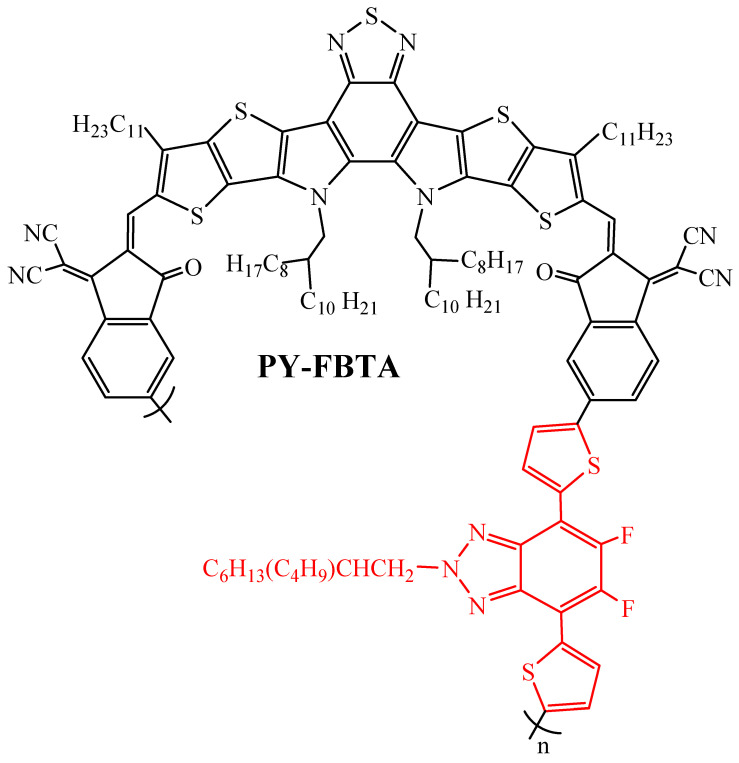
Structural formula of **PY-FBTA** [145].

**Figure 49:**
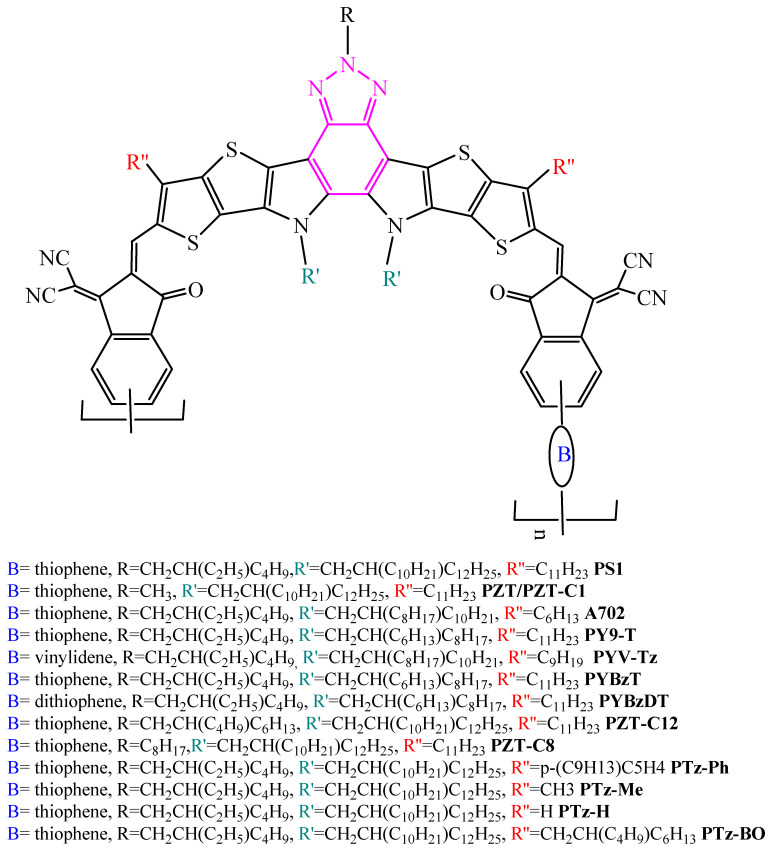
Structural formula of BzT-based acceptor polymers [125,144,145,148,149,150,151,152,153].

Except for the polymer **PY-FBTA** developed by Peng et al. [145] in 2023, in which an acceptor unit A (an Y6 derivative) is paired to a second acceptor unit A consisting of the well-known FTAZ, Figure 48, all of the research focused on BzT-based acceptor polymers which are obtained by connecting the non-fullerene acceptor building block of dithienothiophen[3,2-b]pyrrolobenzotriazole capped with 3-(dicyanomethylidene)-indan-1-one through thiophene [125,144,146,147,148,149,150,151], vinylidene [152], and dithiophene [153] (Figure 49, Table 11).

**Figure 50:**
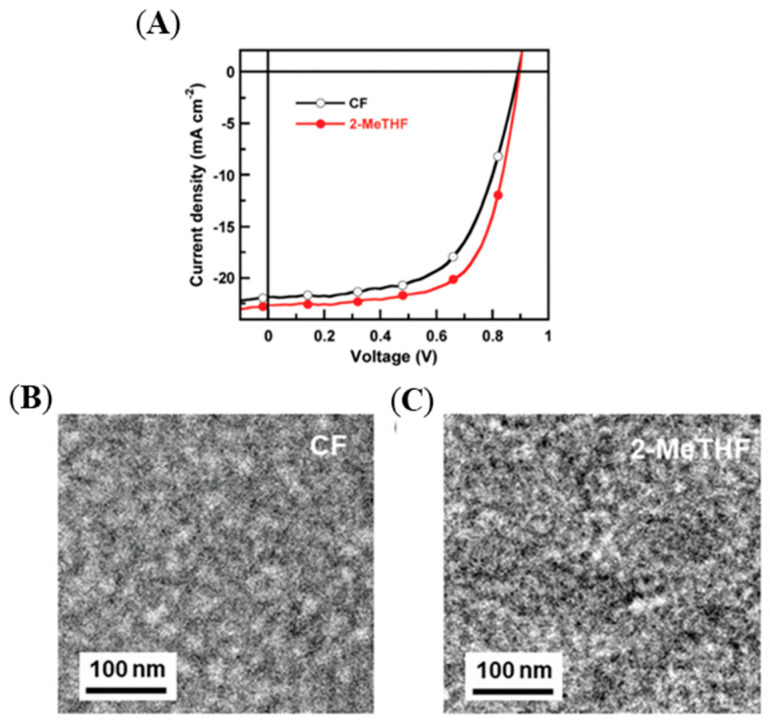
(**A**) The J-V curves of the devices based on **PTzBI-oF**:**PS16** processed from different solvents recorded under AM1.5G (100 mWcm^−2^) illumination; (**B**) TEM image of **PTzBI-oF**:**PS16** blend film processed from chloroform; (**C**) TEM image of **PTzBI-oF**:**PS16** blend film processed from 2-MeTHF [125].

The first acceptor, **PS1**, was designed with a bulky and solubilizing alkyl chain on the key building block so that it was possible to process it with the BzT-based copolymer PTzBI-oF (Figure 41A) using the non-halogenated solvent 2-MeTHF [125]. The corresponding device reached a significant PCE of 13.8%, even higher than the corresponding device obtained from chlorinated solvent (PCE 12.1% with chloroform) thanks to the more favorable film morphology induced by 2-MeTHF (Table 11) (the J-V curves are shown in Figure 50A and TEM images in Figure 50B): delicate and inter-continuous bright and dark regions were visible in both blends in the TEM images, but these features were finer in the 2-MeTHF-processed blend. Moreover, improved crystallinity and reduced-size domains in the 2-MeTHF-processed blend can assist in charge transfer and transportation and eventually increase the J_SC_ and FF of the OSCs.

Extending the conjugation through a dithiophene spacer (**PyBzDT**) did not help in improving the PCE of the device containing **J52** as donor polymer (Table 11) with respect to the “simple” thiophene-based acceptor (**PYBzT**) while the control of the regioregularity of polymer acceptors is important for improving the device performance, as shown by Fu et al. [144].

**Figure 51:**
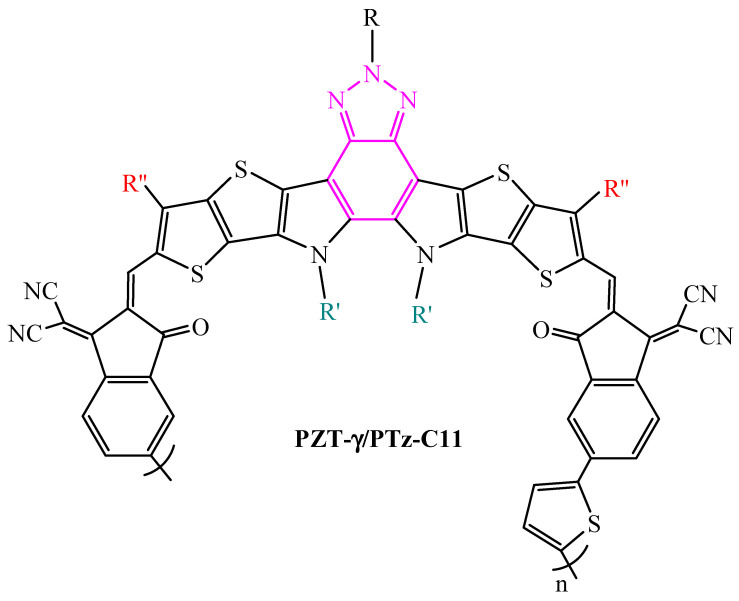
Structural formula of PZT-γ/PTZ-C11.

**Figure 52:**
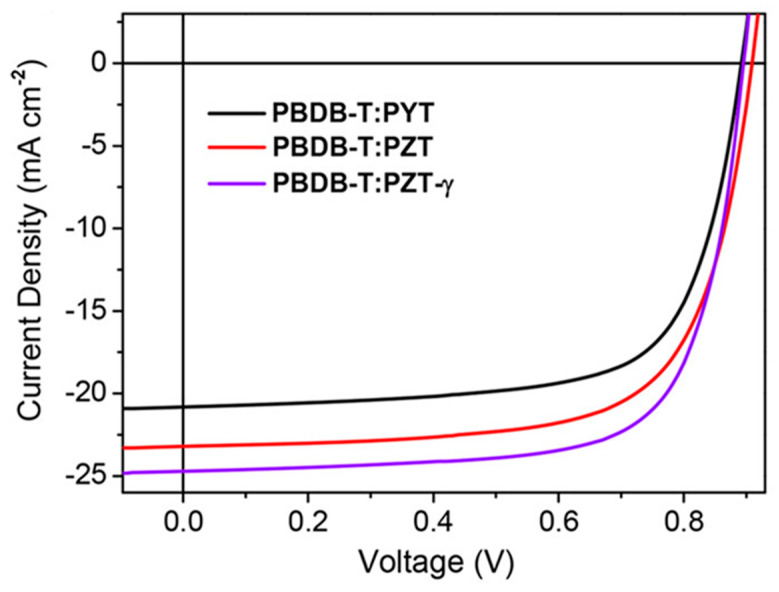
The J-V curves of the devices based on **PBDB-T**:**PZT-γ** (violet line) recorded under AM1.5G (100 mWcm^−2^) illumination [144] with permission of American Chemical Society© 2021 American Chemical Society.

Actually, through a thorough purification it was possible to isolate the regioregular **PZT-γ** species [144], reported later as **PTz-C11** [149] (Figure 51), that endows the polymer acceptor to have more extended and intense absorption and superior backbone ordering and form an optimal blend with the donor; in this way, PCE was increased from 14.5 to 15.8% thanks to higher J_SC_ and FF of devices with PBDB-T as donor polymer (the J-V curve is shown in Figure 52).

This PCE is expected to further increase up to 25.1% with Jsc 27.74 and Voc 0.986 V in a cell which utilizes a graded-index active layer containing the high-absorption-efficiency polymer PBDB compound with branched 2-butyl octyl, linear n-octyl, and methyl groups as well as a light-trapping anti-reflection coating thin film based on ITO to reduce incident light reflection and enhance its absorption [150].

A simple and feasible approach for material optimization via modulation of the structural conformation and interchain interaction and thereby the adjustment of optoelectronic properties and crystallization behavior is also the tuning of the side-chain substituents analogously to what is performed for polymer donors.

Fu et al. [148] varied N-alkyl chains on the BzT core including the branched 2-butyloctyl, linear n-octyl, and methyl groups. After decreasing the size of alkyl chains, the resulting **PZT** polymers exhibit better crystallinity, leading to higher charge carrier mobility in both neat and blended films. Consequently, the **PZT-C1** with the methyl group, the smallest one, is the best for all-polymer OSC applications and delivers higher PCE (14.9%) than the other two (PCE 13.1% and 13.8%) when paired with the polymer donor PBDB-T.

**Figure 53:**
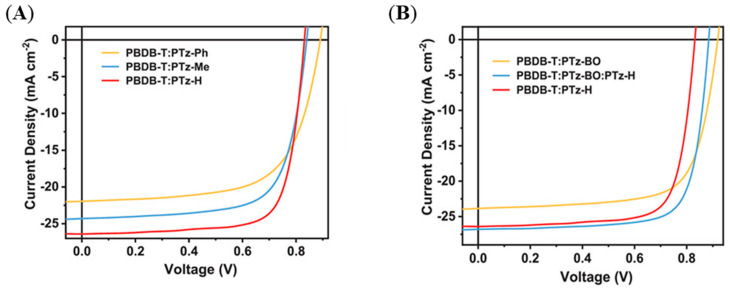
The J-V curves of the devices based on (**A**) **PBDB-T**:**PTz-Ph**, **PBDB-T**:**PTz-Me**, and **PBDB-T**:**PTzH** blends and (**B) PBDB-T:PTz-BO**, **PBDB-T**:**PTz-BO**:**PTzH**, and **PBDB-T**:**PTzH** blends recorded under AM1.5G (100 mWcm^−2^) illumination [151] © 2024 Wiley-VCH GmbH.

Analogously, Li et al. [151] prepared three polymer acceptors, **PTz-Ph**, **PTz-PMe**, and **PTz-H** (Figure 49), modifying the polymer backbone at the level of the external thienyl group by substituting the phenyl, methyl, and hydrogen in the β position of the thiophene unit. Weakening the steric hindrance resulted in stronger ICT effects and intermolecular interaction, as well as significant bathochromic absorption; photovoltaic performance of the corresponding OSC devices with PDBD-T as donor polymer was therefore improved due to higher Jsc and FF. Benefitting from the remarkable light harvesting, the polymer acceptor **PTz-H** performs well in the all-polymer OSC, delivering the highest PCE of 16.53% for all benzotriazole-based devices (the J-V curve is shown in Figure 53A). Moreover, the addition of **PTz-H** as a third component to the **PBDB-T:PTz-BO** binary system improved photon utilization in the NIR region (the J-V curves are shown in Figure 53B), so that the optimal ternary device exhibited an outstanding PCE of 18.16%, which represented one of the highest PCE values reported for all-polymer OSCs to date.

## 4. Conclusions

The importance of the photovoltaic technology based on polymers is that it represents a real alternative to fossil fuels for producing electricity, since it uses the most energetic renewable resource available.

This review reports the extensive studies in the past five/ten years which have led to the development of many new *p*- and *n*-type photoactive organic semiconductor materials starting from the benzotriazole moiety and should be helpful to the organic chemist to better understand how important is to find the right combination of donor and acceptor in terms of molecular orbital interactions, conductivity, miscibility, crystallinity, morphology, etc., to obtain a device with high PCE in order to take advantage of the tremendous synthetic efforts behind the research.

Fully aware that tailoring a device structure too is fundamental in the improvement of the OSC photovoltaic performance, this review illustrates how the goal of achieving better and better photovoltaic performance from the preparation of suitable BzT-based polymers is pursued from a chemical point of view, in particular by means of side-chain engineering rather than acting on the polymer backbone; actually, in the case of D-A conjugated polymers, most of the research was focused on the BDT-FTAZ skeleton.

However, the most recent trend of research regards the substitution of the simple thiophene with halogenated thiophene or with the more rigid and planar TT molecule as bridging unit between the BzT A unit and the D donor.

While other papers have already reviewed BzT-based D-A donor polymers and small-molecule-based NFAs, this review is the first to collect the successful work performed with BzT-based acceptor polymers, developed since 2021; such research is challenging but quite promising.

## Figures and Tables

**Table 2:** PCE values of **J52**:**NFA** OSCs under simulated AM1.5G (100 mWcm^−2^) illumination.

Polymer Donor	Non-Fullerene Acceptor	PCE_max_^a^ (%)	PCE_avg_^b^ (%)	References
**J52**	*ITIC*	5.18 (5.51) ^c^	4.85 ± 0.26 (5.26 ± 0.18) ^c^	[50]
**PBZ (J52)**	*ITIC*	8.00	7.5 ± 0.3	[51]
**J52**	*m-ITIC*	3.45 (5.98) ^c^	5.89 ± 0.12 ^c^	[53]
**J52**	*BTA13*	7.82	7.63 ± 0.015	[54]
**J52**	*ITIC*	3.78	3.01	[55]
**PBDT(T)FTAZ (J52)**	*N2200*	4.95 (5.83) ^d^ (6.14) ^c^	4.71 (5.55) ^d^ (5.95)^c^	[52]
**J52**	*JC2*	10.27	10.12 ± 0.09	[56]
**J52**	*Y6*	7.15		[57]
**J52**	*Y6*	6.02		[58]
**J52**	*IT-4F*	6.4		[43]

^a^ PCE maximum value, reported for as-cast film from halogenated solvents unless otherwise specified. ^b^ PCE average values and standard deviations when available. ^c^ After thermal treatment. ^d^ Film processed with DIO as additive.

**Table 3:** PCE values of **J52 derivative**:**NFA** OSCs under simulated AM1.5G (100 mWcm^−2^) illumination.

Polymer Donor	Non-Fullerene Acceptor	PCE_max_^a^ (%)	PCE_avg_^b^ (%)	References
**J91**	*m-ITIC*	6.05 (11.63) ^c^	11.32 ± 0.21 ^c^	[53]
**J52-F(PFBZ)**	*ITIC*	10.4	9.8 ± 0.4	[51]
**J52-F**	*BTA13*	8.36	8.28 ± 0.11	[54]
**J52-F **	*BTA3*	9.04	8.68	[64]
**J52-F **	*BTA4*	5.61	5.34	[64]
**J52-F **	*BTA5*	11.26	10.95	[64]
**J52-F**	*BTA3b*	9.81	9.72 ± 0.09	[65]
**J52-F **	*J14*	10.33	10.29 ± 0.04	[65]
**J52-Cl**	*ITIC*	11.53	10.9	[55]
**J52-Cl**	*Y6*	12.31	12.31 ± 0.06	[29]
**J52-Cl**	*IT-4F*	9.7	9.3	[43]
**L68 (J52-Cl)**	*IT-4F*	9.30	9.1 ± 0.2	[66]
**L68 (J52-Cl)**	*TTPT-T-4F*	12.72	12.42 ± 0.27	[67]
**J52-Cl**	*JC2*	11.44	11.21 ± 0.18	[56]
**J52-Cl**	*BTA1*	0.6		[68]
**J52-Cl**	*BTA11*	0.13		[68]
**J52-Cl**	*BTA3*	10.5		[68]
**J52-Cl**	*BTA13*	7.16		[68]
**J52-Cl**	*BTA7*	5.78		[68]
**J52-Cl**	*BTA17*	5.18		[68]
**J52-Cl**	*Y18*	13.64		[21]
**J52-Ome ^d^**	*JC2*	11.18	10.92 ± 0.16	[56]
**L24**	*IT-4F*	1.33	1.3 ± 0.1	[66]
**L810**	*IT-4F*	12.1	11.7 ± 0.4	[66]
**J52FTh**	*ITIC*	12.23 (13.32) ^c^		[69]
**J52ClF**	*IT-4F*	13.73 (14.59) ^c^	13.49 ± 0.24 (14.32 ± 0.27) ^c^	[59]
**J11**	*m-ITTC*	10.16 (12.32) ^c^	10.01 ± 0.24 (11.81 ± 0.21)	[70]
**J12**	*m-ITTC*	8.31 (8.74) ^c^	7.99 ± 0.13 (8.31 ± 0.25)	[70]
**J11**	*Y10*	11.37 (13.46) ^e^	11.17 ± 0.2 (13.26 ± 0.2)	[71]

^a^ PCE maximum value, reported for as-cast film from halogenated solvents unless otherwise specified. ^b^ PCE average values and standard deviations when available. ^c^ After thermal treatment. ^d^ Structural formula in Figure S7B. ^e^ Film processed with CN as additive after thermal treatment.

**Table 4:** PCE values of **alkylthio-substituted BDTT-alt-FTAZ:NFA** OSCs under simulated AM1.5G (100 mWcm^−2^) illumination.

Polymer Donor	Non-Fullerene Acceptor	PCE_max_ ^a^ (%)	PCE_avg_ ^b^ (%)	References
**J60**	*ITIC*	5.17 (8.97) ^c^	5.07 ± 0.14 (8.67 ± 0.31) ^c^	[50]
**J61**	*ITIC*	9.15 (9.53) ^c^	8.93 ± 0.26 (9.22 ± 0.24) ^c^	[50]
**J61**	*ITIC*	10.57	10.28 ± 0.15	[72]
**J61**	*ITIC*	9.32 ^c^		[75]
**J61**	*m-ITIC*	11.77	11.49 ± 0.16	[72]
**PSBZ**	*ITIC*	9.7 (10.5) ^c^	9.2 ± 0.4 (10.0 ± 0.3) ^c^	[73]
**J62**	*ITIC*	9.09 (10.81) ^c^	8.87 ± 0.19 (10.68 ± 0.11) ^c^	[74]
**J63**	*ITIC*	7.38 (8.13) ^c^	7.24 ± 0.13 (8.02 ± 0.09) ^c^	[74]
**J64**	*ITIC*	7.51 (8.59) ^c^	7.23 ± 0.19 (8.36 ± 0.18) ^c^	[74]
**J61**	*BTA1*	0.26	0.26 ± 0.003	[76]
**J61**	*BTA2*	3.02	2.98 ± 0.04	[76]
**J61**	*BTA3*	8.25	8.03 ± 0.14	[76]
**J52-FS**	*BTA13*	3.84	3.83 ± 0.02	[54]
**J52-FS**	*Y6*	10.58	10.14 ± 0.40	[77]
**J52-Cl-S**	*Y6*	10.48	10.23 ± 0.24	[57]
**J65**	*ITIC*	4.92 (6.91) ^c^	4.58 ± 0.32 (6.01 ± 0.36))	[61]
**PBDTFBTA-2T**	*Y6*	11.76	11.37 ± 0.35	[78]
**PBDTFBTA-TSi**	*Y6*	14.18	13.76 ± 0.40	[78]
**PBDTFBTA-2Si**	*Y6*	11.92	11.45 ± 0.39	[78]

^a^ PCE maximum value, reported for as-cast film from halogenated solvents unless otherwise specified. ^b^ PCE average values and standard deviations when available. ^c^ After thermal treatment.

**Table 5:** PCE values of **alkylsilyl-substituted BDTT-alt-XTAZ**:**NFA** OSCs under simulated AM1.5G (100 mWcm^−2^) illumination.

Polymer Donor	Non-Fullerene Acceptor	PCE_max_ ^a^ (%)	PCE_avg_ ^b^ (%)	References
**J71**	*ITIC*	9.03 (11.41) ^c^	8.94 ± 0.24 (11.2 ± 0.29)	[82]
**J71**	*m-ITIC*	12.05	11.77 ± 0.15	[83]
**J70**	*m-ITIC*	11.62	11.40 ± 0.18	[83]
**J72**	*m-ITIC*	10.23	9.95 ± 0.22	[83]
**J73**	*m-ITIC*	10.71	10.43 ± 0.16	[83]
**J74**	*m-ITIC*	9.63	9.47 ± 0.12	[83]
**J71**	*ITCPTC*	6.38 (7.13) ^c^	5.88 ± 0.52 (6.61 ± 0.67) ^c^	[84]
**J71**	*MeIC*	6.64 (8.83) ^c^	6.22 ± 0.47 (8.38 ± 0.56)	[84]
**J71**	*ITC6-IC*	11.32	10.89	[85]
**J71**	*IDIC*	11.75	11.23	[85]
**J71**	*Me-IC*	10.57	10.37	[85]
**J71**	*ITCPTC*	10.46	10.03	[85]
**J71**	*ITIC*	10.95	10.55	[85]
**PBZ-ClSi (J71-Cl)**	*IT-4F*	12.8 ^b^	12.5	[43]
**J71**	*IT-4F*	7.79 (8.16) ^d^		[30]
**J71-Cl (PBZ-ClSi)**	*IT-4F*	9.47 (11.10) ^e^		[30]
**J101**	*IT-4F*	8.07 (11.30) ^e^		[30]

^a^ PCE maximum value, reported for as-cast film from halogenated solvents unless otherwise specified. ^b^ PCE average values and standard deviations when available. ^c^ Film processed with toluene and 1-phenylnaphthalene as additive. ^d^ After thermal treatment. ^e^ Film processed with diphenyl ether as additive.

**Table 6:** PCE values of **aryl-substituted BDT-alt-FTAZ**:**NFA** OSCs under simulated AM1.5G (100 mWcm^−2^) illumination.

Polymer Donor	Non-Fullerene Acceptor	PCE_max_ ^a^ (%)	PCE_avg_ ^b^ (%)	References
**PDTTz-N**	*ITIC*	6.61		[92]
**PBDTTz-BP**	*ITIC*	8.03		[92]
**PBDTTz-BP**	*ITIC*	9.20	8.97	[47]
**PBDTTz-SBP**	*ITIC*	12.09	11.89	[47]
**P2**	*ITIC*	5.91 (6.59) ^c^		[94]
**PBZ1**	*ITIC*	4.3 (5.8) ^c^	4.1 (5.7) ^c^	[95]
**PBZ-m-CF3**	*ITIC*	7.9 (10.4) ^c^	7.8 (10.3) ^c^	[95]
**PBTA-PS**	*ITIC*	11.85	11.52	[98]
**PBTA-PSF**	*ITIC*	13.91	13.60	[98]
**PBTZa**	*ITIC-4Cl*	6.02 (8.50) ^c^	5.96 (8.31) ^c^	[99]
**PBTZb**	*ITIC-4Cl*	12.09 (14.53) ^c^	11.81 (14.34) ^c^	[99]
**T1**	*ITIC*	11.83	11.50 ±0.33	[93]
**DZ1**	*MeIC*	8.26	7.94 ± 0.19	[97]
**DZ2**	*MeIC*	10.21	9.98 ± 0.20	[97]
**DZ3**	*MeIC*	5.97	5.77 ± 0.20	[97]
**PBDTPSi-FTAZ**	*IDIC*	5.78 (7.07) ^c^	5.62 ± 0.16 (6.91 ± 0.16) ^c^	[100]

^a^ PCE maximum value, reported for as-cast film from halogenated solvents unless otherwise specified. ^b^ PCE average values and standard deviations when available. ^c^ After thermal treatment.

**Table 7:** PCE values of **DTBDT- and BDO-based polymers**:**NFA** OSCs under simulated AM1.5G (100 mWcm^−2^) illumination.

Polymer Donor	Non-Fullerene Acceptor	PCE_max_ ^a^ (%)	PCE_avg_ ^b^ (%)	References
**PE51**	*Y6*	13.34	13.17 ± 0.12	[101]
**PE52**	*Y6*	14.61	14.33 ± 0.28	[101]
**PE53**	*Y6*	13.72	13.52 ± 0.15	[101]
**PTBFBz**	*ITIC*	6.54 (8.33) ^c^	6.42 (8.12) ^c^	[102]
**PTS_DO_-Bz**	*ITIC*	8.66		[103]
**PT_DO_-Bz**	*ITIC*	6.59		[103]
**PP_EH_-Bz**	*ITIC*	8.23		[103]
**PPFO_EH_-Bz**	*ITIC*	8.16		[103]

^a^ PCE maximum value, reported for as-cast film from halogenated solvents unless otherwise specified. ^b^ PCE average values and standard deviations when available. ^c^ After thermal treatment.

**Table 8:** PCE values of **BDF-based polymers**:**NFA** OSCs under simulated AM1.5G (100 mWcm^−2^) illumination.

Polymer Donor	Non-Fullerene Acceptor	PCE_max_ ^a^ (%)	PCE_avg_ ^b^ (%)	References
**PBDFT-Bz**	*ITIC*	9.26	9.08	[105]
**PBDFT-Bz**	*m-ITIC*	9.84	9.62	[105]
**PBDFF-Bz**	*ITIC*	9.46	9.28	[105]
**PBDFF-Bz**	*m-ITIC*	10.28	10.02	[105]
**PBDFS-fBz**	*ITIC*	9.00		[26]
**PBDFT-FFBz**	*m-ITIC*	7.57	7.32 ± 0.20	[106]
**PBDFF-FFBz**	*m-ITIC*	8.79	8.32 ± 0.32	[106]
**J81**	*ITIC*	10.6		[107]
**J81**	*m-ITIC*	11.5		[107]
**L2 (F11/P-ClT)**	*TTPT-T-4F*	14.0	13.68 ± 0.21	[67]
**F10 (P-FT)**	*m-ITIC*	10.5		[62]
**F11 (L2/PClT)**	*m-ITIC*	11.37		[62]
**P-FT (F10)**	*m-ITIC*	11.43 ^c^	10.87 ± 0.23 ^a^	[108]
**P-FT (F10)**	*Y6*	10.61 ^c^	10.03 ± 0.48 ^a^	[108]
**P-ClT (L2/F11)**	*m-ITIC*	11.61 ^c^	11.04 ± 0.55 ^a^	[108]
**P-ClT (L2/F11)**	*Y6*	11.03 ^c^	10.64 ± 0.15 ^a^	[108]
**P-P**	*m-ITIC*	8.28 ^c^	7.32 ± 0.59 ^a^	[108]
**P-P**	*Y6*	9.49 ^c^	8.11 ± 0.11 ^a^	[108]
**P-4FP**	*m-ITIC*	8.86 ^c^	8.70 ± 0.26 ^a^	[108]
**P-4FP**	*Y6*	13.34 ^c^	12.71 ± 0.51 ^a^	[108]
**PBDFTz-SBP**	*ITIC*	12.42	12.24 ± 0.22	[109]
**PBDFP-Bz**	*ITIC*	11.10 ^d^	10.69 ± 0.27	[110]
**PBDFP-Bz**	*IT-M*	12.93 ^e^	12.19 ± 0.38	[110]
**PBDFP-Bz**	*Y6*	14.62	13.49 ± 0.62	[111]
**PBDFP-dT-Bz**	*Y6*	16.03		[112]
**PBDFP-dF-Bz**	*Y6*	15.59		[112]
**PBDFP-TF-Bz**	*Y6*	17.01		[112]
**PBDFP-TF-Bz**	*Y6:PCBO12*	18.1		[112]

^a^ PCE maximum value, reported for as-cast film from halogenated solvents unless otherwise specified. ^b^ PCE average values and standard deviations when available. ^c^ After aging. ^d^ Film processed with DIO additive, after thermal treatment and further treatment with methanol. ^e^ Film processed with DIO additive, after thermal treatment.

**Table 9:** PCE values of **BDTT-alt-TT-BzT polymers**:**NFA** OSCs under simulated AM1.5G (100 mWcm^−2^) illumination.

Polymer Donor	Non-Fullerene Acceptor	PCE_max_ ^a^ (%)	PCE_avg_ ^b^ (%)	References
**PE1**	*BTA3*	8.43	8.36 ± 0.09	[118]
**PE2**	*BTA3*	5.83	5.75 ± 0.05	[118]
**PE2**	*Y6*	13.50	13.05 ± 0.26	[77]
**PE4 (PE5)**	*Y6*	14.02	13.90 ± 0.08	[29]
**PE5 (PE4)**	*Y6*	14.48	14.25 ± 0.21	[57]
**PE40**	*Y6*	7.07	6.29 ± 0.86	[119]
**PE42**	*Y6*	10.11	9.93 ± 0.14	[119]
**PE44**	*Y6*	13.62	12.81 ± 0.50	[119]
**PE45**	*Y5*	13.76	13.40 ± 0.26	[120]
**PE46**	*Y5*	13.55	13.32 ± 0.19	[120]
**PE47**	*Y5*	6.51	6.14 ± 0.18	[120]
**PE45**	*Y6*	10.30	10.16 ± 0.12	[120]
**PE46**	*Y6*	14.25	13.72 ± 0.38	[120]
**PE47**	*Y6*	15.58	15.16 ± 0.38	[120]
**PCN1**	*BTA3*	12.07	11.63 ± 0.44	[58]
**PCN2**	*BTA3*	15.2	14.83 ± 0.37	[58]

^a^ PCE maximum value, reported for as-cast film from halogenated solvents unless otherwise specified. ^b^ PCE average values and standard deviations when available.

**Table 10:** PCE values of **TzBI-based polymers**:**NFA** OSCs under simulated AM1.5G (100 mWcm^−2^) illumination.

Polymer Donor	Non-Fullerene Acceptor	PCE_max_ ^a^ (%)	PCE_avg_ ^b^ (%)	References
**PTzBI-DT**	*ITIC*	8.11 (9.43) ^c^	7.99 (9.32) ^c^	[121]
**PTzBI**	*ITIC*	8.64 (10.23) ^d^	8.60 (10.13) ^d^	[121]
**PTzBI**	*N2200*	9.16 ^e^	9.06 ± 0.14 ^e^	[122]
**PTzBI**	*N2200*	8.21 ^f^	8.05 ± 0.15 ^f^	[123]
**PTzBI**	*Y6*	9.24	9.06 ± 0.18	[124]
**PTzBI-Si**	*N2200*	8.3 ^e^ (10.1) ^g^	8.2 ± 0.1 ^e^ (9.9± 0.2) ^g^	[126]
**PTzBI-Si**	*N2200*	10.1 ^e^	9.9 ± 0.2 ^e^	[127]
**PTzBI-Si**	*N2200*	7.6 ^g^	7.4 ± 0.2 ^g^	[127]
**PTzBI-Si**	*N2200*	11.0 ^h^	10.8 ± 0.2 ^h^	[127]
**PTzBI-Si**	*PNDICI*	5.4 ^i^ (3.0) ^j^	5.2 ± 0.2 ^i^ (2.8 ± 0.1) ^j^	[128]
**P2F-Si**	*PNDICI*	4.6 ^i^ (4.2) ^j^	4.4 ± 0.1 ^i^ (4.1 ± 0.1) ^j^	[128]
**P2F-EHP**	*BTPT-4F*	1.09 ^c^	1.06 ± 0.06 ^c^	[129]
**P2F-EHP**	*BTPTT-4F (Y6)*	16.02 ^d^	15.75 ± 0.25 ^d^	[129]
**P2F-EHP**	*Y6*	15.65	15.50 ± 0.15	[130]
**P2F-EHP**	*Y6+PC61BM*	16.18	16.07 ± 0.13	[130]
**P2F-EHP**	*BTA3*	4.62	4.45 ± 0.02	[131]
**P2F-EHP**	*BTA3-F*	8.38	8.25 ± 0.02	[131]
**PTzBI-2Fp**	*ITIC-4F+5% N2200*	13.0	12.9 ± 0.1	[132]
**PTzBIoF**	*PS1*	13.8 ^g^	13.5	[125]
**PM6-TzBI-10**	*L8-BO*	18.36		[135]
**PHT-EHp**	*Y6*	7.7 ^d^	7.63 ± 0.2	[133]
**PFT-EHp**	*Y6*	14.16	14.02 ± 0.13	[130]
**PFT-EHp**	*Y6*	15.4 ^d^	15.0 ± 0.2	[133]
**PCT-EHp (PTzBI-Cl)**	*Y6*	15.06	14.96 ± 0.06	[130]
**PCT-EHp (PTzBI-Cl)**	*Y6*	15.8 ^d^	15.6 ± 0.1	[133]
**PCT-EHp (PTzBI-Cl)**	*Y6DT*	16.4 ^d^	16.2 ± 0.2	[133]
**PTzBI-Cl (PCT-EHp)**	*Y6*	10.35	9.99 ± 0.36	[124]
**PTzBI-dF**	*Y6*	9.13	8.77 ± 0.36	[124]
**PTzBI-dF**	*L8-BO*	16.74 ^k^	16.49 ± 0.25	[134]
**PTzBI-dF**	*Y6*	16.23 ^k^	16.03 ± 0.20	[134]
**PTzBI-dF**	*L8-BO+Y6*	18.26	17.95 ± 0.31	[134]
**P1**	*ITIC*	7.14 ^l^	7.02 ± 0.08	[136]
**P2**	*ITIC*	4.17 ^l^	3.96 ± 0.14	[136]
**P3**	*ITIC*	3.66 ^l^	3.60 ± 0.07	[136]
**PfBTAZT-BDT (PfBTAZT-H)**	*ITIC*	6.04	5.71	[137]
**PfBTAZT-fBDT (PfBTAZT-F)**	*ITIC*	6.59 ^d^	6.05 ^d^	[137]
**PfBTAZT-H (PfBTAZT-BDT)**	*BTA3*	6.65	6.53 ± 0.16	[138]
**PfBTAZT-F (PfBTAZT-fBDT)**	*BTA3*	7.69	7.53 ± 0.09	[138]
**PfBTAZT-Cl**	*BTA3*	8.00	7.95 ± 0.06	[138]
**P32**	*IHIC*	8.59 ^m^		[139]
**P33**	*IHIC*	6.63 ^m^		[139]
**PfBTAZT-fBDT (PfBTAZT-F)**	*Y6*	8.77	8.50 ± 0.14	[140]
**PffBTAZT-fBDT**	*Y6*	14.53	14.29 ± 0.16	[140]
**PY1**	*IT-M*	12.49	12.20 ± 0.30	[19]
**PY39**	*Y18*	12.45	12.26 ± 0.19	[20]
**PDTH-TZNT**	*IT-M*	4.42	4.05 ± 0.37	[141]
**PDTF-TZNT**	*IT-M*	10.05 (11.48) ^n^	9.74 ± 0.31 (11.16 ± 0.32) ^n^	[141]
**PDTS-TZNT**	*ITIC*	10.45	10.13 ± 0.32	[142]
**PDTS-TZNT**	*IT-4F*	11.31	11.00 ± 0.31	[142]
**PDTSF-TZNT**	*ITIC*	12.16	11.81 ± 0.35	[142]
**PDTSF-TZNT**	*IT-4F*	13.25	12.92 ± 0.33	[142]
**PE93**	*Y6*	9.32	9.28 ± 0.09	[143]
**PE93**	*BTA75*	10.11	10.05 ± 0.11	[143]
**PE93**	*BTA76*	12.16	11.98 ± 0.18	[143]

^a^ PCE maximum value, reported for as-cast film from halogenated solvents unless otherwise specified. ^b^ PCE average values and standard deviations when available. ^c^ Film processed with dibenzylether (DBE) and DIO additives. ^d^ Film processed with DBE additive. ^e^ Film processed with 2-MeTHF. ^f^ Film processed under 30% humidity. ^g^ Film processed with 2-MeTHF after thermal treatment. ^h^ Film processed with CPMe. ^i^ Film processed with THF. ^j^ Film processed with LM. ^k^ Film processed with DBE additive after thermal treatment. ^l^ Film processed with diphenylether (DPE). ^m^ Film processed with DIO additive after thermal treatment. ^n^ Film processed with 5,5,10,10,15,15-hexabutyl-2,7,12-tri(4-(3-ethylhexyl-4-oxothiazolidine-2-yl)dimalononitrile-benzothiadiazole)-truxene (meta-TrBRCN) additive.

**Table 11:** PCE values of **donor polymer**:**BzT-based acceptor polymer** OSCs under simulated AM1.5G (100 mWcm^−2^) illumination.

BzT-Acceptor	Polymer Donor	PCE_max_ ^a^ (%)	PCE_avg_ ^b^ (%)	References
**PS1**	*PTzBI-of*	13.8 ^c^	13.8 ^c^	[125]
**PS1**	*PTzBI-of*	12.1 ^d^	12.1 ^d^	[125]
**PYT**	*PBDB-T*	12.9	12.7 ± 0.2	[144]
**PZT (PZT-C1)**	*PBDB-T*	14.5	14.2 ± 0.3	[144]
**PZT-γ (PTz-C11)**	*PBDB-T*	15.8	15.6 ± 0.2	[144]
**Graded index(GI)-PZT**	*PBDB-T*	25.1	25.1	[150]
**PYV-Tz**	*PBDB-T*	13.02	12.78 ± 0.24	[152]
**PYBzT**	*J52*	11.22	10.67 ± 0.28	[153]
**PYBzDT**	*J52*	8.93	8.69 ± 0.26	[153]
**PZT-C12**	*PBDB-T*	13.1	12.8 ± 0.3	[148]
**PZT-C8**	*PBDB-T*	13.8	13.5 ± 0.3	[148]
**PZT-C1 (PZT)**	*PBDB-T*	14.9	14.5 ± 0.4	[148]
**PTz-BO**	*PBDB-T*	15.76	15.56 ± 0.19	[149]
**PTz-C11 (PZT-γ)**	*PBDB-T*	15.59	15.36 ± 0.22	[149]
**PTz-BO:PTz-C11**	*PBDB-T*	16.58	16.34 ± 0.22	[149]
**PY-FBTA**	*PM6*	13.95	13.74	[145]
**PTz-H**	*PBDB-T*	16.53	16.28 ± 0.19	[151]
**PTz-Me**	*PBDB-T*	14.48	14.22 ± 0.22	[151]
**PTz-Ph**	*PBDB-T*	12.82	12.56 ± 0.25	[151]
**PTz-BO:PTz-H**	*PBDB-T*	18.16	17.86 ± 0.25	[151]

^a^ PCE maximum value, reported for as-cast film from halogenated solvents unless otherwise specified. ^b^ PCE average values and standard deviations when available. ^c^ Film processed with 2-MeTHF. ^d^ Film processed with CHCl_3_.

## Supplementary Materials

The following supporting information can be downloaded at: https://www.mdpi.com/article/10.3390/molecules29153625/s1.
