# Supplementary material for: The Double-Cross of Benzotriazole-Based Polymers as Donors and Acceptors in Non-Fullerene Organic Solar Cells
Source: Molecules. 2024 Jul 31;29(15):3625. doi: 10.3390/molecules29153625 (PMC11313701; doi:10.3390/molecules29153625)
Supplement: Supplementary file 1 [file molecules-29-03625-s001.zip › molecules-3088206-supplementary.pdf]

The double-cross of benzotriazole based polymers as donors and acceptors in non-fullerene organic solar cells

Laura Crociani

Institute of Condensed Matter Chemistry and Technologies for Energy, ICMATE, National Research Council of Italy, CNR
Corso Stati Uniti 4
35127 Padua
Italy
laura.crociani@cnr.it

SUPPORTING INFORMATION

Figure S1 Structural formula of NFAs reported in the text

SF-PDI2

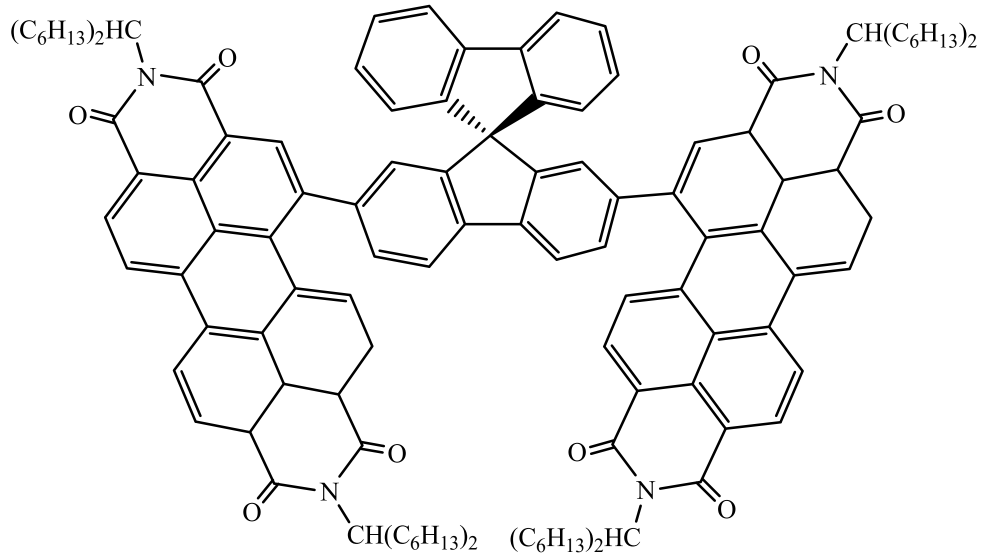

IDCIC

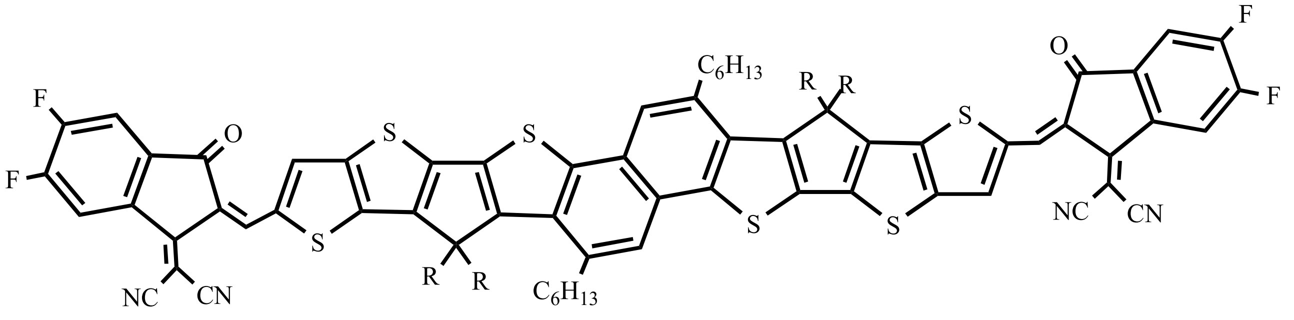

$R=p\text{-}C_6H_4\text{-}C_6H_{13}$

ITIC (ITIC 1)

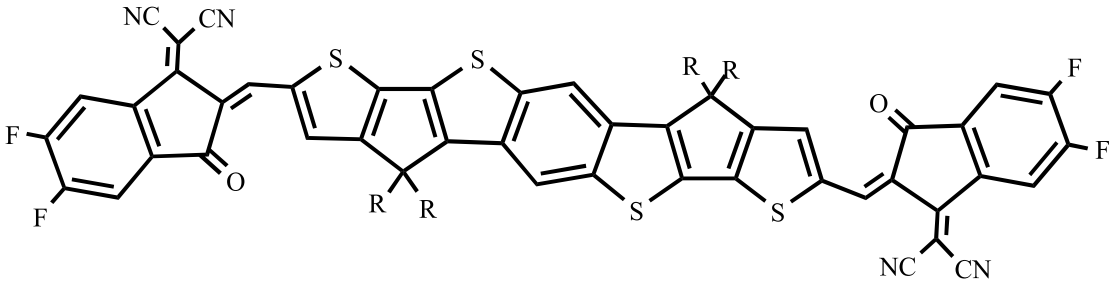

R=*p*-C_6H_4-C_6H_{13}

ITIC2

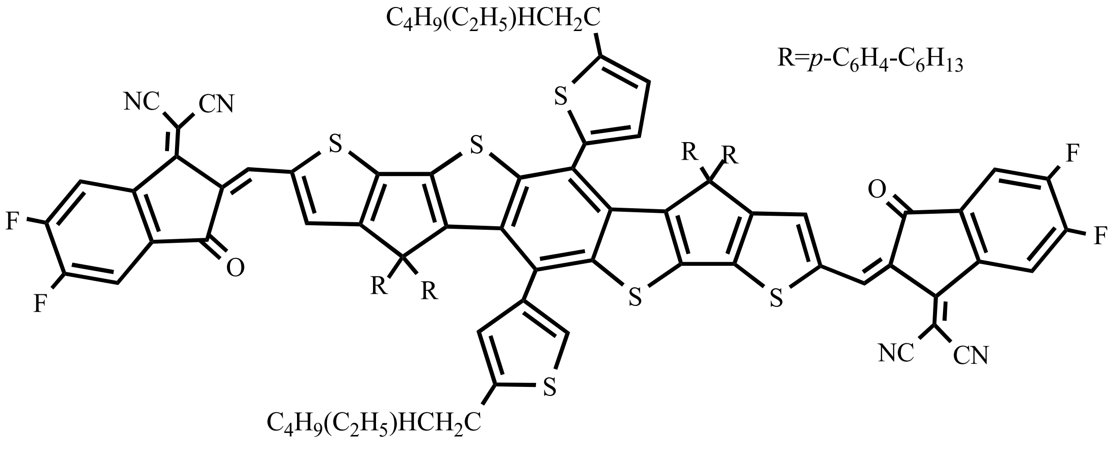

ITIC-Th, ITIC-Th1

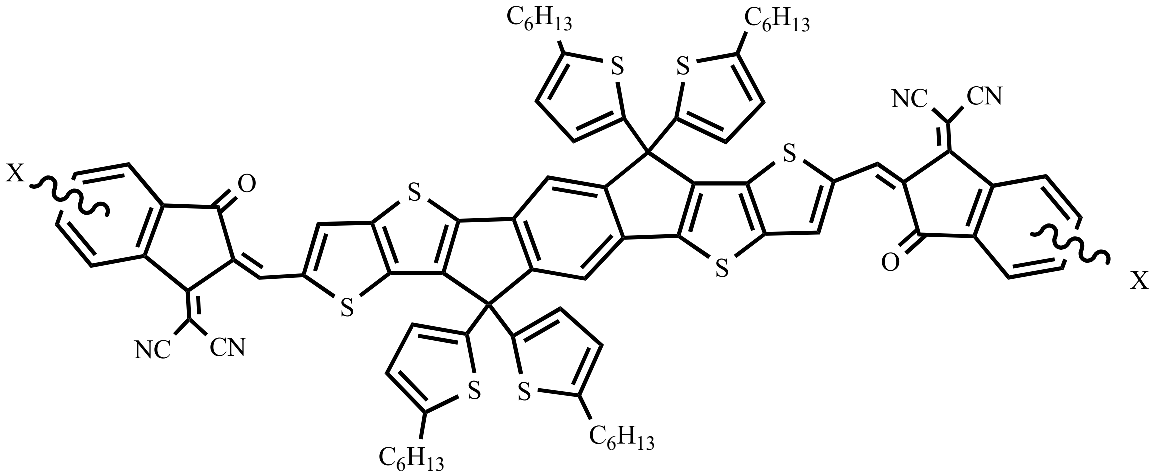

ITIC-Th X=H
ITIC-Th1 X=F

ITIC-4Cl

R=*p*-C_6H_4-C_6H_{13}

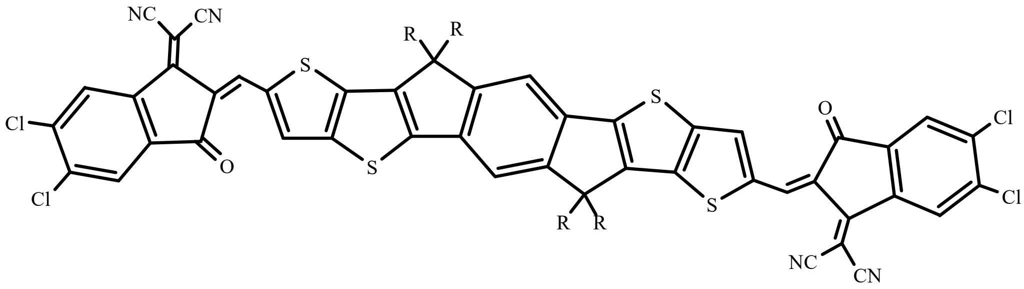

INIC, INIC1,2,3,

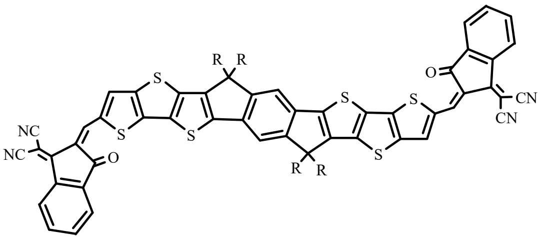

INIC

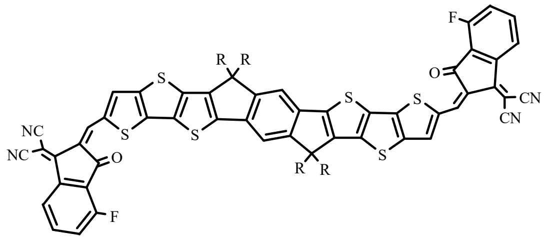

INIC1

R=*p*-C_6H_4-C_6H_{13}

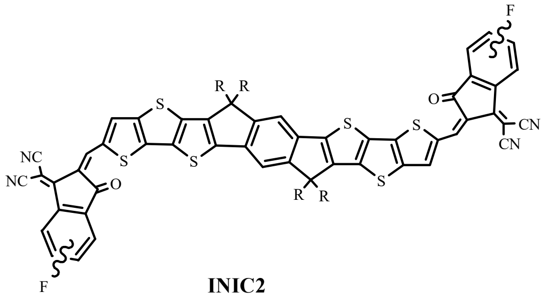

INIC2

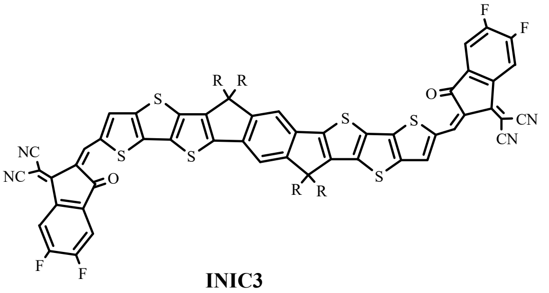

INIC3

IOIC2

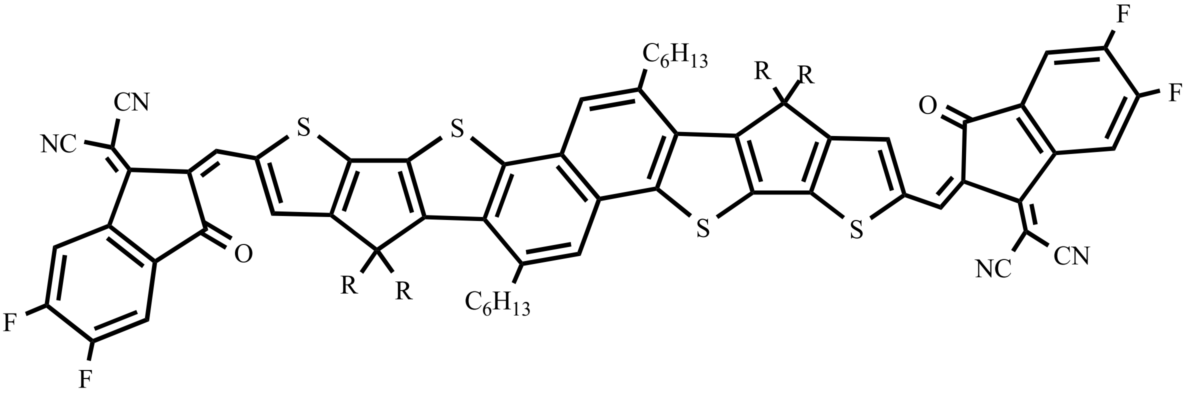

R=*p*-C_6H_4-C_6H_{13}

IHIC2

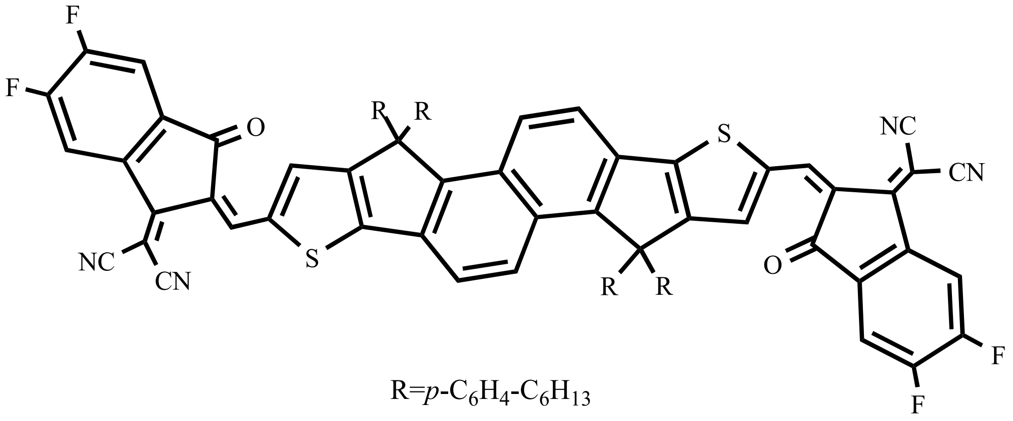

R=*p*-C_6H_4-C_6H_{13}

IT-M

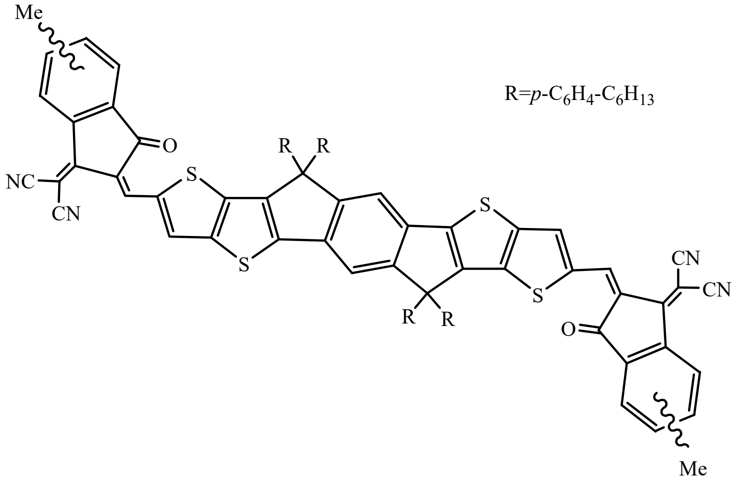

R=*p*-C_6H_4-C_6H_{13}

IDIC

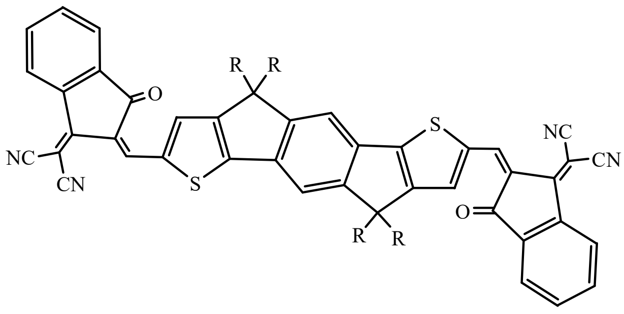

N2200

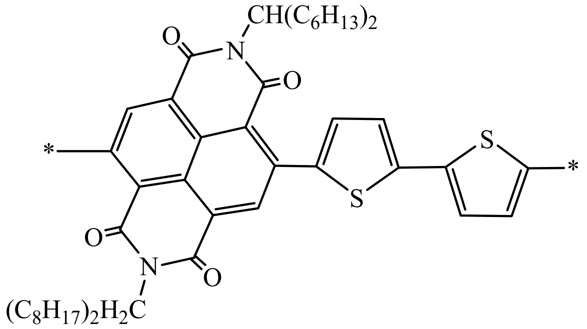

JC14

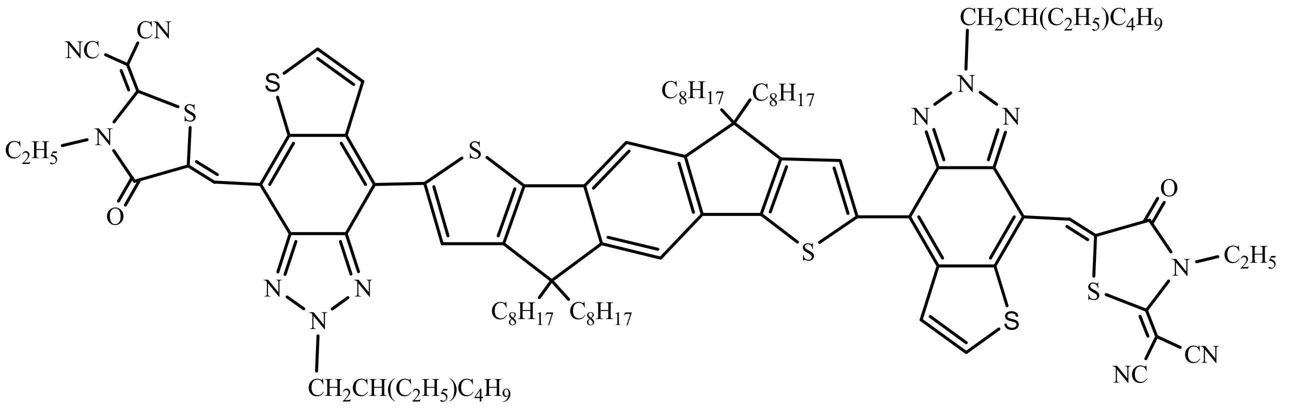

BTA13

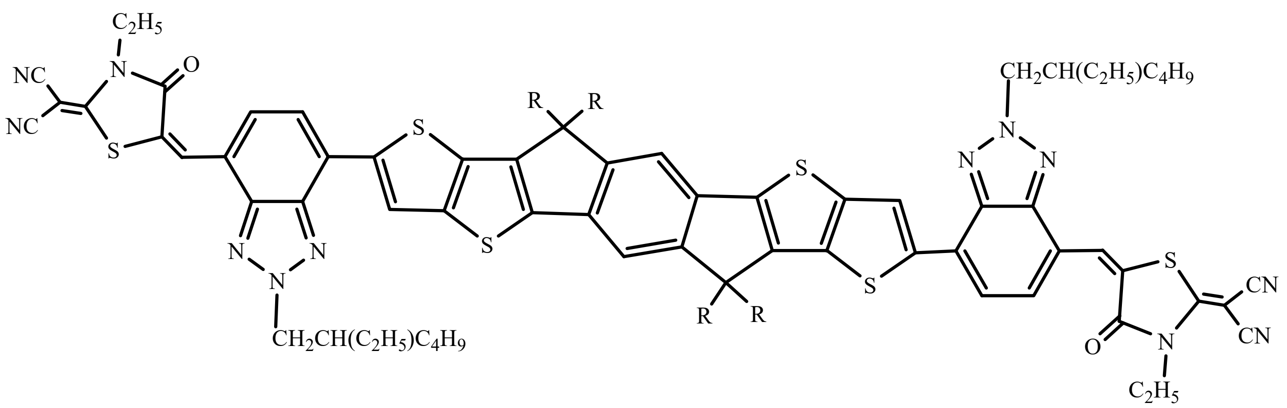

R=p-C_6H_4-C_6H_{13}

BTA3b

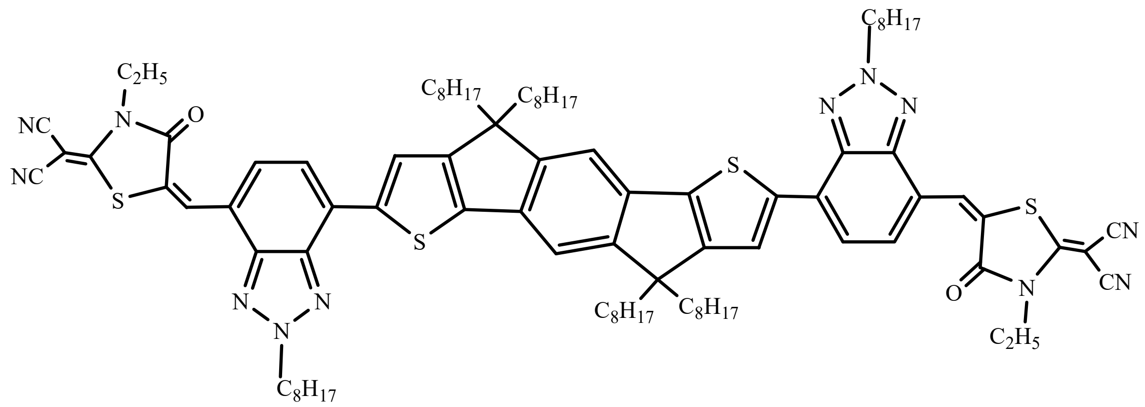

BTA4, BT5

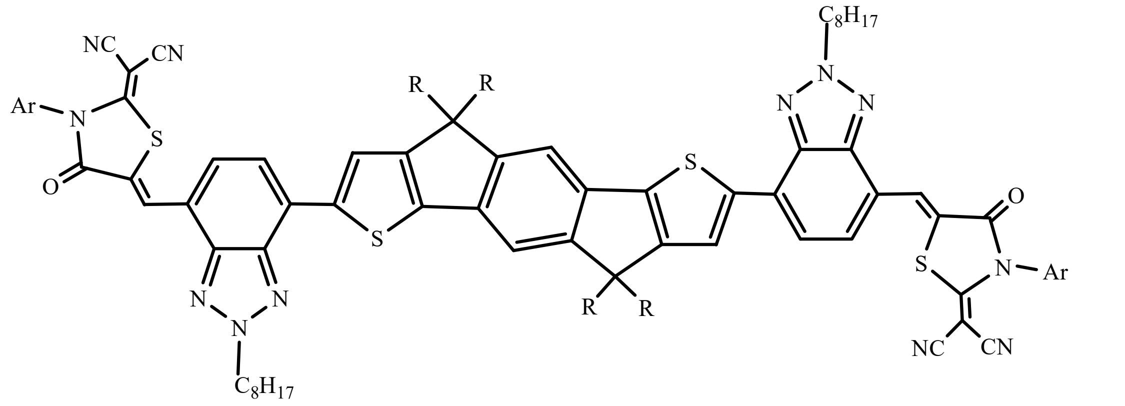

BTA4 R=*p*-C_6H_4-C_6H_{13}Ar=C_6H_4

BTA5 R=*p*-C_6H_4-C_6H_{13}Ar=$CH_2(C_6H_4)$

JC2

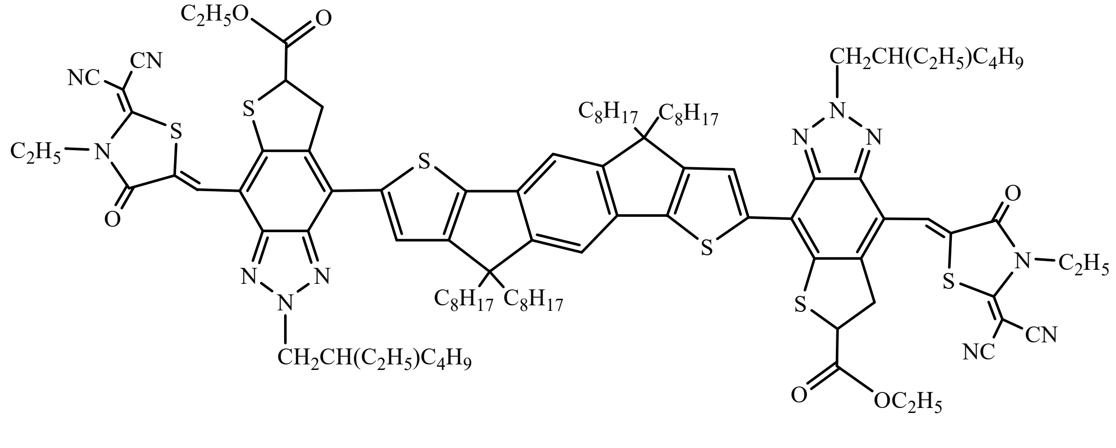

IT-4F

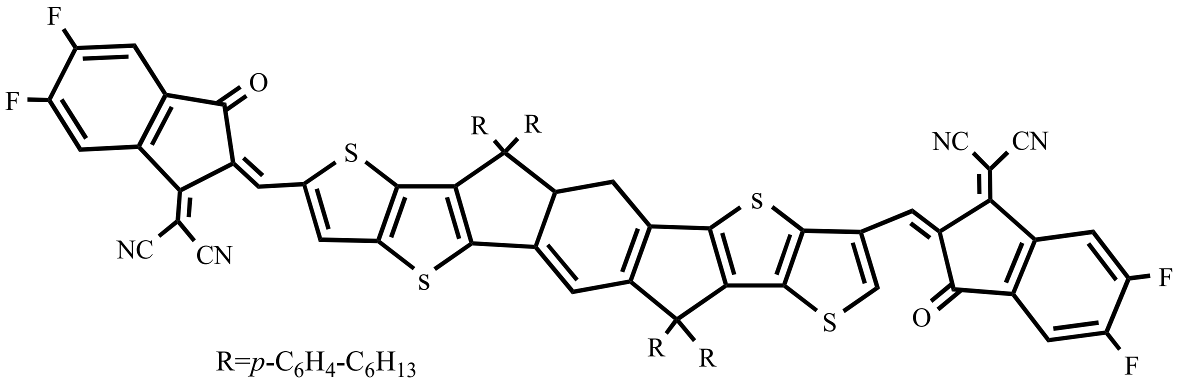

R=p-C_6H_4-C_6H_{13}

BTA1, BTA3, BTA7, BTA11, BTA13, BTA17

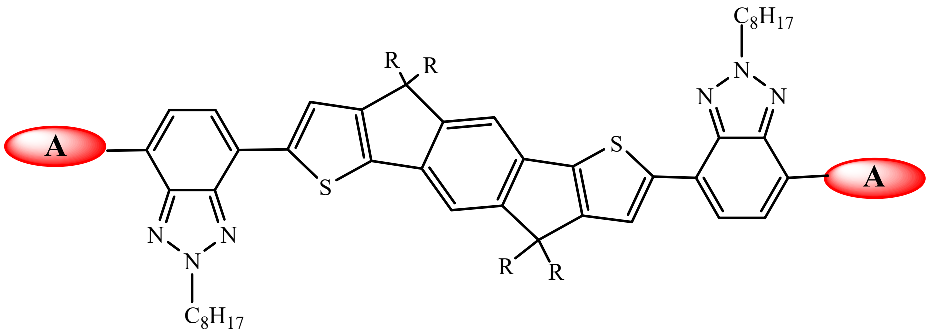

R= *p*-C_6H_4-C_6H_{13}

BTA1 BTA3 BTA7

A=

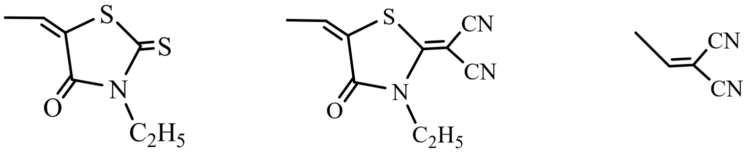

BTA11 BTA13 BTA17

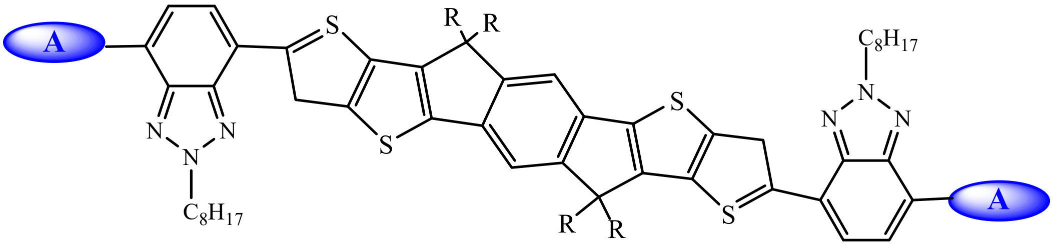

R= *p*-C_6H_4-C_6H_{13}

F-BTA3

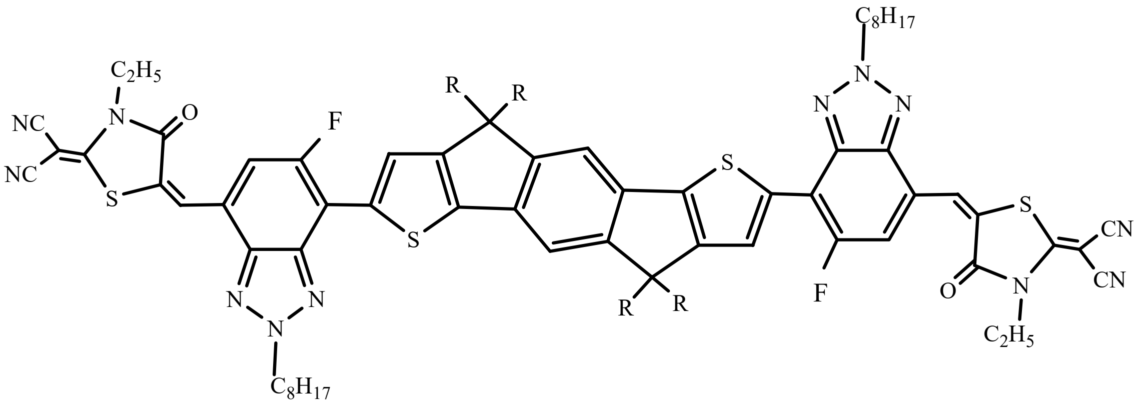

R=p-C_6H_{13}-C_6H_4

Y6, Y6DT

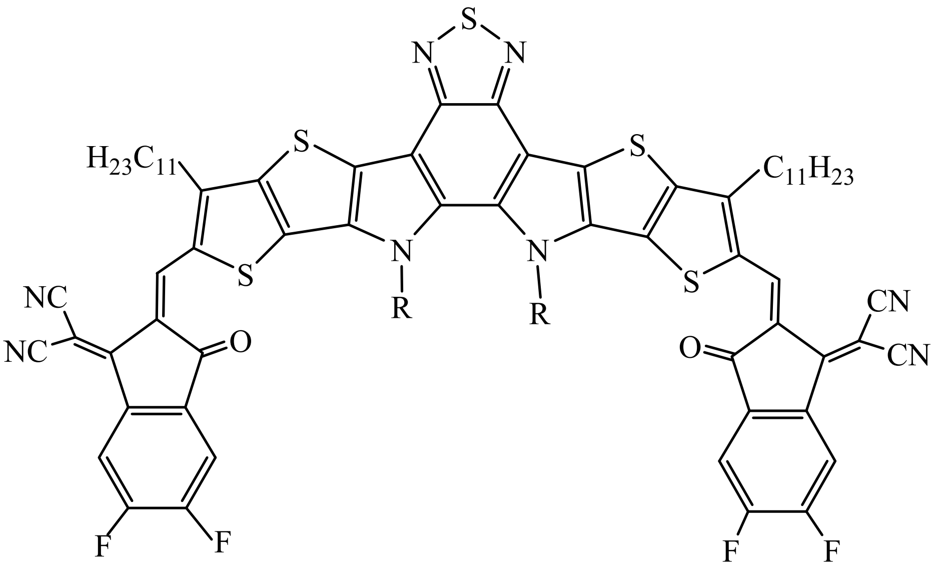

Y6 R=$CH_2CH(C_2H_5)C_4H_9$

Y6DT R=$CH_2CH(C_{10}H_{21})C_{12}H_{25}$

Y18

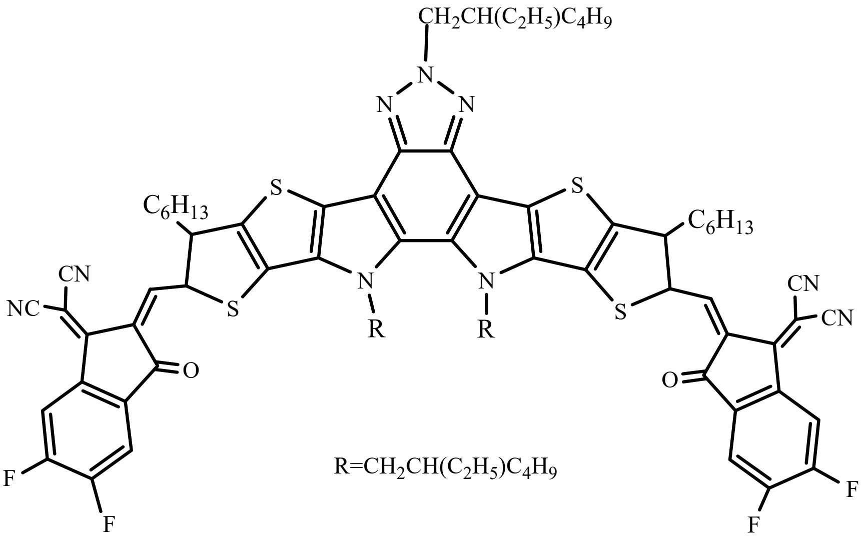

m-ITTC

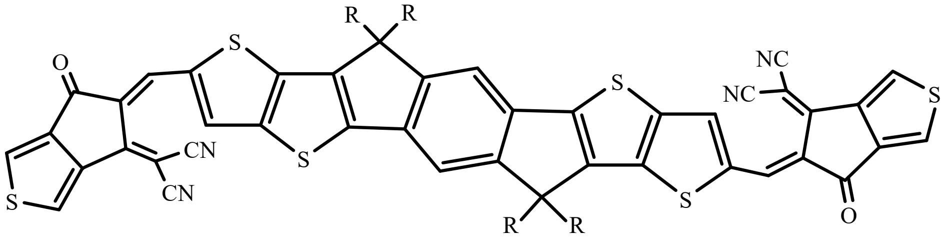

$R = p\text{-}C_6H_4\text{-}C_6H_{13}$

Y10

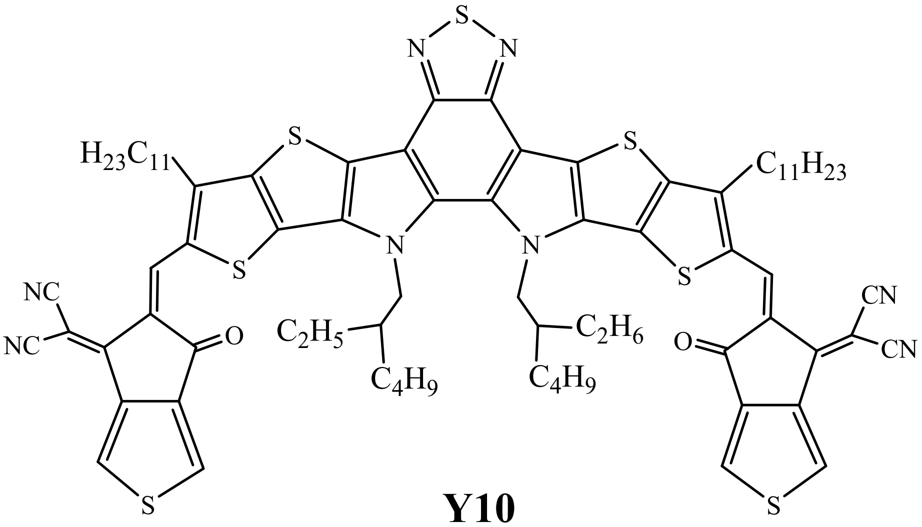

Y10

Y5

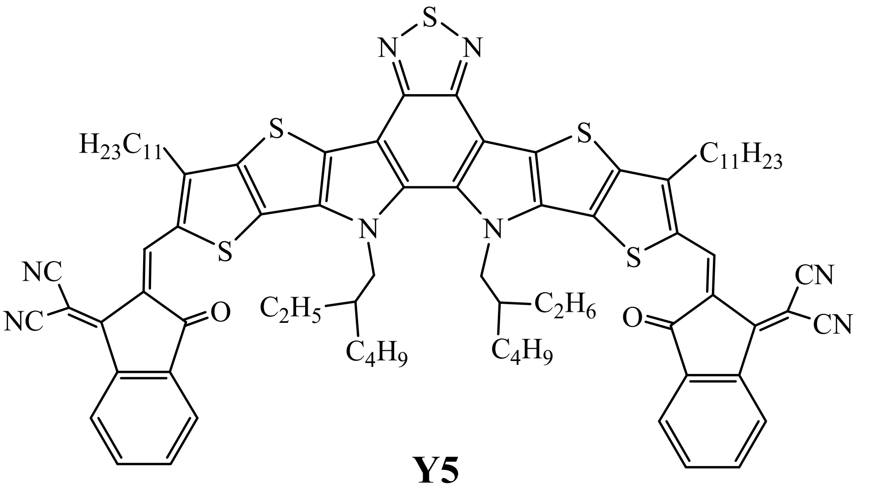

Y5

ITCPTC, MeIC

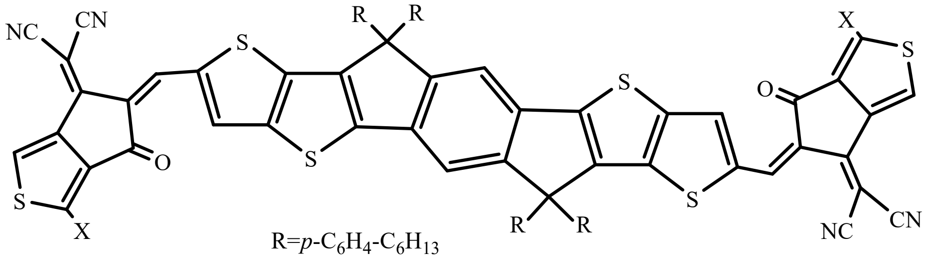

ITCPTC X=H
Me-IC X=F

ITC6-IC

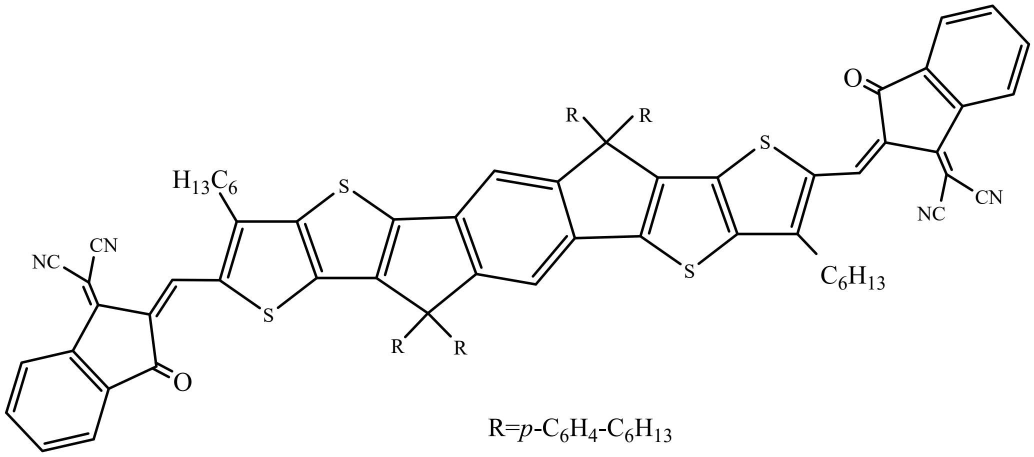

R=p-C_6H_4-C_6H_{13}

ITC6-IC

PNDICI

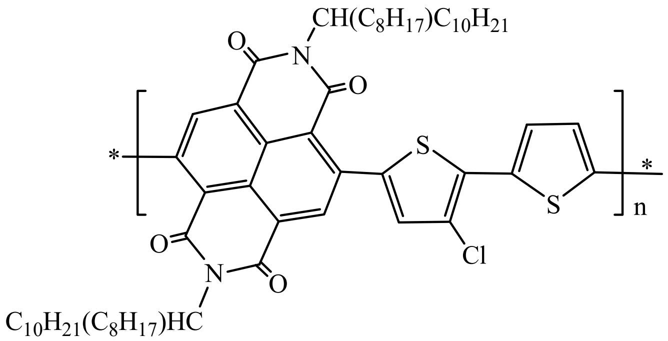

BTPT-4F

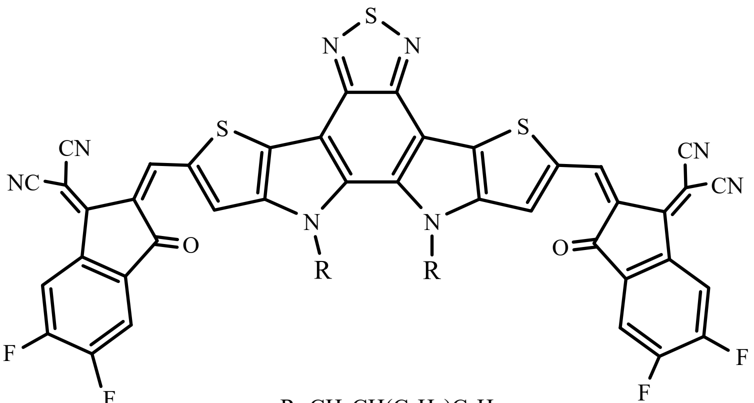

R=$CH_2CH(C_4H_9)C_6H_{13}$

PS1

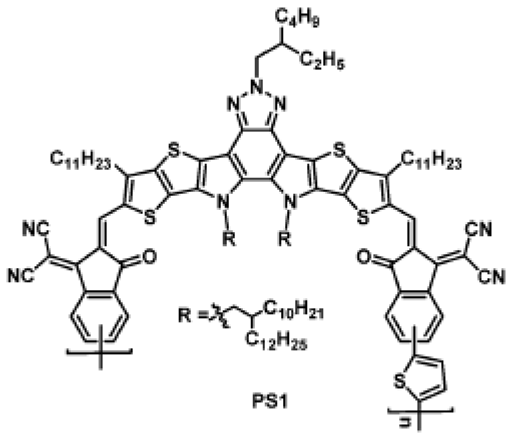
C4H9
C2H5
C11H23
C11H23
NC
NC
CN
CN
R = C10H21
C12H25
PS1

IHIC

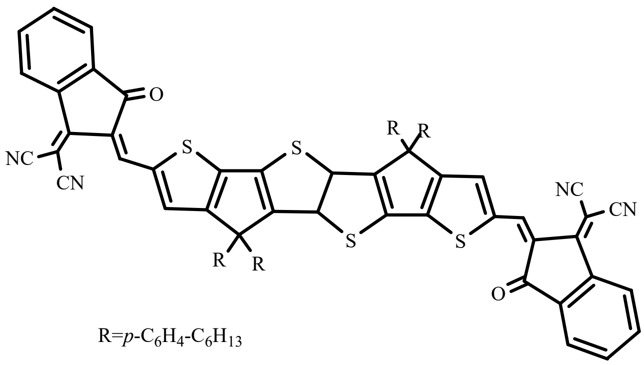
NC
CN
NC
CN
R=p-C6H4-C6H13

L8B0

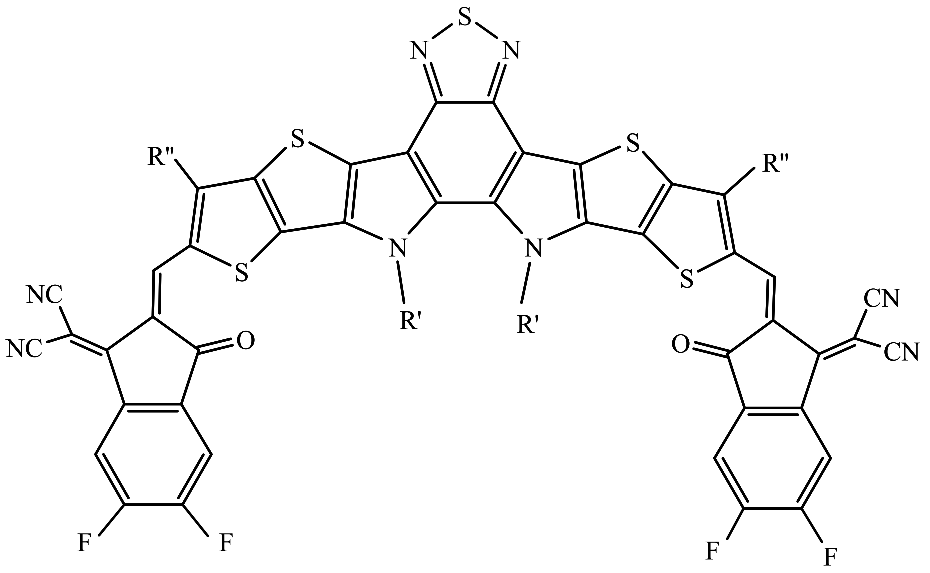

R'= $CH_2CH(C_2H_5)C_4H_9$
R"= $CH_2CH(C_4H_9)C_6H_{13}$

BTA75 e BTA76

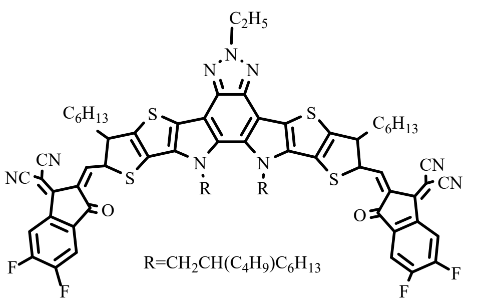

BTA75

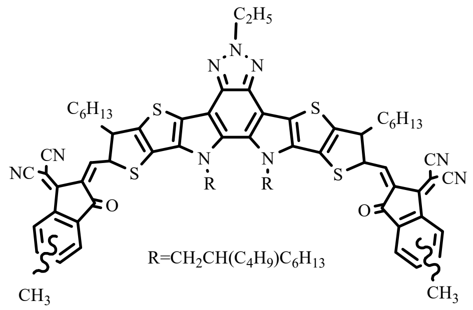

BTA76

Figure S2 Structural formula of donor polymer used with Bzt-based acceptor polymers in the text

PBDB-T

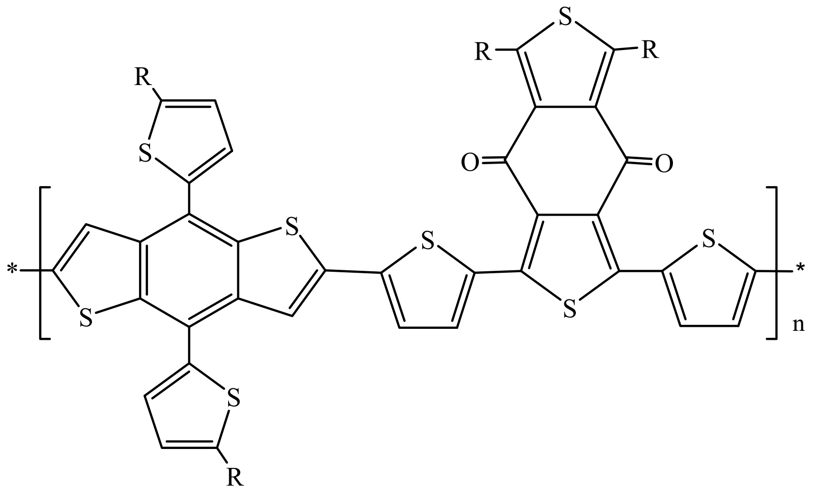

PM6

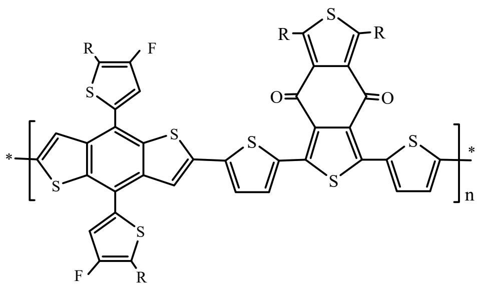

R= $CH_2CH(C_2H_5)C_4H_9$

Figure S3 . Structural formula of OTAZ

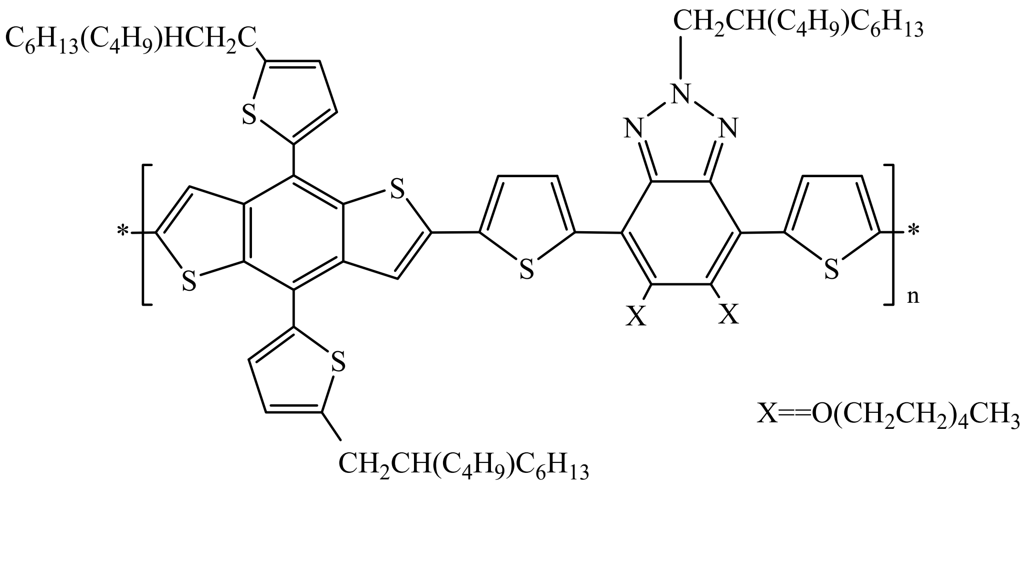

Figure S4 Structural formula of F OTAZ

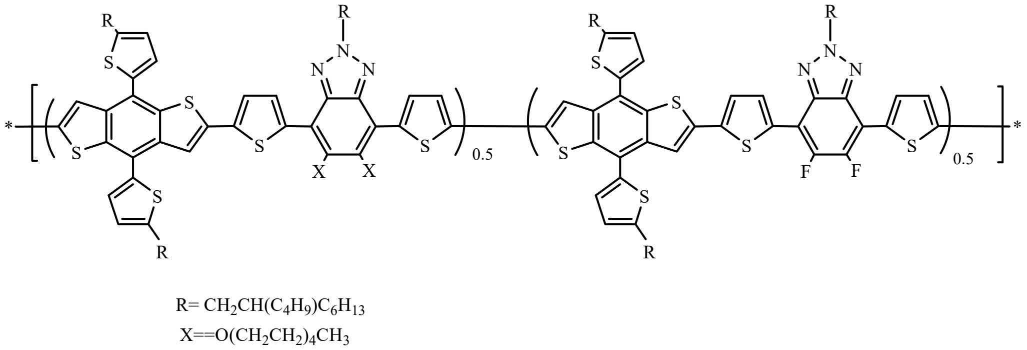

F-OTAZ

Figure S5 Structural formula of 4'-FT-FTAZ

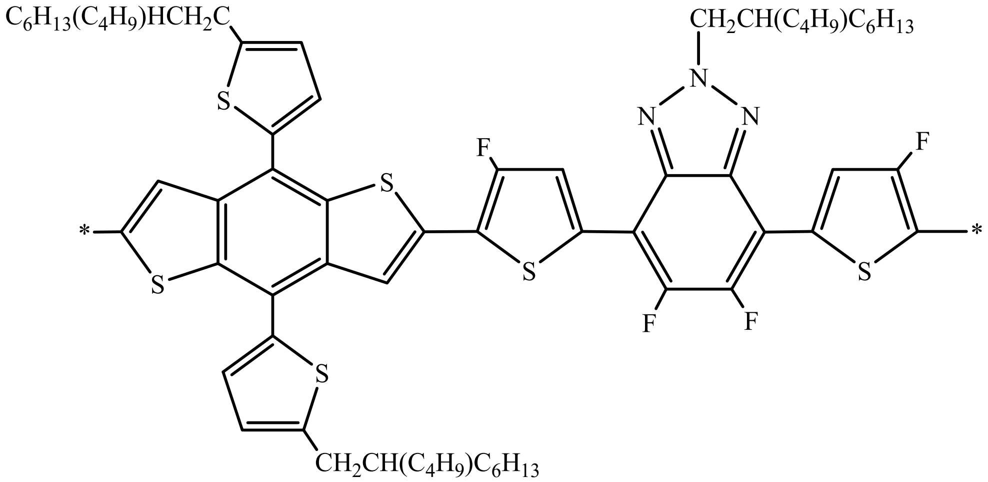

Figure S6 Structural formula of J52-based terpolymer (ref 51, ref 59).

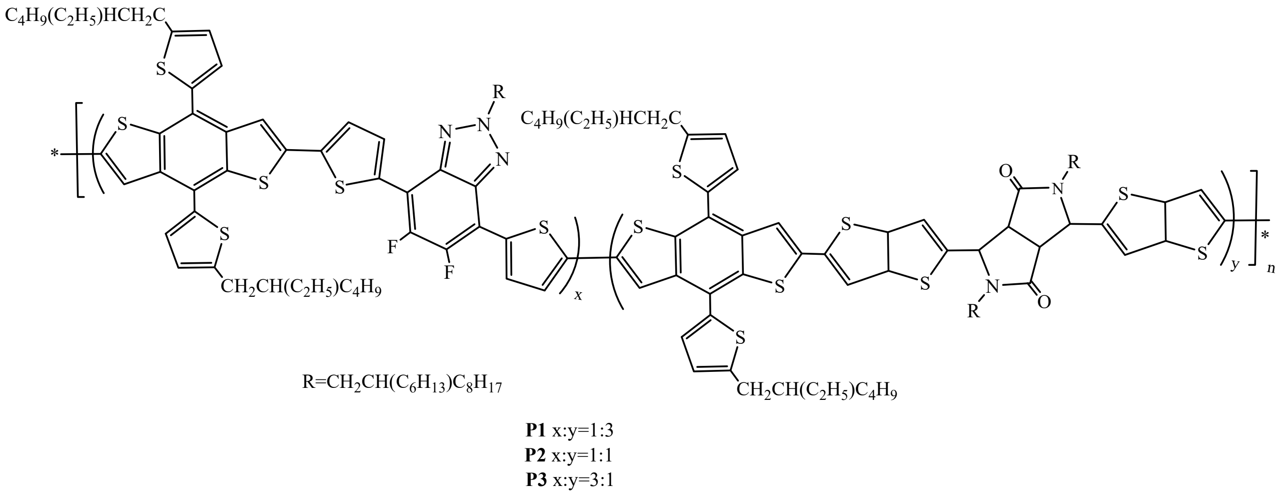

P1 x:y=1:3
P2 x:y=1:1
P3 x:y=3:1

Ref 51

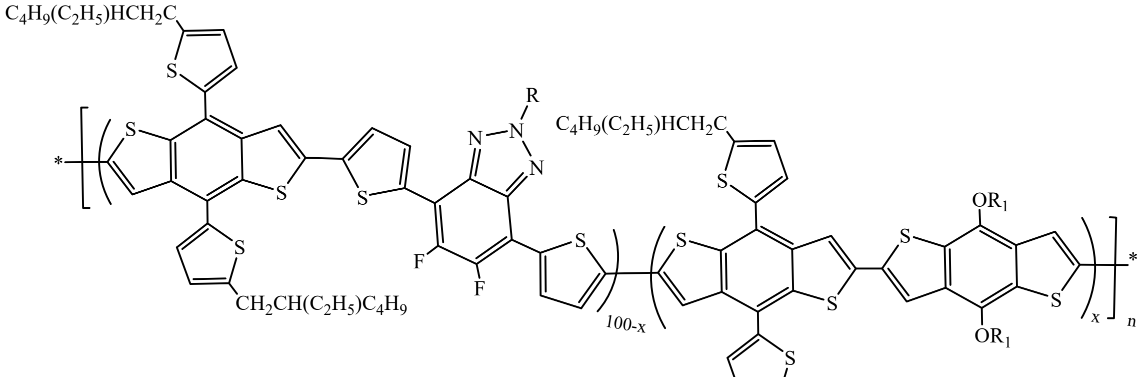

R=CH$_2$CH(C$_6$H$_{13}$)C$_8$H$_{17}$

R'=C$_{10}$H$_{23}$

PBDT(T)FTAZ x=0
PBDT(T)FTAZ-B$_5$ x=5
PBDT(T)FTAZ-B$_{10}$ x=10
PBDT(T)FTAZ-B$_{15}$ x=15

Ref 59

Figure S7 Structural formula of A) J55 and B) J52-OMe

A)

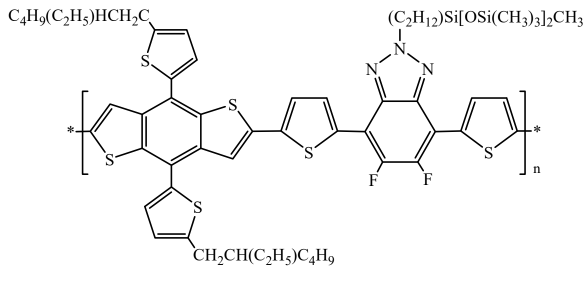

J55

B)

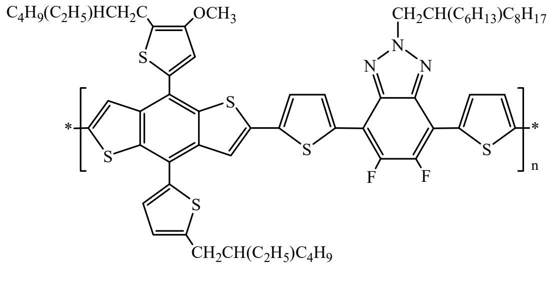

J52-OMe

Figure S8 Structural formula of PffBTT2-DPPT2

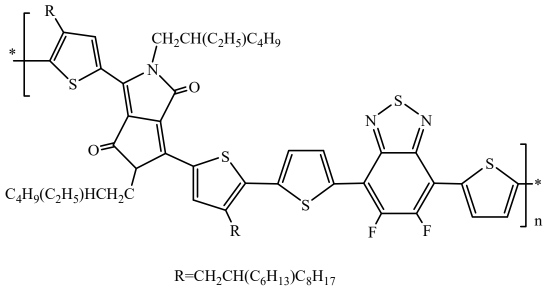

Figure S9 Structural formula of PTB7-Th

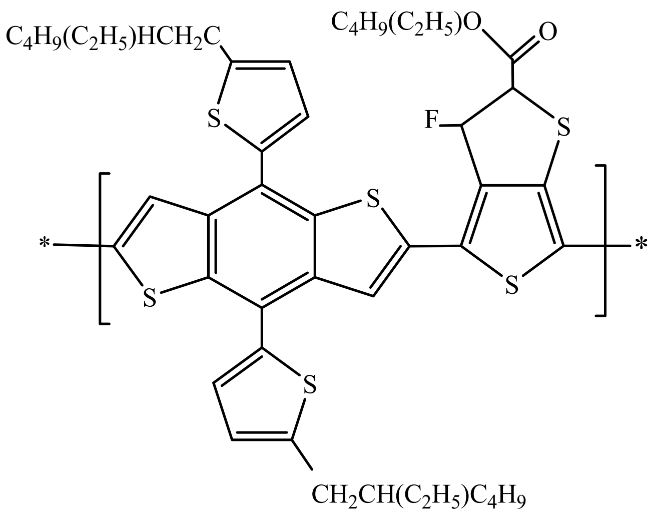

PTB7-Th

Figure S10 Structural formula of PCBO-12

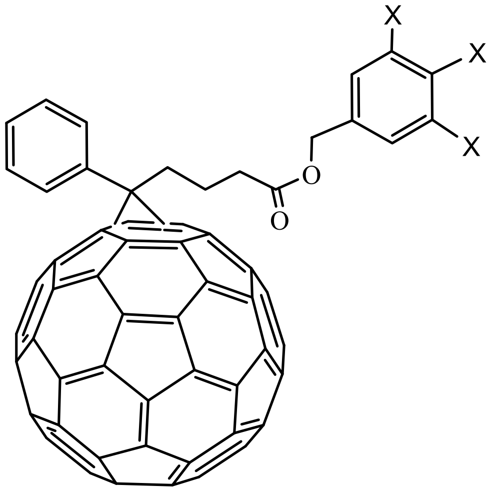

PCBO12 X=$(OCH_2CH_2)_3CH_3$
